# Carbon monoxide and hydrogen (syngas) as a C1-building block for selective catalytic methylation†

**DOI:** 10.1039/d0sc05404f

**Published:** 2020-11-20

**Authors:** Akash Kaithal, Markus Hölscher, Walter Leitner

**Affiliations:** Max Planck Institute for Chemical Energy Conversion Stiftstraße 34-36 Mülheim a.d. Ruhr 45470 Germany walter.leitner@cec.mpg.de; Institut für Technische und Makromolekulare Chemie, RWTH Aachen University Worringer Weg 2 52074 Aachen Germany

## Abstract

A catalytic reaction using syngas (CO/H_2_) as feedstock for the selective β-methylation of alcohols was developed whereby carbon monoxide acts as a C1 source and hydrogen gas as a reducing agent. The overall transformation occurs through an intricate network of metal-catalyzed and base-mediated reactions. The molecular complex [Mn(CO)_2_Br[HN(C_2_H_4_P*^i^*Pr_2_)_2_]] 1 comprising earth-abundant manganese acts as the metal component in the catalytic system enabling the generation of formaldehyde from syngas in a synthetically useful reaction. This new syngas conversion opens pathways to install methyl branches at sp^3^ carbon centers utilizing renewable feedstocks and energy for the synthesis of biologically active compounds, fine chemicals, and advanced biofuels.

## Abstract 2

A broadly applicable catalytic process for the selective β-methylation of alcohols is presented using syngas (CO/H_2_) directly as a C1 building block and the shown manganese complex in the presence of a base as the catalytic system.

## Graphical abstract

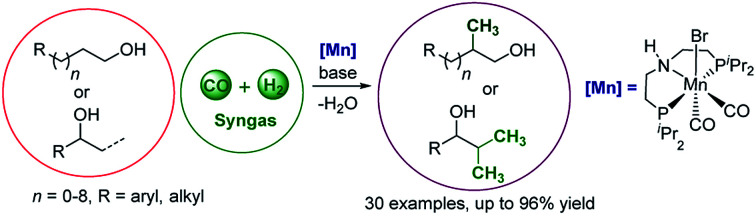

## Background and motivation

Synthesis gas (syngas), a mixture of carbon monoxide (CO) and hydrogen (H_2_), is a crucial relay between the energy and the chemical sector. While produced mainly from fossil resources today,^1,2^ it can be obtained also from other carbon feedstocks such as biomass,^3–5^ recycled plastics,^6^ or even carbon dioxide (CO_2_) combined with renewable energy.^7–9^ Therefore, catalytic processes for the chemical conversion of syngas are considered central elements in future sustainable chemical value chains. In particular, conversion of renewable-based syngas *via* the Fischer–Tropsch process^10–12^ or methanol synthesis^13–15^ is finding wide-spread interest due to the large product volumes. At the same time, “de-fossilized” syngas may be envisaged also as a C1-building block in later-stages of the chemical value chain.^16,17^ Hydroformylation, for example, uses syngas for the production of commodities and fine chemicals.^18–20^

**Scheme 1:**
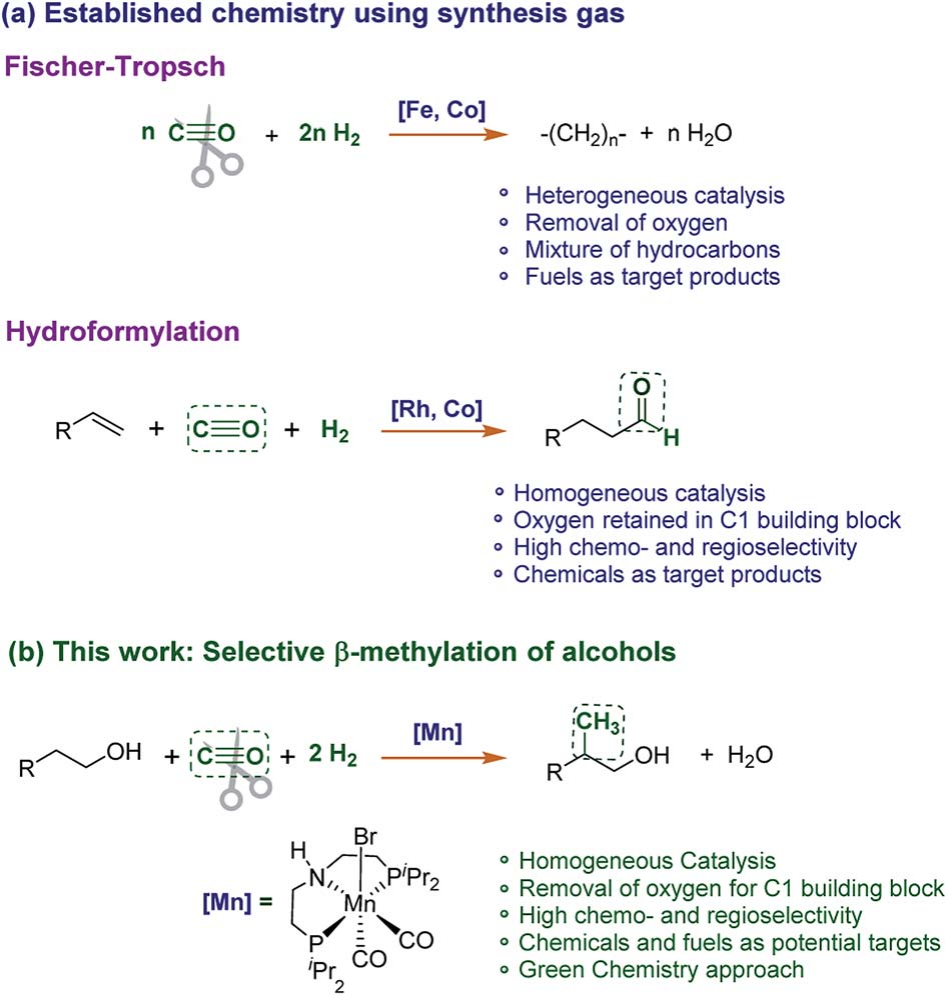
(a) Established processes for the conversion of syngas (CO/H_2_) to fuels and chemicals. (b) Novel catalytic reaction to use syngas as a methyl source for the β-methylation of alcohols.

We report here a novel catalytic process using syngas to install methyl branches (H_3_C–) with high selectivity at existing aliphatic carbon chains in the β-position of alcohol substrates (Scheme 1). This transformation combines two distinct features of Fischer–Tropsch chemistry (full deoxygenation of CO, hydrocarbon product) and hydroformylation (chemo- and regioselectivity, C1 building block). The reaction is catalysed by a molecular complex comprising earth abundant and non-toxic manganese as an active metal in the presence of a suitable base. The transformation opens new pathways to introduce renewable carbon into molecular structures with potential applications for the synthesis of fuels, large volume products, fine chemicals, and pharmaceuticals.

## Results and discussion

Most recently, catalytic methods using methanol (CH_3_OH) for β-methylation of alcohols have been reported by us^21–23^ and others.^24–29^ These reactions occur *via* an integrated “borrowing hydrogen” reaction sequence involving metal-catalyzed re-hydrogenation and de-hydrogenation in conjunction with base-mediated aldol-condensation/isomerization. Formaldehyde is formed as the C1 building block *in situ* by de-hydrogenation of methanol in these systems.^21–23^ Starting from syngas as the C1 source would thus require to provide sufficient concentrations of formaldehyde to enter this sequence. Notably, examples for catalytic generation of formaldehyde from syngas using homogeneous catalysis are very scarce.^30^ Lately, however, the groups of Prakash as well as Checinski and Beller reported the amine assisted hydrogenation of CO to methanol using homogeneous transition metal catalysts based on ruthenium and manganese, respectively.^31,32^

Based on recent progress using Mn-complexes in alkylation^22,29,33–36^ and CO/CO_2_ hydrogenation,^32,37^ we explored the potential of the well-established complex [Mn(^*i*^Pr-MACHO)(CO)_2_Br] (^*i*^Pr-MACHO = HN(C_2_H_4_P^*i*^Pr_2_)_2_; 1) as a catalyst precursor for the methylation directly from syngas. At the outset, 2-phenylethanol (2a) was selected as a benchmark substrate to validate the catalytic activity of 1 and to screen a set of parameters for optimization. The initial conditions involved reacting 2a under a mixture of CO (5 bar) and H_2_ (15 bar) in the presence of complex 1 (1 mol%) and NaO^*t*^Bu as a base (2 equiv. with respect to 2a) in toluene as a solvent. Already under this preliminary set of conditions, analysis of the liquid phase revealed >99% conversion of 2a and the formation of the β-methylated product 3a in a yield of 65% after 24 h at 150 °C (Table 1, entry 1). Alkenes and aldol-coupled products were observed in the reaction mixture indicating selectivity as the main optimization target. Using the closely related noble metal complex [RuH(CO)(BH_4_)(HN(C_2_H_4_PPh_2_)_2_)] as a catalyst resulted in rather unselective conversions with 25% yield of 3a only, highlighting the superior performance of the 3d metal in the diagonal position of the periodic table in this case.

**Table 1:** Mn^I^ catalyzed β-methylation of 2a with CO and H_2_: influence of different reaction conditions^a^

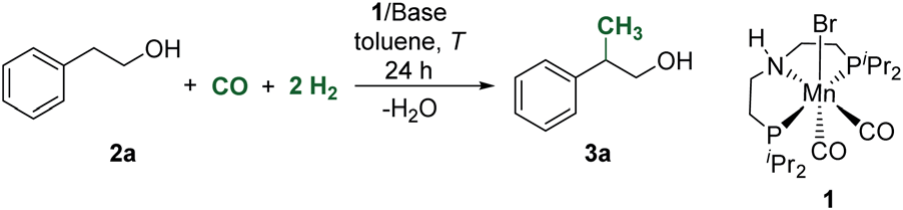						
	CO (bar)	H_2_ (bar)	Base (equiv.)	Temp. (°C)	Conv. (%)	Yield (%)
1^b^	5	15	NaO^*t*^Bu (2)	150	>99	65
2	5	15	NaO^*t*^Bu (2)	150	>99	92
3	5	15	NaO^*t*^Bu (2)	120	94	73
4	5	15	NaO^*t*^Bu (2)	170	>99	54
5	2.5	7.5	NaO^*t*^Bu (2)	150	72	15
6	8	24	NaO^*t*^Bu (2)	150	>99	93
7	5	15	NaO^*t*^Bu (1)	150	82	67
8	5	15	KO^*t*^Bu (2)	150	>99	0
9	5	15	Cs_2_CO_3_ (1)	150	8	0

a Reaction conditions: 2-phenylethanol 2a (0.5 mmol), Mn-complex 1 (2 mol%), CO, H_2_, base, and toluene (0.8 mL) were heated in a high-pressure reactor for 24 h. Conversion and yield were calculated using ^1^H NMR spectroscopy.

b Modified conditions: 2a (0.5 mmol), 1 (1 mol%), CO, H_2_, base, and toluene (0.8 mL) were heated in a high-pressure reactor for 24 h.

The selectivity for β-methylation could be improved significantly when the amount of complex 1 was increased to 2 mol%. The desired product 3a was formed with a selectivity of 92% at >99% conversion and isolated in 86% yield by column chromatography (Table 1, entry 2). Lower yields were obtained at lower (120 °C) as well as higher (170 °C) temperatures (Table 1, entry 3 and 4) reflecting a combined influence of activity and selectivity. Reducing the syngas pressure decreased the yield to the desired methylated product drastically while conversion remained relatively high (Table 1, entry 5). Increasing the syngas pressure (CO: 8 bar, H_2_: 24 bar) gave similar results as under the conditions of entry 2 (Table 1, entry 6). When the amount of NaO^*t*^Bu was decreased to 1 equiv. with respect to 2a, the rate of the transformation decreased leading to 3a in 67% yield at 82% conversion (Table 1, entry 7). Replacing the base with KO^*t*^Bu led to high conversion but very unselective product formation including polymeric materials, while Cs_2_CO_3_ resulted in no significant activity (Table 1, entry 8 and 9).

**Fig. 1:**
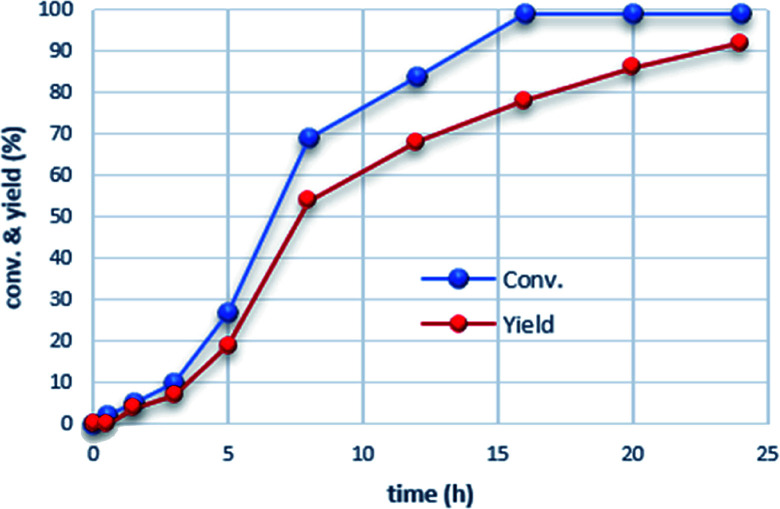
Conversion/time profile based on ^1^H NMR of individual experiments. Reaction conditions: 2a (0.5 mmol), Mn-complex 1 (2 mol%), CO (5 bar), H_2_ (15 bar), NaO^*t*^Bu (1 mmol), and toluene (0.8 mL) were heated at 150 °C in a high-pressure reactor for a certain time.

Monitoring the pressure over time for the β-methylation of 2-phenyl propanol 2a under the conditions of entry 2, Table 1, indicated sigmoidal reaction progress (see the ESI†). In the first 3 hours, the pressure dropped slowly by 2.5 bar followed by a sharp decrease resulting in a total 12 bar pressure drop over 7 h which continued to finally reach a stable value of a 15 bar total pressure drop after 16 h. The conversion/time profile obtained by the ^1^H NMR analysis of the reaction mixture at different time intervals corroborate this observation (Fig. 1). The reaction starts with a significant induction period exhibiting only 5% conversion and 4% yield to the β-methylated alcohol product after 1.5 h. The reaction rate continuously increases reaching a maximum around 50% conversion. Subsequently, the transformation slows down but continues to reach >99% conversion while selectivity catches up to result in 92% yield of the desired product 3a. The ^1^H NMR spectra revealed the formation of small amounts phenylacetaldehye (^1^H NMR = 9.86 ppm, *t*, *J* = 4 hz), methanediol (^1^H NMR = 4.89 ppm, s), and methanol (^1^H NMR = 3.50 ppm, s) as potential intermediate products in the reaction (see the ESI†).

**Scheme 2:**
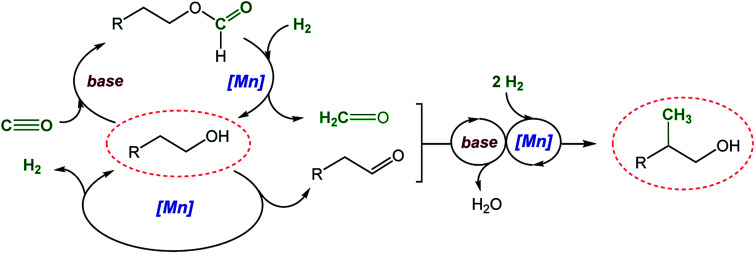
Proposed reaction network for the catalytic β-methylation of alcohols using syngas (CO/H_2_) as a C1 source.

**Scheme 3:**
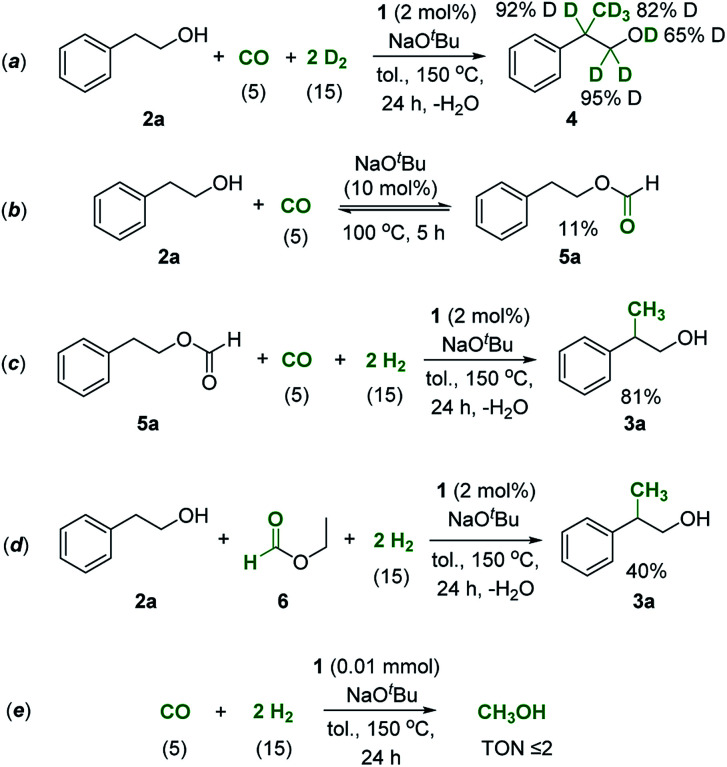
Control experiments to investigate the reaction sequence shown in Scheme 2. Values in parentheses correspond to the pressure of the reactive gases.

Scheme 2 shows a plausible reaction network to rationalize the basic mechanism of the new catalytic process which is consistent with the conversion/time profile of Fig. 1 and supported by the control experiments summarized in Scheme 3.^38^ The Mn–pincer complex catalyzed de- and re-hydrogenation steps of the organic substrate and intermediates^39–52^ and the base mediated aldol condensation are well established and the deuterium scrambling over all three carbon centers is fully consistent with this sequence (Scheme 3a).^22,36^ The high degree of deuterium incorporation at the α- and β-position in product 4 reflects rapid de- and re-hydrogenation at all stages of the catalytic network (Scheme 3a, see the ESI, Section 5.2 and 5.3†). The manganese-catalyzed generation of formaldehyde from CO/H_2_ is unprecedented, however, and unlocks the overall manifold. It can be reasonably assumed as the limiting factor in the initial phase explaining the observed induction period. While a direct hydrogenation pathway for CO reduction cannot be fully excluded, we favor an indirect conversion similar to previous reports on homogeneously catalyzed methanol formation from syngas.^31,32,53,54^ These reports have identified organic formyl species resulting from base mediated coupling of CO and secondary amines as crucial intermediates for CO hydrogenation. In full analogy, the alcohols used as substrates here can be converted to formate esters in the presence of CO and NaO^*t*^Bu.^53,55^ This possibility was confirmed for substrate 2a in the absence of hydrogen and a catalyst under otherwise typical reaction conditions (Scheme 3b). In line with this assumption, the reaction of 2-phenethyl formate 5a produced the methylated alcohol 3a in 81% yield under standard conditions (Scheme 3c). Furthermore, the reaction of 2a in the presence of ethyl formate and hydrogen led to the methylated product formation with a yield of 40% (Scheme 3d), clearly demonstrating that formate esters can serve as a formaldehyde source. On the other hand, when syngas was reacted under the standard conditions but in the absence of alcohol, the reaction resulted in only a very small amount of methanol (TON ≤ 2) and no formation of formaldehyde or methanediol was detected under these conditions (Scheme 3e). These control experiments affirmed that the presence of the alcohol substrate is necessary to mediate the hydrogenation of carbon monoxide most likely *via* formate esters as intermediates.

**Scheme 4:**
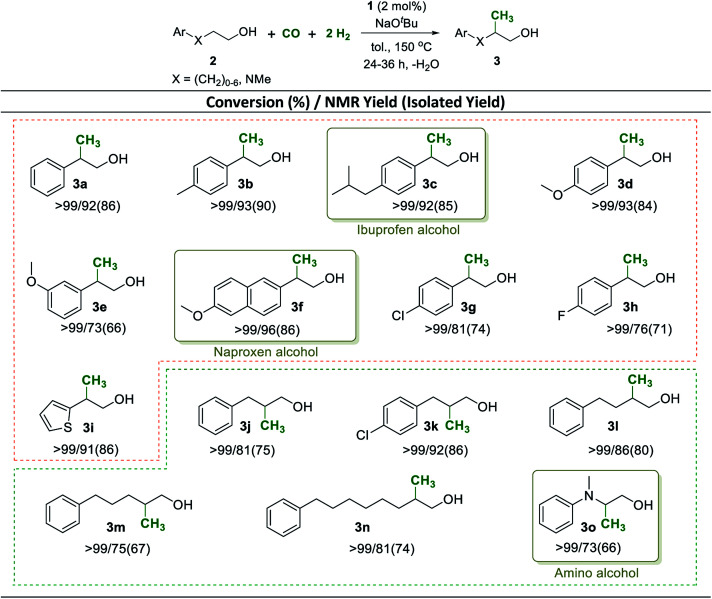
| Mn^I^ catalyzed β-methylation of aryl substituted alcohols 2 with CO and H_2_. Reaction conditions: 2 (0.5 mmol), Mn-complex 1 (2 mol%), CO (5 bar), H_2_ (15 bar), NaO^*t*^Bu (1 mmol), and toluene (0.8 mL) were heated at 150 °C in a high-pressure reactor for 24 h. Conversion and yield were calculated using ^1^H-NMR spectroscopy. Yields in parentheses correspond to isolated yield.

Having established a robust method to construct a methyl group from syngas in the β-position of the aliphatic chain in the benchmark substrate 2a, we set out to explore the synthetic scope of this catalytic reaction. The methyl branch is a highly important structural motif in biologically active products such as pharmaceuticals and agrochemicals.^56,57^ Using the reaction conditions of Table 1 entry 2 as standard procedure, various 2-arylethanol derivatives were methylated with CO and H_2_ addressing potential building blocks and intermediates for biologically active products (Scheme 4, 3a–3i).

Electron donating as well as withdrawing substituents in the phenyl ring were fully tolerated and no dehalogenation was observed (Scheme 4, 3a–3h). Notably, pharmaceutically relevant ibuprofen and naproxen alcohols were prepared by using this new methodology in excellent yields of 92% and 96%, respectively (Scheme 4, 3c and 3f). The sulfur containing heterocyclic 2-(thiophen-2-yl)ethanol was converted effectively and the product 3i could be isolated in 86% yield. Aryl-substituted longer chain aliphatic alcohols reacted also smoothly under standard conditions providing very good to excellent yields of the corresponding methyl-branched products (Scheme 4, 3j–3n). Again, heteroatoms were tolerated and the pharmaceutically important amino alcohol 2-(methyl(phenyl)amino)ethan-1-ol^58^ was selectively mono-methylated with a yield of 73% at >99% conversion (Scheme 4, 3o).

**Scheme 5:**
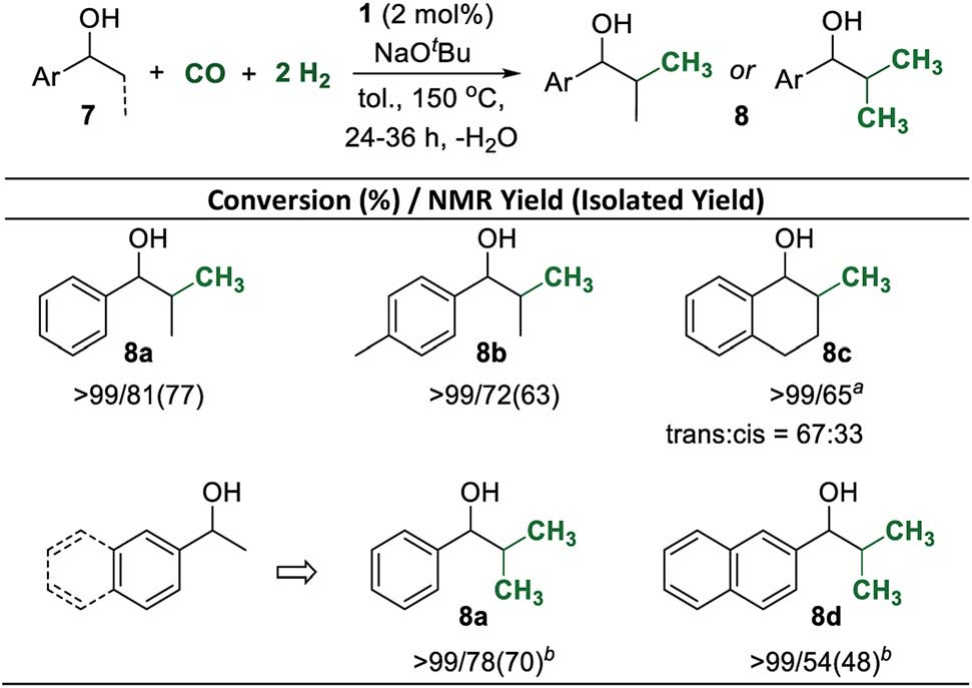
Mn^I^ catalyzed β-methylation of secondary alcohols 7 with CO and H_2_. Reaction conditions: same as Scheme 4. ^*a*^Modified conditions: 7 (0.5 mmol), Mn-complex 1 (2 mol%), CO (5 bar), H_2_ (15 bar), NaO^*t*^Bu (1 mmol), and toluene (0.8 mL) were heated in a high pressure reactor for 36 h. ^*b*^7 (0.5 mmol), Mn-complex 1 (2 mol%), CO (8 bar), H_2_ (24 bar), NaO^*t*^Bu (2 mmol), and toluene (0.8 mL) were heated in a high-pressure reactor for 36 h.

Secondary alcohols of general structure 7 were used also as substrates for selective β-methylation (Scheme 5). Using the standard reaction conditions, 1-phenylpropan-1-ol was mono-methylated at the β-position with 81% selectivity at full conversion and the product was isolated in 77% yield (Scheme 5, 8a). Similarly, 1-(*p*-tolyl)propan-1-ol was also mono-methylated in good yield (Scheme 5, 8b). The reaction also occurred readily when the reactive position was part of a 6-membered carbocycle, installing the methyl group with a 2 : 1 preference in the *trans*-position to the OH group (Scheme 5, 8c). Di-methylation of 1-arylethanols to generate *iso*-propyl groups was possible under slightly adjusted conditions (see the ESI†). Increasing the amount of base to 4 equiv. and reacting the substrates under higher pressures of CO (8 bar) and H_2_ (24 bar) for 36 h allowed isolation of the di-methlyated products 8a and 8d in 70% and 48%, respectively.

**Scheme 6:**
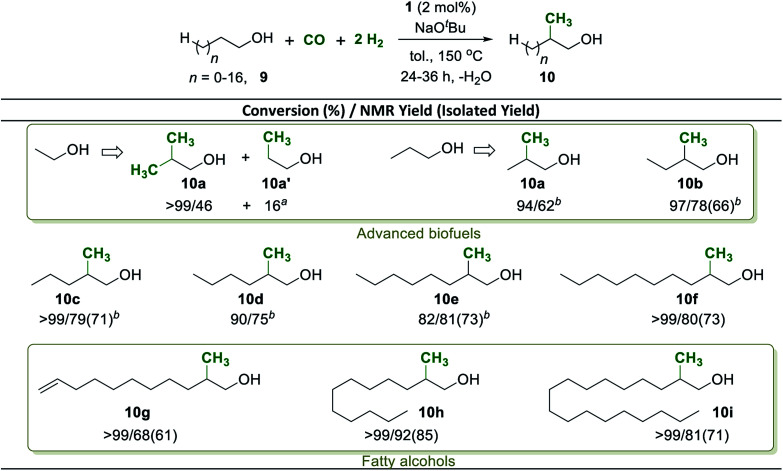
Mn^I^ catalyzed β-methylation of aliphatic alcohols 9 with CO and H_2_. Reaction conditions: same as Scheme 4. ^*a*^Modified conditions: 9 (0.5 mmol), Mn-complex 1 (2 mol%), CO (8 bar), H_2_ (24 bar), NaO^*t*^Bu (2 mmol), and toluene (0.8 mL) were heated in a high-pressure reactor at 150 °C for 36 h. ^*b*^9 (0.5 mmol), Mn-complex 1 (2 mol%), CO (5 bar), H_2_ (15 bar), NaO^*t*^Bu (1 mmol), and toluene (0.8 mL) were heated at 150 °C in a high-pressure reactor for 36 h.

Methyl branches in aliphatic carbon chains feature beneficial combustion properties in fuel components.^59,60^ As a possible synthetic pathway for the upgrading of biogenic alcohols to fuel components with improved combustion properties,^61,62^ we therefore focused next on the selective β-methylation of purely aliphatic alcohols (Scheme 6). Ethanol proved to be a challenging substrate, but a mixture of *iso*-butanol (46%) from di-methylation and 1-propanol (16%) from mono-methylation was obtained under 8 bar of CO and 24 bar of H_2_ at an elongated reaction time of 36 h (Scheme 6, 10a). Longer chain aliphatic alcohols that can result in mono-methylation only were almost quantitatively converted under 5 bar of CO and 15 bar of H_2_ within 24–36 h providing good selectivity and yields (Scheme 6, 10b–10f). Fatty alcohols including lauryl alcohol, and stearyl alcohol were also converted resulting in high yield to the corresponding β-monomethylated alcohols (Scheme 6, 10h, and 10i). Remarkably, the unsaturated fatty alcohol undec-10-en-1-ol was transformed into the corresponding β-methylated product with 68% yield leaving the C=C double bond in the molecule intact (Scheme 6, 10g). These products may have potential application as fine chemicals for surfactants or in the cosmetic industry.

## Conclusions

In conclusion, a catalytic reaction has been developed employing syngas as raw material for the catalytic β-methylation of alcohols enabling the use of carbon monoxide as a renewable C1 source and “green” hydrogen as a reducing agent. The catalyst system comprises the earth-abundant, first row transition metal manganese in the form of an air-stable pincer complex as the metal component. This new catalytic reaction for the installation of methyl groups at sp^3^ C-centers generates water as the sole by-product. The reaction shows a remarkable broad substrate scope providing very high to excellent yields for primary and secondary alcohols. Potential products include fuel components or commodity chemicals, fine chemicals, and even pharmaceuticals. Even with the resource basis of today's petrochemical industry, the catalytic process described herein opens new retrosynthetic pathways to important target molecules providing potential environmental benefits. Most intriguingly, however, the resulting novel synthetic strategies may help to unlock the potential of waste, biomass, or CO_2_ as carbon sources for the chemical value chain in line with the principles of Green Chemistry.^17^ The general concept to access formaldehyde from syngas through a catalytic cycle with a molecularly defined organometallic complexes and to intercept this useful building block provides a multitude of further opportunities for chemical synthesis.

## Conflicts of interest

There are no conflicts to declare.

## Supplementary Material

SC-012-D0SC05404F-s001
