# Supplementary material for: Carbon monoxide and hydrogen (syngas) as a C1-building block for selective catalytic methylation
Source: Chem Sci. 2020 Nov 20;12(3):976–82. doi: 10.1039/d0sc05404f (PMC8179066; doi:10.1039/d0sc05404f)
Supplement: SC-012-D0SC05404F-s001 [file SC-012-D0SC05404F-s001.pdf]

Electronic Supplementary Material (ESI) for Chemical Science.
This journal is © The Royal Society of Chemistry 2020

Supporting Information

Carbon Monoxide and Hydrogen (Syngas) as C1-Building Block for Selective Catalytic Methylation

Akash Kaithal,[a,b] Markus Hölscher[b] and Walter Leitner[a,b]*

[a]Max Planck Institute for Chemical Energy Conversion, Stiftstraße 34-36, 45470 Mülheim a.d. Ruhr.

[b]Institut für Technische und Makromolekulare Chemie, RWTH Aachen University Worringer Weg 2, 52074 Aachen, Germany.

Corresponding Author: walter.leitner@cec.mpg.de
Leitner@itmc.rwth-aachen.de

Table of Contents

1. General Methods 2
2. Synthesis of Manganese pincer complex 1 2
3. Standard procedure and reaction optimization 3
4. Conversion/time profile for the β-methylation of 2a using Syngas at different time intervals 4
5. Control experiments to investigate the proposed reaction network 7
   - 5.1. Reaction of 2-phenylethan-1-ol 2a with with CO and D_2 using 1 7
   - 5.2. Reaction of 2-phenylethan-1-ol 2a with D_2 under standard reaction conditions 9
   - 5.3. Reaction of 2-phenylpropan-1-ol 3a with D_2 under standard reaction conditions 10
   - 5.4. Reaction of 2-phenylethan-1-ol 2a with carbon monoxide in the presence of a base 11
   - 5.5. Reaction of 2-Phenethyl formate 5a with CO and H_2 using 1 12
   - 5.6. Reaction of butyl formate 5b with CO and H_2 using 1 13
   - 5.7. Reaction of 2a with ethyl formate 6 and hydrogen 14
   - 5.8. Reaction of CO with H_2 using complex 1 15
6. Standard procedure for the catalytic β-methylation of aryl substituted alcohols 17
7. Standard procedure for catalytic β-monomethylation of secondary alcohols 24
8. Standard procedure and reaction optimization for catalytic β-dimethylation of 1-phenylethanol... 26
9. Standard procedure for catalytic β-dimethylation of secondary alcohols 26
10. Standard procedure for catalytic β-methylation of aliphatic alcohols 28
11. Postulated Catalytic Sequence 32
12. Alternate reaction mechanism 32
13. NMR spectra of isolated products 33
14. ^{1}H NMR reaction mixture spectra of β-methylated products 58

References 79

1. General Methods

All catalytic and stoichiometric reactions were performed under argon atmosphere using standard schlenk and glove box techniques. Glassware was dried under vacuum at high temperatures, evacuated and refilled with argon at least three times. The solvents were purified using solvent purification systems and were stored and handled under argon. Chemicals were purchased from Sigma-Aldrich, Alfa-Aesar, abcr, Acros Organics, TCI chemicals and used without further purification. NMR-spectra were recorded on Bruker AV-300, AV-400, DPX-300, AV-600 spectrometers at the indicated temperatures with the chemical shifts (δ) given in ppm relative to TMS and the coupling constants (J) in Hz. The solvent signals were used as references and the chemical shifts converted to the TMS scale (Acetonitrile-d_3: δ_H = 1.94 ppm and δ_C = 118.3, 1.3 ppm; $CDCl_3$: δ_H = 7.26 ppm and δ_C = 77.1 ppm; CD_2Cl_2: δ_H = 5.32 ppm and δ_C = 54.00 ppm; C_6D_6: δ_H = 7.16 ppm and δ_C = 128.1 ppm; THF-d_8: δ_H = 1.72 ppm, 3.58 ppm and δ_C = 67.2 ppm, 25.3 ppm; toluene-d_8: δ_H = 2.08, 6.97, 7.01, 7.09 ppm and δ_C = 137.5, 128.9, 127.9, 125.1, 20.4 ppm; DMSO-d_6: δ_H = 2.50 ppm and δ_C = 39.5ppm).[1]

Note: Catalytic reactions involving pressurized carbon monoxide and hydrogen were carried out in home built stainless steel reactors equipped with pressure transducer and external electrical heating.

Safety advice: High-pressure experiments represent a significant risk and must be conducted with appropriate safety procedures and in conjunction with the use of suitable equipment.

2. Synthesis of Manganese pincer complex 1

The manganese pincer complex **1** was synthesized according to the reported literature.[2] A solution of

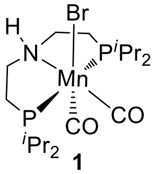

1

Bis(2-(diisopropylphosphaneyl)ethyl)amine (2.26 mL, 10 wt. % in THF, 0.655 mmol, 1.1 equiv.) was added to a solution of [$Mn(CO)_5Br$] (163.7 mg, 0.595 mmol, 1.0 equiv.) in toluene (8 mL) and stirred for 24 h at 100 °C under argon atmosphere. The volatiles were removed in vacuo and the residue was washed with pentane (3 x 5 mL). Upon drying in vacuo, the residue was solidified and complex **1** was obtained as a bright yellow powder (267.4 mg, 0.54 mmol, 91%). The analytical data of complex are consistent with those previously reported in the literature.[2]

3. Standard procedure and reaction optimization

Mn-MACHO-^{i}Pr **1** and NaO^{t}Bu were measured into a glass inlet equipped with a stirring bar inside a glovebox. The glass inlet was closed with a septum and transferred into the bottom part of the 10 mL steel autoclave, where it was opened under a stream of argon. After sealing, the autoclave was evacuated and purged with argon three times. 2-phenylethan-1-ol **2a** (61.0 mg, 0.5 mmol) and toluene (0.8 mL) were added at room temperature through a valve under argon. The autoclave was sealed, pressurized with CO and H_2 and heated at certain temperature for 24 h. After completion of the reaction, the autoclave was cooled to room temperature and slowly vented while stirring continued. Mesitylene was added as an internal standard to the reaction mixture that was then passed through a short path of acidic alumina before the composition was analyzed by NMR spectroscopy.

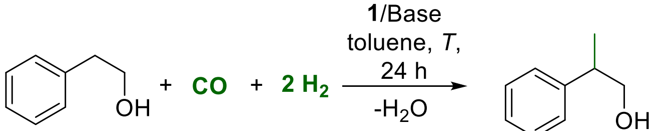

2a **3a**

Table S1: Optimization reactions for β–methylation of **2a** using **1**.

Entry	1 (mol%)	CO (bar)	H_2 (bar)	Base (equiv.)	Temp. (°C)	Conv. (%)[b]	Yield (%)[a]
1	1	5	15	NaO^{t}Bu (2)	150	>99	65
2	2	5	15	NaO^{t}Bu (2)	150	>99	92
3	2	5	15	NaO^{t}Bu (2)	120	94	73
4	2	5	15	NaO^{t}Bu (2)	170	>99	54
5	2	2.5	7.5	NaO^{t}Bu (2)	150	72	15
6	2	8	24	NaO^{t}Bu (2)	150	>99	93
7	2	5	15	NaO^{t}Bu (1)	150	82	67
8	2	5	15	KO^{t}Bu (2)	150	>99	0
9	-	5	15	NaO^{t}Bu (2)	150	>99	0
10	-	5	15	-	150	4	0
11	2	5	15	NaO^{t}Bu (0.1)	150	5	0

4. Conversion/time profile for the β-methylation of 2a using Syngas at different time intervals

Ten individual reactions were performed for the reaction progress experiments at different time intervals. Mn-MACHO-^{i}Pr **1** (4.95 mg, 2 mol%) and NaO^{t}Bu (96.1 mg, 1 mmol) were measured into a glass inlet equipped with a stirring bar inside a glovebox. The glass inlet was closed with a septum and transferred into the bottom part of the 10 mL steel autoclave, where it was opened under a stream of argon. After sealing, the autoclave was purged with argon three times. 2-phenyl ethanol **2a** (61.0 mg, 0.5 mmol) and toluene (0.8 mL) were added at room temperature through a valve under argon. The autoclave was sealed, pressurized with CO (5 bar) and H_2 (15 bar) and heated to 150 °C temperature for 24 h. After completion of the reaction, the autoclave was cooled to room temperature and slowly vented while stirring continued. Mesitylene (43.2 mg, 0.36 mmol) was added as an internal standard to the reaction mixture that was then passed through a short path of acidic alumina before the composition was analyzed by NMR spectroscopy. The profile is shown in the manuscript which corresponds to Figure 1.

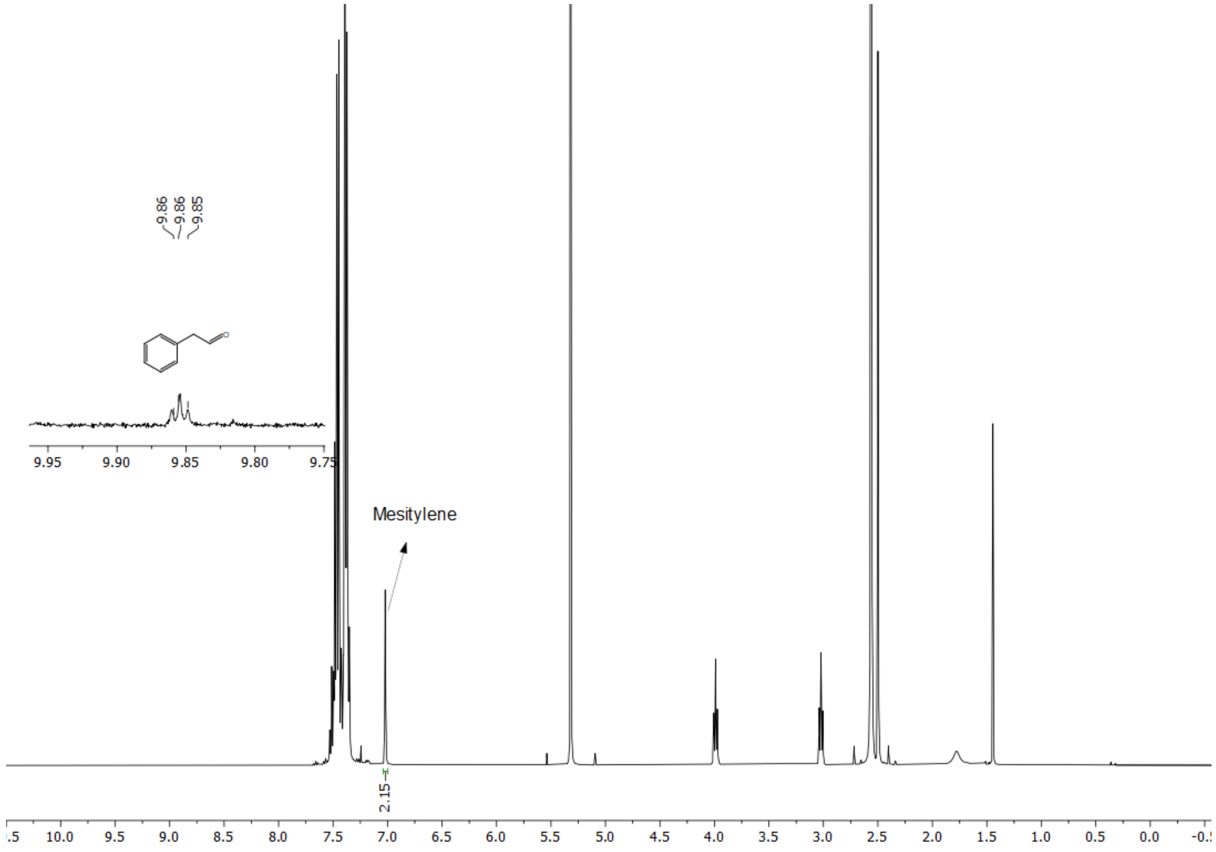

Figure S1: ^{1}H NMR (400 MHz, $CDCl_3$, 298 K) reaction mixture spectrum of 2a with syngas after 30 minutes as example.

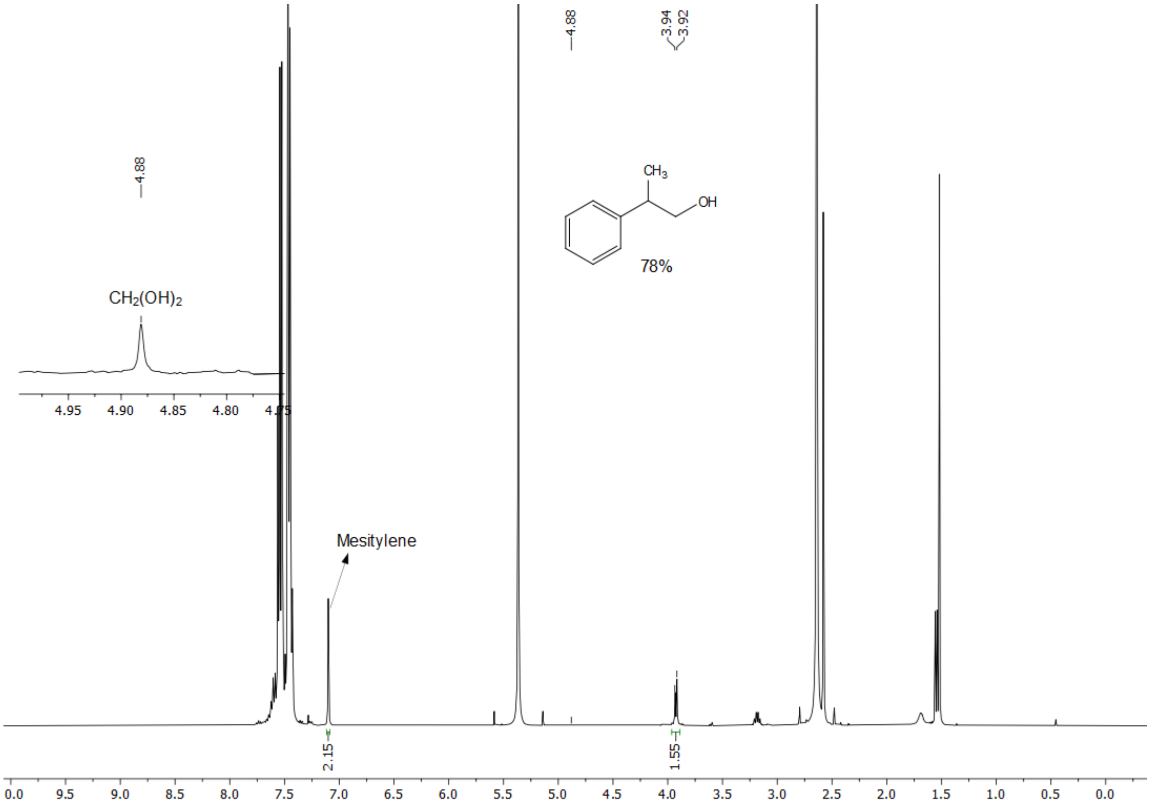

Figure S2: ^{1}H NMR (400 MHz, $CDCl_3$, 298 K) reaction mixture spectrum of 2a with syngas after 16 h as example.[3]

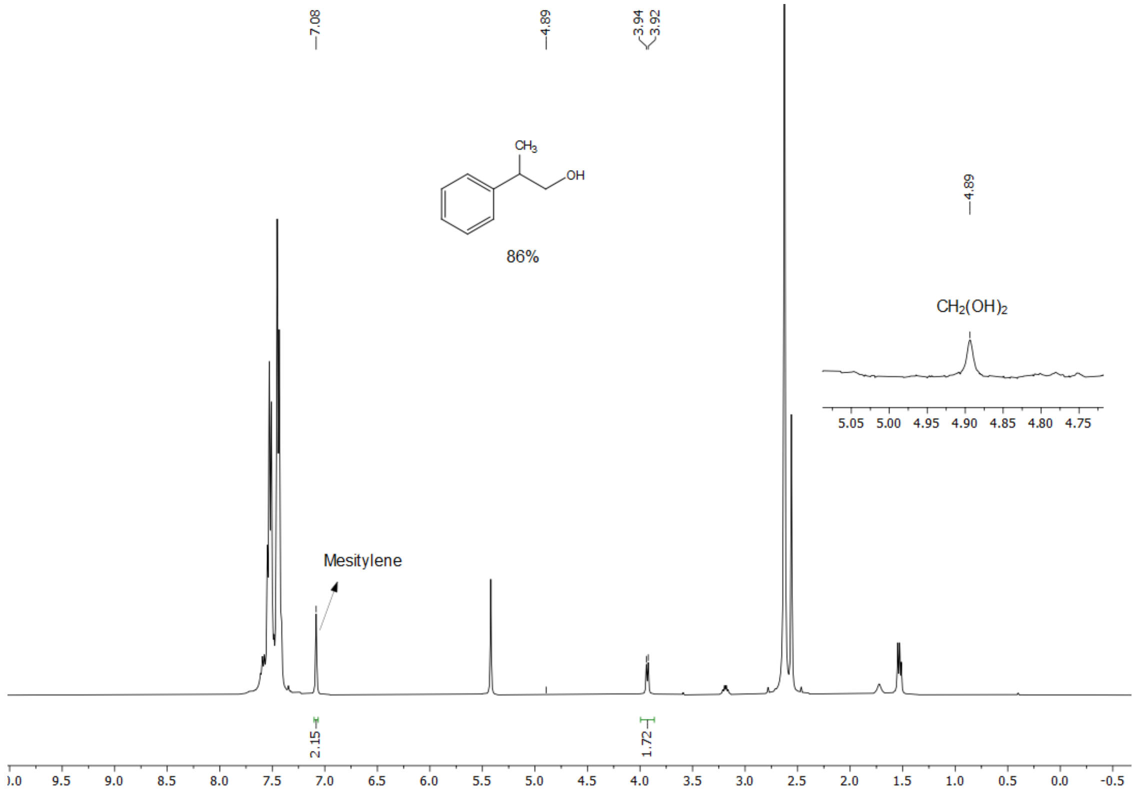

Figure S3: ^{1}H NMR (400 MHz, $CDCl_3$, 298 K) reaction mixture spectrum of 2a with syngas after 20 h as example.

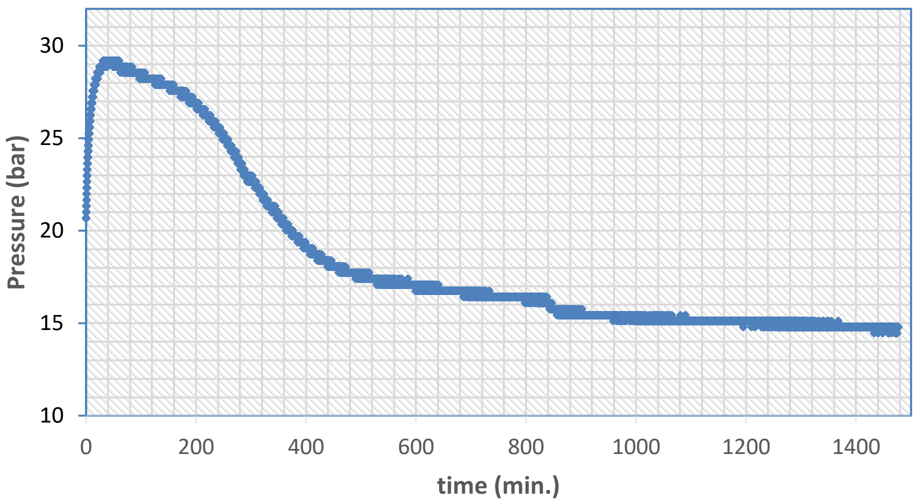

Figure S4: Pressure curve for the β-methylation of 2a using syngas.

5. Control experiments to investigate the proposed reaction network

5.1. Reaction of 2-phenylethan-1-ol 2a with with CO and D_2 using 1

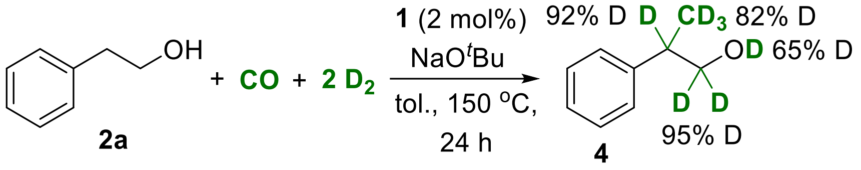

Mn-MACHO-^{i}Pr **1** (4.95 mg, 2 mol%) and NaO^{t}Bu (96.10 mg, 1 mmol) were measured into a glass inlet equipped with a stirring bar inside a glovebox. The glass inlet was closed with a septum and transferred into the bottom part of the 10 mL steel autoclave, where it was opened under a stream of argon. After sealing, the autoclave was evacuated and purged with argon three times. 2-phenylethan-1-ol **2a** (61.0 mg, 0.5 mmol) and toluene (0.8 mL) were added at room temperature through a valve under argon. The autoclave was sealed, pressurized with CO (5 bar) and D_2 (15 bar) and heated to 150 °C temperature. After 24 h, the autoclave was cooled to room temperature and slowly vented while stirring continued. The reaction mixture was passed through a short path of acidic alumina before the composition was analyzed by NMR spectroscopy.

^{1}H NMR (400 MHz, $CDCl_3$, 298 K) δ = 7.25-7.38 (m, 5H, ArC*H*), 3.72 (br s, 0.11H, CH_2), 2.96-2.97 (m, 0.08H, C*H*), 1.42 (br s, 0.35H, O*H*), 1.27 (br s, 0.53H, CH_3).

^{2}H NMR (61 MHz, $CDCl_3$, 298 K) δ = 3.72 (br s), 2.94 (br m), 1.27 (br s).

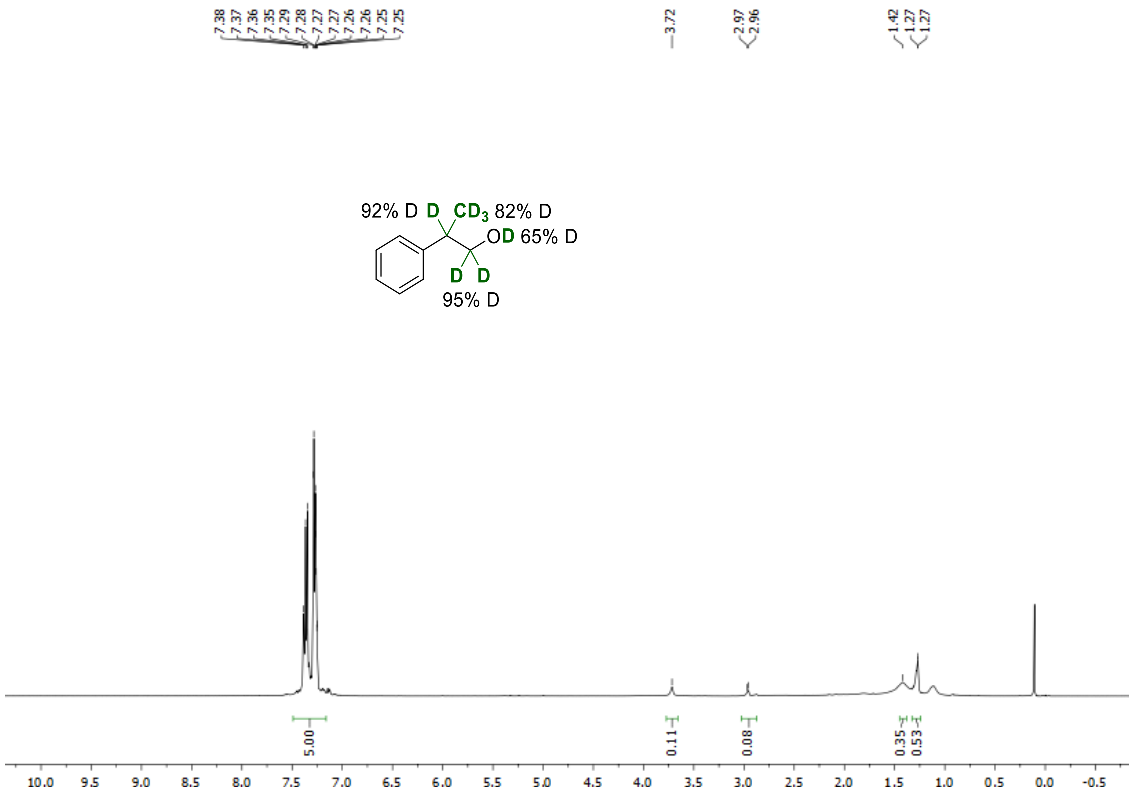

Figure S5: ^{1}H NMR (400 MHz, $CDCl_3$, 298 K) reaction mixture spectrum for the reaction of 2a with CO and D_2.

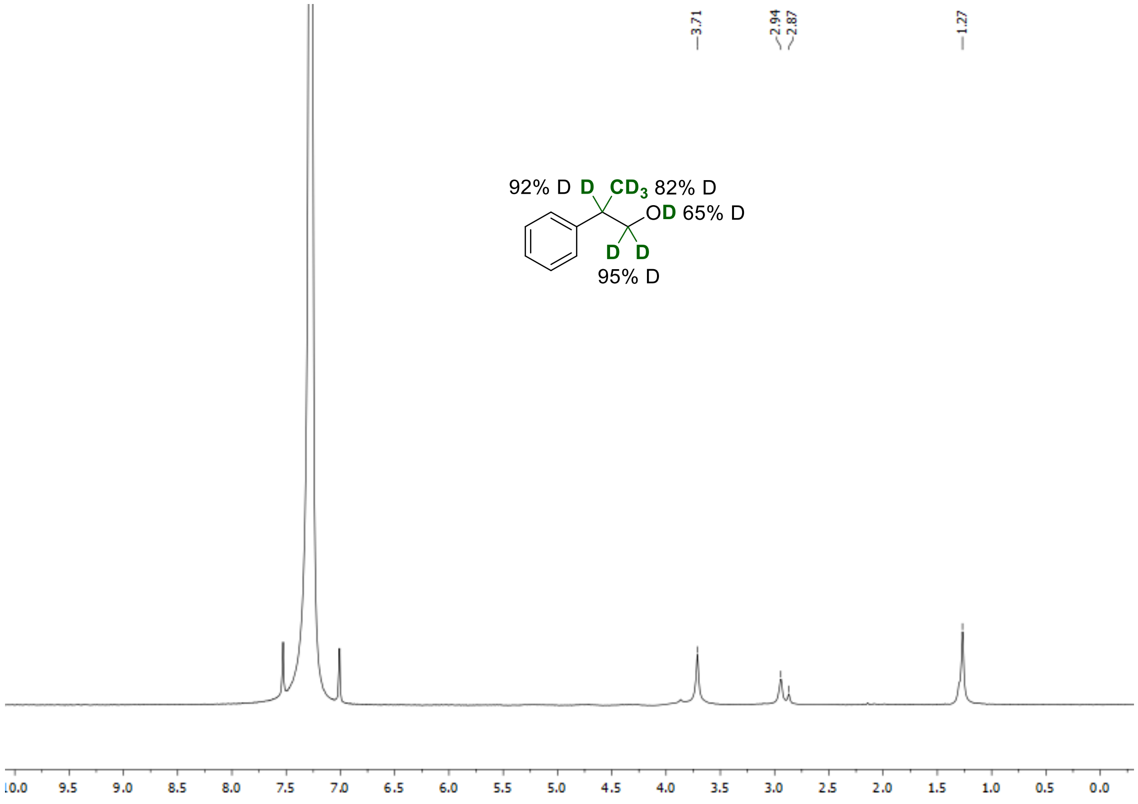

Figure S6: ^{2}H NMR (61 MHz, $CDCl_3$, 298 K) reaction mixture spectrum for the reaction of 2a with CO and D_2.

5.2. Reaction of 2-phenylethan-1-ol 2a with D_2 under standard reaction conditions

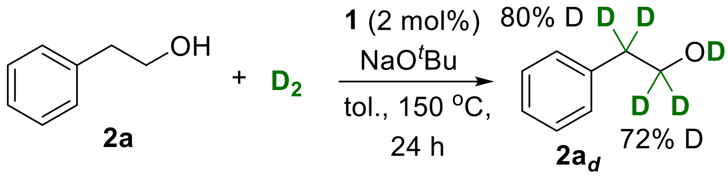

Mn-MACHO-^{i}Pr **1** (4.95 mg, 2 mol%) and NaO^{t}Bu (96.10 mg, 1 mmol) were measured into a glass inlet equipped with a stirring bar inside a glovebox. The glass inlet was closed with a septum and transferred into the bottom part of the 10 mL steel autoclave, where it was opened under a stream of argon. After sealing, the autoclave was evacuated and purged with argon three times. 2-phenylethan-1-ol **2a** (61.0 mg, 0.5 mmol) and toluene (0.8 mL) were added at room temperature through a valve under argon. The autoclave was sealed, pressurized with D_2 (10 bar) and heated to 150 °C temperature. After 24 h, the autoclave was cooled to room temperature and slowly vented while stirring continued. The reaction mixture was further analyzed by NMR spectroscopy.

^{1}H NMR (400 MHz, $CDCl_3$, 298 K) δ = 7.33-7.37 (m, 2H, ArC*H*), 7.24-7.28 (m, 3H, ArC*H*), 3.92-3.97 (m, 0.57H, CH_2), 2.94-3.00 (m, 0.40 H, CH_2).

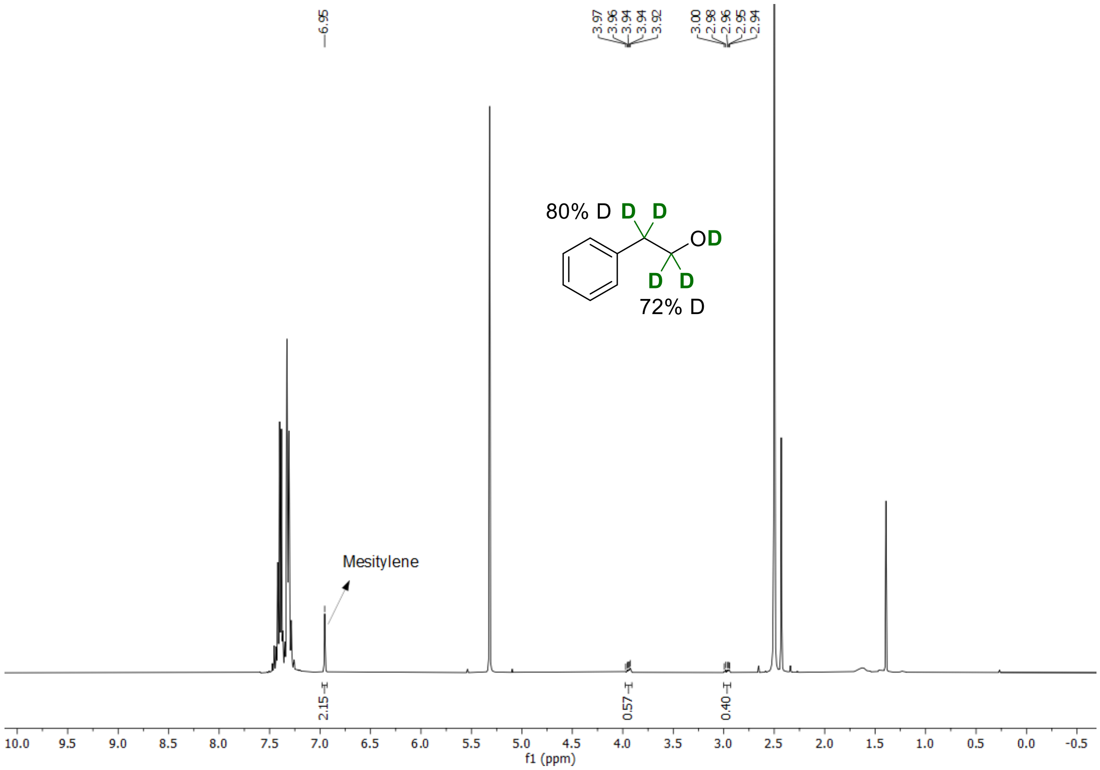

Figure S7: ^{1}H NMR (400 MHz, $CDCl_3$, 298 K) reaction mixture spectrum for the reaction of 2a with D_2.

5.3. Reaction of 2-phenylpropan-1-ol 3a with D_2 under standard reaction conditions

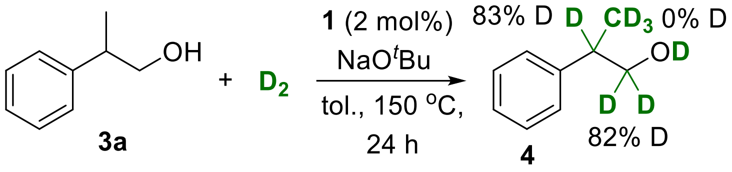

Mn-MACHO-^{i}Pr **1** (4.95 mg, 2 mol%) and NaO^{t}Bu (96.10 mg, 1 mmol) were measured into a glass inlet equipped with a stirring bar inside a glovebox. The glass inlet was closed with a septum and transferred into the bottom part of the 10 mL steel autoclave, where it was opened under a stream of argon. After sealing, the autoclave was evacuated and purged with argon three times. 2-phenylpropan-1-ol **3a** (68.1 mg, 0.5 mmol) and toluene (0.8 mL) were added at room temperature through a valve under argon. The autoclave was sealed, pressurized with D_2 (10 bar) and heated to 150 °C temperature. After 24 h, the autoclave was cooled to room temperature and slowly vented while stirring continued. The reaction mixture was further analyzed by NMR spectroscopy.

^{1}H NMR (400 MHz, $CDCl_3$, 298 K) δ = 7.34-7.38 (m, 2H, ArC*H*), 7.25-7.28 (m, 3H, ArC*H*), 3.70 (br s, 0.26H, C*H*$_2$), 2.94-2.99 (m, 017H, C*H*$_2$), 1.29 (s, 3H, C*H*$_3$).

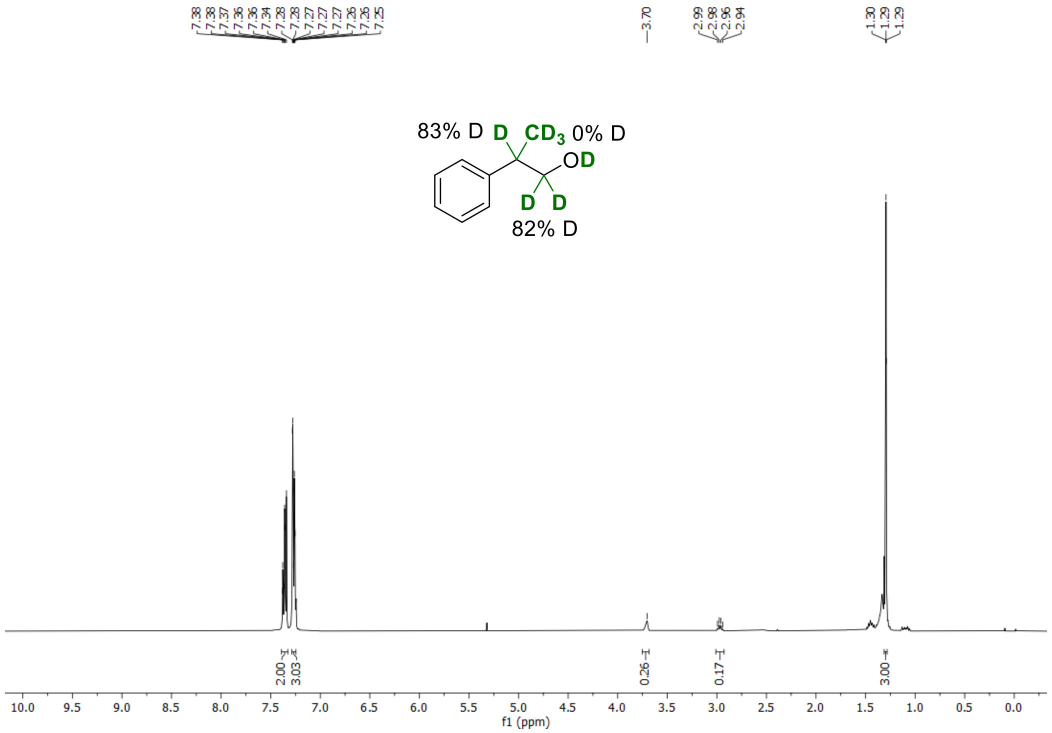

Figure S8: ^{1}H NMR (400 MHz, $CDCl_3$, 298 K) reaction mixture spectrum for the reaction of 3a with D_2.

5.4. Reaction of 2-phenylethan-1-ol 2a with carbon monoxide in the presence of a base

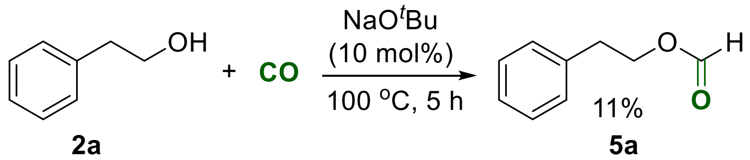

NaO^{t}Bu (9.6 mg, 0.1 mmol) were measured into a glass inlet equipped with a stirring bar inside a glovebox. The glass inlet was closed with a septum and transferred into the bottom part of the 10 mL steel autoclave, where it was opened under a stream of argon. After sealing, the autoclave was evacuated and purged with argon three times. 2-phenylethan-1-ol **2a** (122.2 mg, 1.0 mmol) and toluene (0.8 mL) were added at room temperature through a valve under argon. The autoclave was sealed, pressurized with CO (5 bar) and heated to 100 °C. After 5 h, the autoclave was cooled to room temperature and slowly vented while stirring continued. The reaction mixture was further analyzed by NMR spectroscopy. ($\delta_{(standard)}$ = 3.78 (t, 2H), $\delta_{product}$ = 4.33 (t, 2H)). The spectral data are in agreement with the reported literature.[4]

NMR Yield of **5a**: 11%.

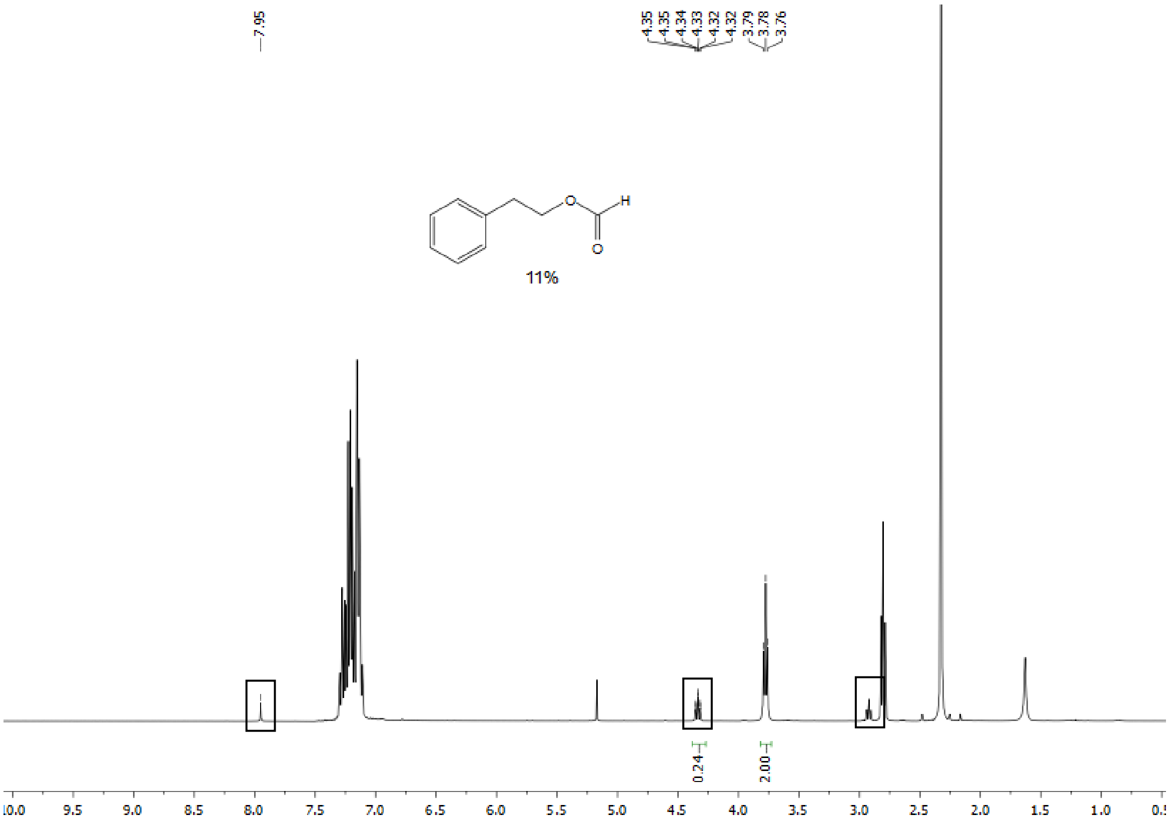

Figure S9: ^{1}H NMR (400 MHz, $CDCl_3$, 298 K) reaction mixture spectrum for the reaction of 2a with CO in the presence of a base.

5.5. Reaction of 2-Phenethyl formate 5a with CO and H_2 using 1

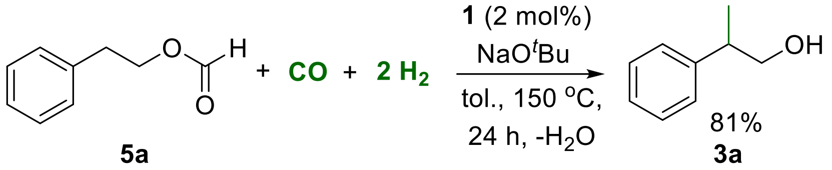

Mn-MACHO-^{i}Pr **1** (4.95 mg, 2 mol%) and NaO^{t}Bu (96.10 mg, 1 mmol) were measured into a glass inlet equipped with a stirring bar inside a glovebox. The glass inlet was closed with a septum and transferred into the bottom part of the 10 mL steel autoclave, where it was opened under a stream of argon. After sealing, the autoclave was evacuated and purged with argon three times. 2-Phenethyl formate **5a** (75.1 mg, 0.5 mmol) and toluene (0.8 mL) were added at room temperature through a valve under argon. The autoclave was sealed, pressurized with CO (5 bar) and H_2 (15 bar) and heated to 150 °C temperature. After 24 h, the autoclave was cooled to room temperature and slowly vented while stirring continued. Mesitylene (43.2 mg, 0.36 mmol) was added as an internal standard to the reaction mixture that was then passed through a short path of acidic alumina before the composition was analyzed by NMR spectroscopy. NMR yield of **3a**: 81%.

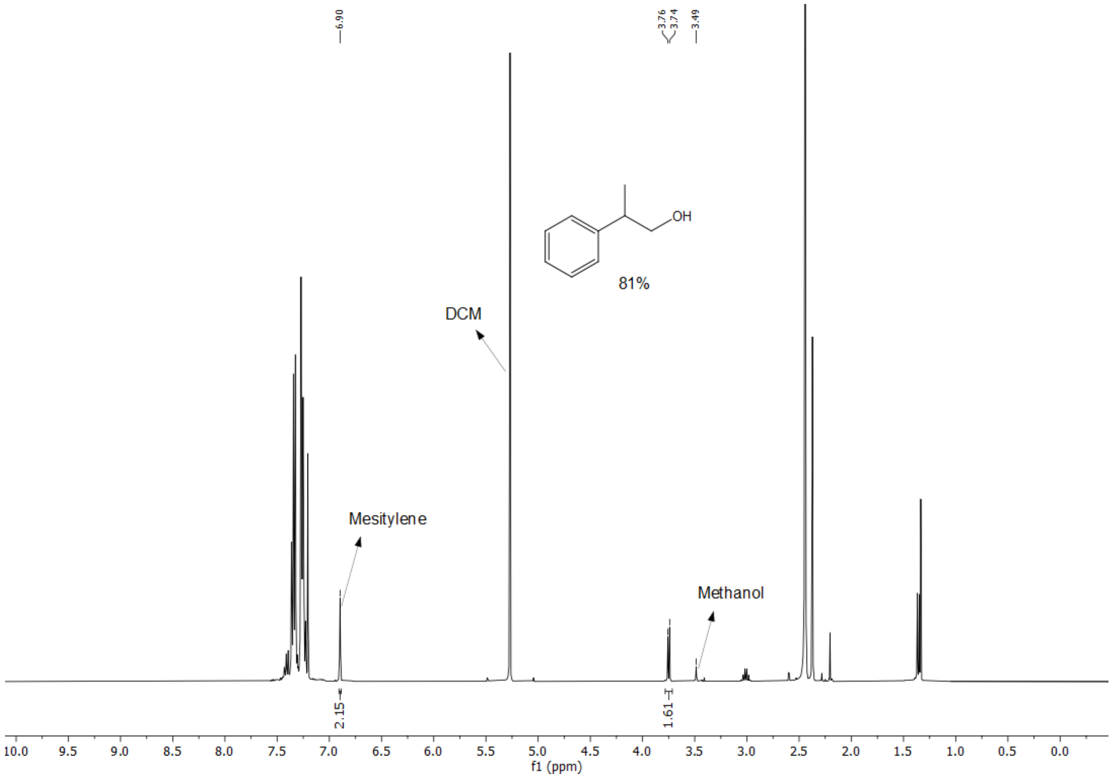

Figure S10: ^{1}H NMR (400 MHz, $CDCl_3$, 298 K) reaction mixture spectrum for the reaction of 5a with CO and H_2.

5.6. Reaction of butyl formate 5b with CO and H_2 using 1

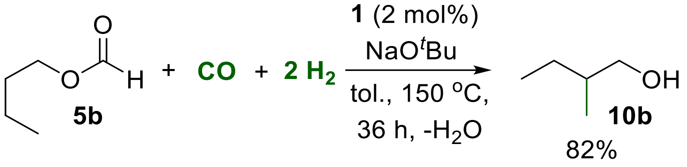

Mn-MACHO-^{i}Pr **1** (4.95 mg, 2 mol%) and NaO^{t}Bu (96.10 mg, 1 mmol) were measured into a glass inlet equipped with a stirring bar inside a glovebox. The glass inlet was closed with a septum and transferred into the bottom part of the 10 mL steel autoclave, where it was opened under a stream of argon. After sealing, the autoclave was evacuated and purged with argon three times. Butyl formate **5b** (51.1 mg, 0.5 mmol) and toluene (0.8 mL) were added at room temperature through a valve under argon. The autoclave was sealed, pressurized with CO (5 bar) and H_2 (15 bar) and heated to 150 °C temperature. After 36 h, the autoclave was cooled to room temperature and slowly vented while stirring continued. Mesitylene (43.2 mg, 0.36 mmol) was added as an internal standard to the reaction mixture that was then passed through a short path of acidic alumina before the composition was analyzed by NMR spectroscopy. NMR yield of **10b**: 82%.

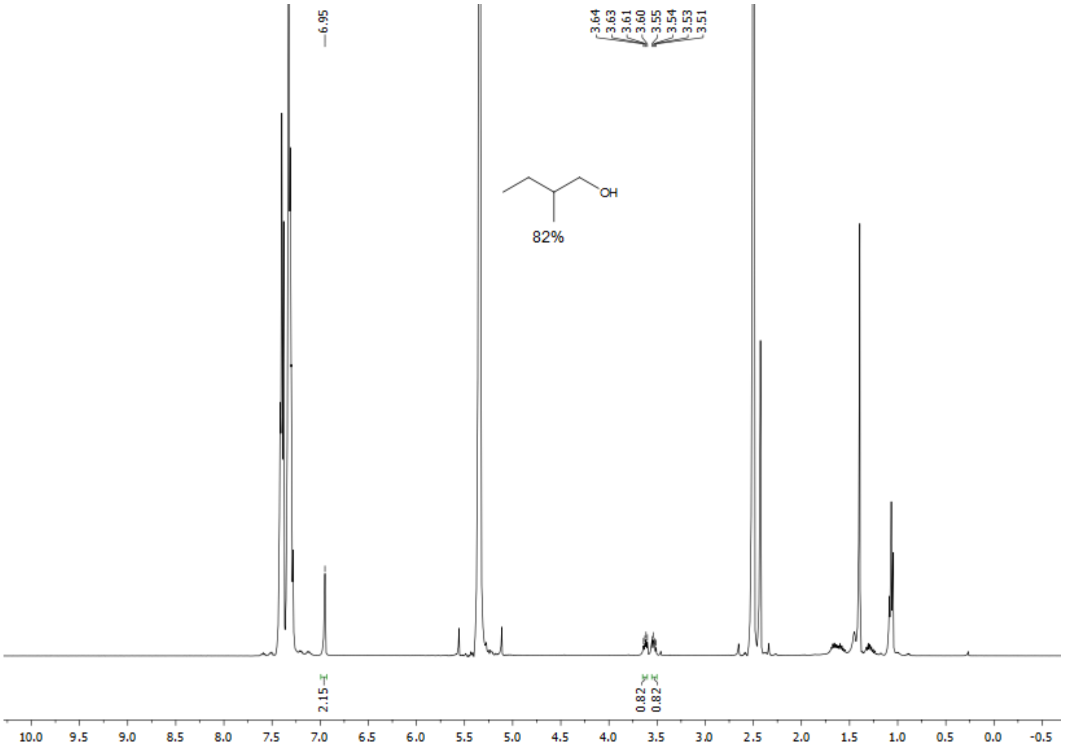

Figure S11: ^{1}H NMR (400 MHz, $CDCl_3$, 298 K) reaction mixture spectrum for the reaction of 5b with CO and H_2.

5.7. Reaction of 2a with ethyl formate 6 and hydrogen

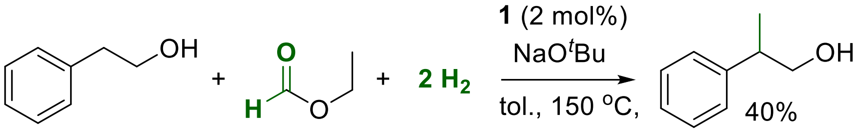

Mn-MACHO-^{i}Pr **1** (4.95 mg, 2 mol%) and NaO^{t}Bu (96.10 mg, 1 mmol) were measured into a glass inlet equipped with a stirring bar inside a glovebox. The glass inlet was closed with a septum and transferred into the bottom part of the 10 mL steel autoclave, where it was opened under a stream of argon. After sealing, the autoclave was evacuated and purged with argon three times. 2-phenylethan-1-ol **2a** (61.1 mg, 0.5 mmol), ethyl formate **6** (222.2 mg, 3 mmol) and toluene (0.8 mL) were added at room temperature through a valve under argon. The autoclave was sealed, pressurized with H_2 (15 bar) and heated to 150 °C temperature. After 24 h, the autoclave was cooled to room temperature and slowly vented while stirring continued. Mesitylene (43.2 mg, 0.36 mmol) was added as an internal standard to the reaction mixture that was then passed through a short path of acidic alumina before the composition was analyzed by NMR spectroscopy.

NMR yield of **3a**: 40%.

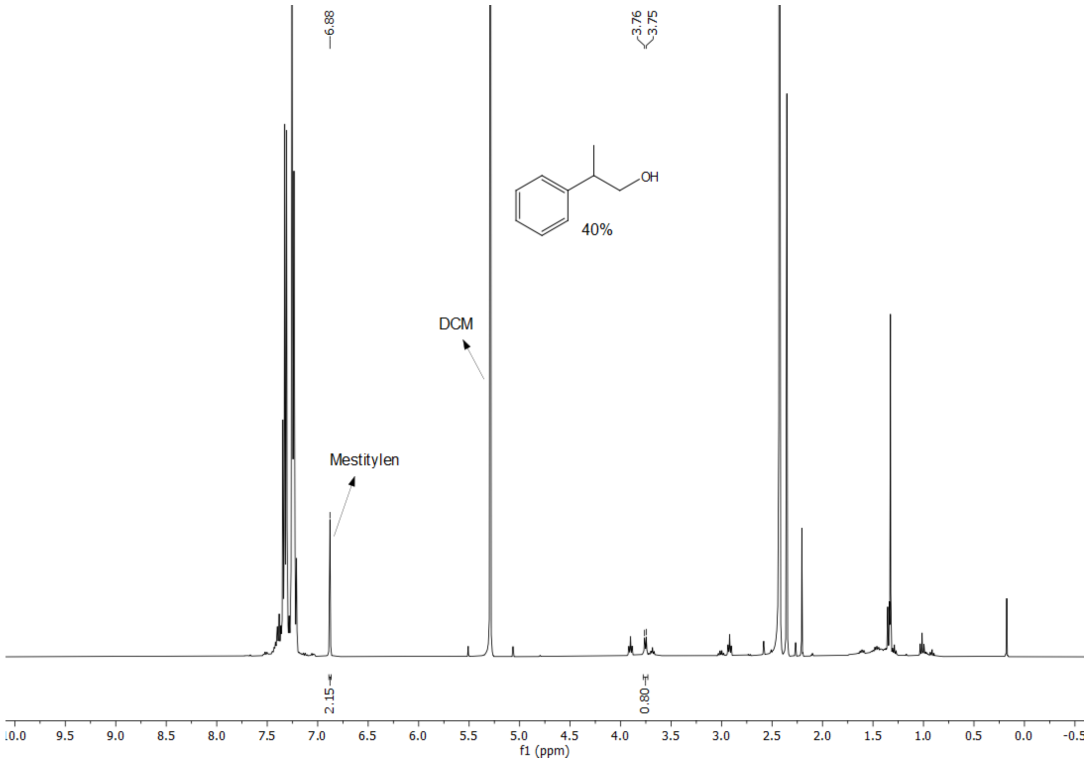

Figure S12: ^{1}H NMR (400 MHz, $CDCl_3$, 298 K) reaction mixture spectrum for the reaction of 2a with 6 and H_2.

5.8. Reaction of CO with H_2 using complex 1

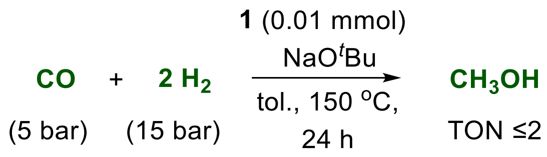

Mn-MACHO-^{i}Pr **1** (4.95 mg) and NaO^{t}Bu (96.10 mg, 1 mmol) were measured into a glass inlet equipped with a stirring bar inside a glovebox. The glass inlet was closed with a septum and transferred into the bottom part of the 10 mL steel autoclave, where it was opened under a stream of argon. After sealing, the autoclave was evacuated and purged with argon three times. Toluene (0.8 mL) was added at room temperature through a valve under argon. The autoclave was sealed, pressurized with CO (5 bar) and H_2 (15 bar) and heated to 150 °C temperature. After 24 h, the autoclave was cooled to room temperature and slowly vented while stirring continued. Mesitylene (43.2 mg, 0.36 mmol) was added as an internal standard to the reaction mixture that was then passed through a short path of acidic alumina before the composition was analyzed by NMR spectroscopy.

TON of MeOH: ≤2

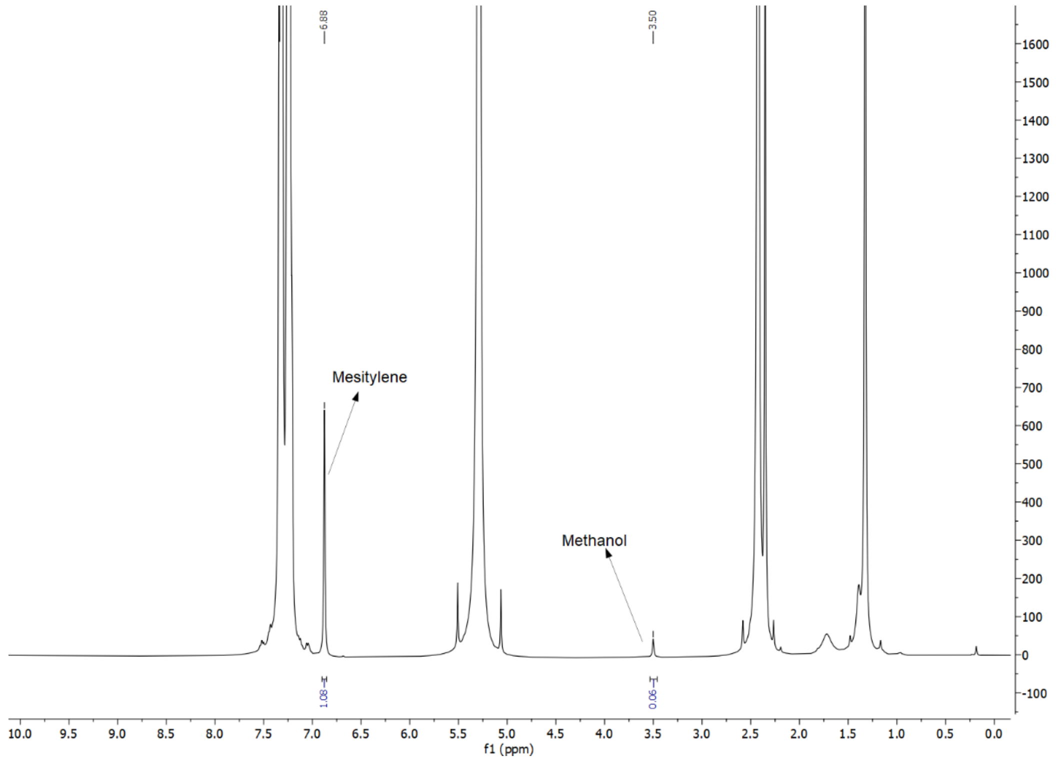

Figure S13: ^{1}H NMR (400 MHz, $CDCl_3$, 298 K) reaction mixture spectrum for the hydrogenation of CO using 1.

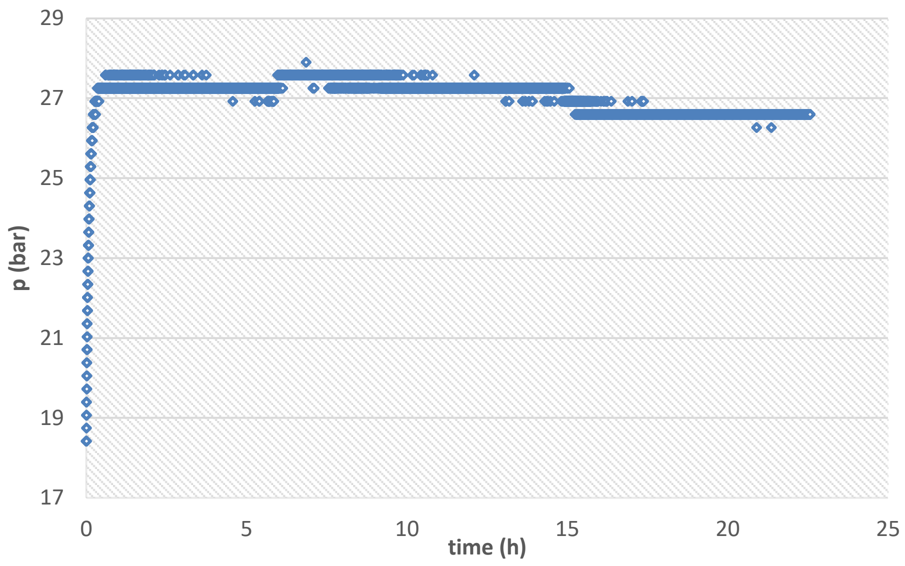

Figure S14: Pressure curve for the hydrogenation of carbon monoxide using complex 1.

6. Standard procedure for the catalytic β-methylation of aryl substituted alcohols

Mn-MACHO-^{i}Pr **1** (4.95 mg, 2 mol%) and NaO^{t}Bu (96.1 mg, 1 mmol) were measured into a glass inlet equipped with a stirring bar inside a glovebox. The glass inlet was closed with a septum and transferred into the bottom part of the 10 mL steel autoclave, where it was opened under a stream of argon. After sealing, the autoclave was purged with argon three times. Alcohol (0.5 mmol) and toluene (0.8 mL) were added at room temperature through a valve under argon. The autoclave was sealed, pressurized with CO (5 bar) and H_2 (15 bar) and heated to 150 °C temperature for 24 h. After completion of the reaction, the autoclave was cooled to room temperature and slowly vented while stirring continued. Mesitylene was added as an internal standard to the reaction mixture that was then passed through a short path of acidic alumina before the composition was analyzed by NMR spectroscopy. The isolation of pure product was carried out via column chromatography over silica gel (100-200 mesh) using ethyl acetate/pentane (10 : 90) mixture as eluent.

2-phenylpropan-1-ol 3a: Prepared by following the general experimental procedure with: **1** (4.95 mg, 2.0 mol%), 2-phenylethan-1-ol **2a** (61.0 mg, 0.5 mmol), NaO^{t}Bu (96.1 mg, 1 mmol), toluene (0.8 mL), CO (5 bar), H_2 (15 bar). Yield was determined by ^{1}H NMR spectrum using mesitylene (43.2 mg, 0.36 mmol) as an internal standard ($\delta_{\text{Mesitylene(standard)}}$ = 7.00 (s, 3H), δ_{product} = 3.85 (d, 2H)).The spectral data are in agreement with the reported literature.[5]

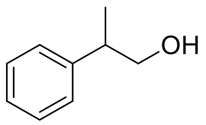

^{1}H NMR (400 MHz, $CDCl_3$, 298 K) δ = 7.24-7.28 (m, 2H, ArC*H*), 7.14-7.18 (m, 3H, ArC*H*), 3.62 (d, 2H, *J* = 6.83 Hz, CH_2), 2.83-2.92 (m, 1H, C*H*), 1.41 (br s, 1H, O*H*), 1.20 (d, 3H, *J* = 7.02 Hz, CH_3).

^{13}C{^{1}H}-NMR (101 MHz, $CDCl_3$, 298 K) δ = 143.79 (C_{Ar}), 128.77 (CH_{Ar}), 127.62 (CH_{Ar}), 126.81 (CH_{Ar}), 68.84 (CH_2), 42.57 (*C*H), 17.71 (CH_3).

Isolated yield: 86%.

2-(*p*-tolyl)propan-1-ol 3b: Prepared by following the general experimental procedure with: **1** (4.95 mg, 2.0 mol%), 2-(*p*-tolyl)ethanol **2b** (68.0 mg, 0.5 mmol), NaO^{t}Bu (96.1 mg, 1 mmol), toluene (0.8 mL), CO (5 bar), H_2 (15 bar). Yield was determined by ^{1}H NMR spectrum using mesitylene (43.2 mg, 0.36 mmol) as an internal standard ($\delta_{\text{Mesitylene(standard)}}$ = 7.07 (s, 3H), δ_{product} = 3.89 (d, 2H)). The spectral data are in agreement with the reported literature.[5]

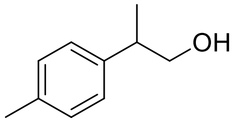

^{1}H NMR (400 MHz, $CDCl_3$, 298 K) δ = 7.12-7.17 (m, 4H, ArCH), 3.69 (d, 2H, *J* = 6.94 Hz, CH_2), 2.88-2.97 (m, 1H, C*H*), 2.34 (s, 3H, CH_3), 1.33 (br s, 1H, O*H*), 1.26 (d, 3H, *J* = 7.00 Hz, CH_3).

$^{13}C\{^1H\}$**-NMR** (101 MHz, $CDCl_3$, 298 K) δ = 140.67 (*C*$_{Ar}$), 136.38 (*C*$_{Ar}$), 129.49 (*C*H$_{Ar}$), 127.49 (*C*H$_{Ar}$), 68.91 (*C*H$_2$), 42.16 (*C*H), 21.14 (*C*H$_3$), 17.80 (*C*H$_3$).

Isolated yield: 90%.

2-(4-isobutylphenyl)propan-1-ol 3c: Prepared by following the general experimental procedure with: **1** (4.95 mg, 2.0 mol%), 2-(4-isobutylphenyl)ethanol **2c** (89.0 mg, 0.5 mmol), NaO^{t}Bu (96.1 mg, 1 mmol), toluene (0.8 mL), CO (5 bar), H_2 (15 bar). Yield was determined by ^{1}H NMR spectrum using mesitylene (43.2 mg, 0.36 mmol) as an internal standard ($δ_{Mesitylene(standard)}$ = 7.04 (s, 3H), $δ_{product}$ = 3.87 (d, 2H)). The spectral data are in agreement with the reported literature.[6]

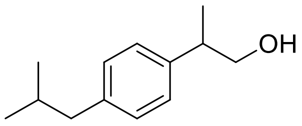

^{1}**H NMR** (400 MHz, $CDCl_3$, 298 K) δ = 7.10-7.16 (m, 4H, ArC*H*), 3.69 (d, 2H, *J* = 6.82 Hz, C*H*$_2$), 2.88-2.97 (m, 1H, C*H*), 2.45 (d, 2H, *J* = 7.16 Hz, C*H*$_2$), 1.80-1.91 (m, 1H, C*H*), 1.38 (br s, 1H, O*H*), 1.27 (d, 3H, *J* = 7.01 Hz, C*H*$_2$), 0.91 (d, 6H, *J* = 6.61 Hz, CH_3).

$^{13}C\{^1H\}$**-NMR** (101 MHz, $CDCl_3$, 298 K) δ = 140.84 (*C*$_{Ar}$), 140.20 (*C*$_{Ar}$), 129.51 (*C*H$_{Ar}$), 127.29 (*C*H$_{Ar}$), 68.92 (*C*H$_2$), 45.17 (*C*H$_2$), 42.16 (*C*H), 30.35 (*C*H), 22.54 (*C*H$_3$), 17.74 (*C*H$_3$).

Isolated yield: 85%.

2-(4-methoxyphenyl)propan-1-ol 3d: Prepared by following the general experimental procedure with: **1** (4.95 mg, 2.0 mol%), 2-(4-methoxyphenyl)ethan-1-ol **2d** (76.1 mg, 0.5 mmol), NaO^{t}Bu (96.1 mg, 1 mmol), toluene (0.8 mL), CO (5 bar), H_2 (15 bar). Yield was determined by ^{1}H NMR spectrum using mesitylene (43.2 mg, 0.36 mmol) as an internal standard ($δ_{Mesitylene(standard)}$ = 6.70 (s, 3H), $δ_{product}$ = 3.52-3.54 (m, 2H)). The spectral data are in agreement with the reported literature.[5]

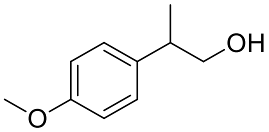

^{1}**H NMR** (400 MHz, $CDCl_3$, 298 K) δ = 7.16 (d, 2H, *J* = 8.60 Hz, ArC*H*), 6.88 (d, 2H, *J* = 8.65 Hz, ArC*H*), 3.80 (s, 3H, OC*H*$_3$), 3.65-3.67 (m, 2H, C*H*$_2$), 2.86-2.94 (m, 1H, C*H*), 1.53 (br s, 1H, O*H*), 1.25 (d, 3H, *J* = 6.99 Hz, C*H*$_3$).

$^{13}C\{^1H\}$**-NMR** (101 MHz, $CDCl_3$, 298 K) δ = 158.42 (*C*$_{Ar}$), 135.72 (*C*$_{Ar}$), 128.48 (*C*H$_{Ar}$), 114.14 (*C*H$_{Ar}$), 68.91 (*C*H$_2$), 55.37 (O*C*H$_3$), 41.68 (*C*H), 17.85 (*C*H$_3$).

Isolated yield: 84%.

2-(3-methoxyphenyl)propan-1-ol 3e: Prepared by following the general experimental procedure with: **1** (4.95 mg, 2.0 mol%), 2-(3-methoxyphenyl)ethan-1-ol **2e** (76.1 mg, 0.5 mmol), NaO^{t}Bu (96.1 mg, 1 mmol), toluene (0.8 mL), CO (5 bar), H_2 (15 bar). Yield was determined by ^{1}H NMR spectrum using mesitylene (43.2 mg, 0.36 mmol) as an

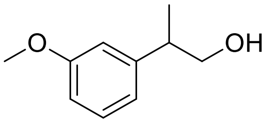

internal standard ($\delta_{Mesitylene(standard)}$ = 6.70 (s, 3H), $\delta_{product}$ = 3.62 (d, 2H)). The spectral data are in agreement with the reported literature.[6]

^{1}H NMR (400 MHz, $CDCl_3$, 298 K) δ = 7.18 (t, 1H, *J* = 8.12 Hz, ArC*H*), 6.76 (d, 1H, *J* = 7.54 Hz, ArC*H*), 6.70-6.72 (m, 2H, ArC*H*), 3.74 (s, 3H, OC*H*$_3$), 3.63 (d, 2H, *J* = 6.27 Hz, C*H*$_2$), 2.83-2.88 (m, 1H, C*H*), 1.52 (br s, 1H, O*H*), 1.19 (d, 3H, *J* = 6.63 Hz, CH_3).

^{13}C{^{1}H}-NMR (101 MHz, $CDCl_3$, 298 K) δ = 159.92 (C_{Ar}), 145.47 (C_{Ar}), 129.74 (CH_{Ar}), 119.90 (CH_{Ar}), 113.58 (CH_{Ar}), 111.81 (CH_{Ar}), 68.79 (CH_2), 55.28 (OCH_3), 42.64 (*C*H), 17.68 (CH_3).

Isolated yield: 66%.

2-(6-methoxynaphthalen-2-yl)propan-1-ol 3f: Prepared by following the general experimental procedure

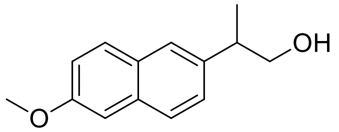

with: **1** (4.95 mg, 2.0 mol%), 2-(6-methoxynaphthalen-2-yl)ethanol **2f** (101.2 mg, 0.5 mmol), NaO^{t}Bu (96.1 mg, 1 mmol), toluene (0.8 mL), CO (5 bar), H_2 (15 bar). Yield was determined by ^{1}H NMR spectrum using mesitylene (43.2 mg, 0.36 mmol) as an internal standard ($\delta_{Mesitylene(standard)}$ = 6.70 (s, 3H), $\delta_{product}$ = 1.14 (d, 3H)). The spectral data are in agreement with the reported literature.[7]

^{1}H NMR (400 MHz, CD_3OD, 298 K) δ = 7.56-7.60 (dd, 2H, *J* = 5.49, 8.63 Hz, ArC*H*), 7.48 (s, 1H, ArC*H*), 7.22-7.24 (dd, 1H, *J* = 1.71, 8.49 Hz, ArC*H*), 7.06 (d, 1H, *J* = 2.44 Hz, ArC*H*), 6.96-6.99 (dd, 1H, *J* = 2.53, 8.95 Hz, ArC*H*), 3.77 (s, 3H, C*H*$_3$), 3.51-3.66 (dd, 2H, *J* = 6.94, 10.72, 42.79 Hz, C*H*$_2$), 3.21 (br. s, 1H, O*H*), 2.83-2.94 (m, 1H, C*H*), 1.23 (d, 3H, *J* = 6.98 Hz, C*H*$_3$).

^{13}C{^{1}H}-NMR (101 MHz, CD_3OD, 298 K) δ = 158.71 (C_{Ar}), 140.84 (C_{Ar}), 134.92 (C_{Ar}), 130.54 (C_{Ar}), 130.02 (CH_{Ar}), 127.88 (CH_{Ar}), 127.49 (CH_{Ar}), 126.51 (CH_{Ar}), 119.55 (CH_{Ar}), 106.50 (CH_{Ar}), 69.05 (CH_2), 55.65 (CH_3), 43.61 (*C*H), 18.39 (CH_3).

Isolated yield: 86%.

2-(4-chlorophenyl)propan-1-ol 3g: Prepared by following the general experimental procedure with: **1**

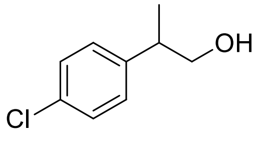

(4.95 mg, 2.0 mol%), 2-(4-chlorophenyl)ethan-1-ol **2g** (79.3 mg, 0.5 mmol), NaO^{t}Bu (96.1 mg, 1 mmol), toluene (0.8 mL), CO (5 bar), H_2 (15 bar). Yield was determined by ^{1}H NMR spectrum using mesitylene (43.2 mg, 0.36 mmol) as an internal standard ($\delta_{Mesitylene(standard)}$ = 7.03 (s, 3H), $\delta_{product}$ = 3.82 (d, 2H)). The spectral data are in agreement with the reported literature.[8]

^{1}H NMR (400 MHz, $CDCl_3$, 298 K) δ = 7.22 (d, 2H, *J* = 8.40 Hz, ArC*H*), 7.10 (d, 2H, 8.43 Hz, ArC*H*), 3.58-3.61 (m, 2H, C*H*$_2$), 2.80-2.89 (m, 1H, C*H*), 1.42 (br, 1H, O*H*), 1.18 (d, 3H, *J* = 7.04 Hz, C*H*$_3$).

$^{13}C\{^1H\}$**-NMR** (101 MHz, $CDCl_3$, 298 K) δ = 142.34 (C_{Ar}), 132.43 (C_{Ar}), 128.94 (CH_{Ar}), 128.82 (CH_{Ar}), 68.60 (CH_2), 41.97 (CH), 17.66 (CH_3).

Isolated yield: 74%.

2-(4-fluorophenyl)propan-1-ol 3h: Prepared by following the general experimental procedure with: **1** (4.95 mg, 2.0 mol%), 2-(4-fluorophenyl)ethan-1-ol **2h** (70.0 mg, 0.5 mmol), NaO^{t}Bu (96.1 mg, 1 mmol), toluene (0.8 mL), CO (5 bar), H_2 (15 bar). Yield was determined by ^{1}H NMR spectrum using mesitylene (43.2 mg, 0.36 mmol) as an internal standard ($\delta_{Mesitylene(standard)}$ = 6.70 (s, 3H), $\delta_{product}$ = 1.14 (d, 3H)). The spectral data are in agreement with the reported literature.[9]

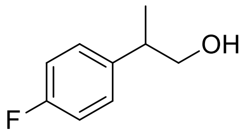

^{1}**H NMR** (400 MHz, $CDCl_3$, 298 K) δ = 7.18-7.22 (m, 2H, ArCH), 6.99-7.04 (m, 2H, ArCH), 3.66-3.69 (dd, 2H, J = 3.18, 6.80 Hz, CH_2), 2.90-2.98 (m, 1H, CH), 1.45 (br s, 1H, OH), 1.26 (d, 3H, J = 7.03 Hz, CH_3).

$^{13}C\{^1H\}$**-NMR** (101 MHz, $CDCl_3$, 298 K) δ = 161.79 (d, C_{Ar}, J = 244.40 Hz), 139.46 (d, C_{Ar}, J = 3.20 Hz), 128.98 (d, CH_{Ar}, J = 7.79 Hz), 115.51 (d, CH_{Ar}, J = 21.05), 68.80 (CH_2), 41.84 (CH), 17.85 (CH_3).

Isolated yield: 71%.

2-(thiophen-2-yl)propan-1-ol 3i: Prepared by following the general experimental procedure with: **1** (4.95 mg, 2.0 mol%), 2-(thiophen-2-yl)ethan-1-ol **2i** (64.1 mg, 0.5 mmol), NaO^{t}Bu (96.1 mg, 1 mmol), toluene (0.8 mL), CO (5 bar), H_2 (15 bar). Yield was determined by ^{1}H NMR spectrum using mesitylene (43.2 mg, 0.36 mmol) as an internal standard ($\delta_{Mesitylene(standard)}$ = 7.06 (s, 3H), $\delta_{product}$ = 3.64-3.74 (m, 2H)). The spectral data are in agreement with the reported literature.[10]

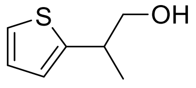

^{1}**H NMR** (400 MHz, $CDCl_3$, 298 K) δ = 7.19-7.20 (dd, 1H, J = 1.02, 5.09 Hz, ArCH), 6.96-6.99 (dd, 1H, J = 3.45, 5.08 Hz, ArCH), 6.90 (d, 1H, J = 3.17 Hz, ArCH), 3.64-3.76 (m, 2H, CH_2), 3.21-3.29 (m, 1H, CH), 1.53 (br s, 1H, OH), 1.36 (d, 3H, J = 6.95 Hz, CH_3).

$^{13}C\{^1H\}$**-NMR** (101 MHz, $CDCl_3$, 298 K) δ = 147.35 (C_{Ar}), 126.84 (CH_{Ar}), 123.92 (CH_{Ar}), 123.56 (CH_{Ar}), 69.01 (CH_2), 38.15(CH), 18.61 (CH_3).

Isolated yield: 86%.

2-methyl-3-phenylpropan-1-ol 3j: Prepared by following the general experimental procedure with: **1** (4.95 mg, 2.0 mol%), 3-phenylpropan-1-ol **2j** (68.0 mg, 0.5 mmol), NaO^{t}Bu (96.1 mg, 1 mmol), toluene (0.8 mL), CO (5 bar), H_2 (15 bar). Yield was determined by ^{1}H NMR spectrum using mesitylene (43.2 mg, 0.36 mmol) as an internal standard

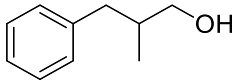

($\delta_{Mesitylene(standard)}$ = 7.06 (s, 3H), $\delta_{product}$ = 3.64-3.74 (ddd, 2H)). The spectral data are in agreement with the reported literature.[11]

^{1}H NMR (400 MHz, $CDCl_3$, 298 K) δ = 7.30-7.35 (m, 2H, ArC*H*), 7.21-7.24 (m, 3H, ArC*H*), 3.50-3.61 (ddd, 2H, *J* = 5.95, 10.56, 24.80 Hz, C*H*$_2$), 2.78-2.83 (dd, 1H, C*H*), 2.45-2.50 (dd, 1H, C*H*), 1.95-2.02 (m, 1H, CH), 1.03 (br s, 1H, O*H*), 0.97 (d, 3H, *J* = 6.73 Hz, C*H*$_3$).

^{13}C{^{1}H}-NMR (101 MHz, $CDCl_3$, 298 K) δ = 140.75 (*C*$_{Ar}$), 129.27 (*C*H$_{Ar}$), 128.39 (*C*H$_{Ar}$), 126.00 (*C*H$_{Ar}$), 67.80 (*C*H$_2$), 39.84 (*C*H$_2$), 37.92 (*C*H), 16.60 (*C*H$_3$).

Isolated yield: 75%.

3-(4-chlorophenyl)-2-methylpropan-1-ol 3k: Prepared by following the general experimental procedure

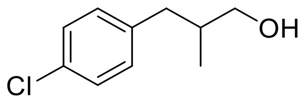

with: **1** (4.95 mg, 2.0 mol%), 3-(4-chlorophenyl)propan-1-ol **2k** (85.3 mg, 0.5 mmol), NaO^{t}Bu (96.1 mg, 1 mmol), toluene (0.8 mL), CO (5 bar), H_2 (15 bar). Yield was determined by ^{1}H NMR spectrum using mesitylene (43.2 mg, 0.36 mmol) as an internal standard ($\delta_{Mesitylene(standard)}$ = 7.02 (s, 3H), $\delta_{product}$ = 1.09 (d, 3H)). The spectral data are in agreement with the reported literature.[12]

^{1}H NMR (400 MHz, $CDCl_3$, 298 K) δ =7.17 (d, 2H, *J*=8.28 Hz, ArC*H*), 7.03 (d, 2H, *J* = 8.39 Hz, ArC*H*), 3.41-3.43 (m, 2H, C*H*$_2$), 2.67 (dd, 1H, *J* = 13.49, 6.08 Hz, C*H*), 2.31 (dd, 1H, *J* = 2.31 Hz, C*H*), 1.79-1.88 (m, 1H, C*H*), 1.44 (br. s, 1H, O*H*), 0.82 (d, 3H, *J* = 6.68 Hz, CH$_3$).

^{13}C{^{1}H}-NMR (101 MHz, $CDCl_3$, 298 K) δ = 139.19 (*C*$_{Ar}$), 131.74 (*C*$_{Ar}$), 130.61 (*C*H$_{Ar}$), 128.48 (*C*H$_{Ar}$), 67.54 (*C*H$_2$), 39.04 (*C*H$_2$), 37.81 (*C*H), 16.42 (*C*H$_3$).

Isolated yield: 86%.

2-methyl-4-phenylbutan-1-ol 3l: Prepared by following the general experimental procedure with: **1**

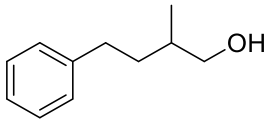

(4.95 mg, 2.0 mol%), 4-phenylbutan-1-ol **2l** (75.1 mg, 0.5 mmol), NaO^{t}Bu (96.1 mg, 1 mmol), toluene (0.8 mL), CO (5 bar), H_2 (15 bar). Yield was determined by ^{1}H NMR spectrum using mesitylene (43.2 mg, 0.36 mmol) as an internal standard ($\delta_{Mesitylene(standard)}$ = 7.04 (s, 3H), $\delta_{product}$ = 3.62-3.73 (ddd, 2H)). The spectral data are in agreement with the reported literature.[13]

^{1}H NMR (400 MHz, $CDCl_3$, 298 K) δ = 7.22-7.24 (m, 2H, ArC*H*), 7.14-7.16 (m, 3H, ArC*H*), 3.41-3.52 (ddd, 2H, *J* = 6.05, 10.38, 28.18 Hz, C*H*$_2$), 2.52-2.71 (dddd, 2H, *J* = 5.92, 10.04, 13.77, 29.92 Hz, C*H*$_2$), 1.67-1.77 (m, 1H, C*H*), 1.59-1.64 (m, 1H, C*H*), 1.40-1.43 (m, 2H, C*H* & O*H*), 0.95 (d, 3H, *J* = 6.60 Hz, C*H*$_3$).

^{13}C{^{1}H}-NMR (101 MHz, $CDCl_3$, 298 K) δ = 142.71 (*C*$_{Ar}$), 128.45 (*C*H$_{Ar}$), 125.83 (*C*H$_{Ar}$), 68.34 (*C*H$_2$), 35.50 (*C*H), 35.10 (*C*H$_2$), 33.41 (*C*H$_2$), 16.63 (*C*H$_3$).

Isolated yield: 80%.

2-methyl-5-phenylpentan-1-ol 3m: Prepared by following the general experimental procedure with: **1**

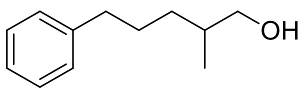

(4.95 mg, 2.0 mol%), 5-phenylpentan-1-ol **2m** (84.1 mg, 0.5 mmol), NaO^{t}Bu (96.1 mg, 1 mmol), toluene (0.8 mL), CO (5 bar), H_2 (15 bar). Yield was determined by ^{1}H NMR spectrum using mesitylene (43.2 mg, 0.36 mmol) as an internal standard ($\delta_{Mesitylene(standard)}$ = 7.05 (s, 3H), $\delta_{product}$ = 3.59-3.71 (ddd, 2H)). The spectral data are in agreement with the reported literature.[14]

^{1}H NMR (400 MHz, $CDCl_3$, 298 K) δ = 7.18-7.21 (m, 2H, ArC*H*), 7.10-7.12 (m, 3H, ArC*H*), 3.33-3.45 (ddd, 2H, *J* = 6.23, 10.37, 32.79 Hz, CH_2), 2.51-2.56 (m, 2H, CH_2), 1.51-1.63 (m, 4H, CH_2), 1.35-1.44 (m, 1H, C*H*), 1.10 (br s, 1H, O*H*), 0.85 (d, 3H, *J* = 6.58 Hz, CH_3).

^{13}C{^{1}H}-NMR (101 MHz, $CDCl_3$, 298 K) δ = 142.75 (C_{Ar}), 128.52 (CH_{Ar}), 128.42 (CH_{Ar}), 125.81 (CH_{Ar}), 68.42 (CH_2), 36.36 (CH_2), 35.82 (*C*H), 32.92 (CH_2), 29.06 (CH_2), 16.66 (CH_3).

Isolated yield: 67%.

2-methyl-8-phenyloctan-1-ol 3n: Prepared by following the general experimental procedure with: **1**

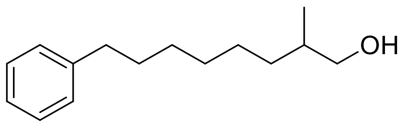

(4.95 mg, 2.0 mol%), 8-phenyloctan-1-ol **2n** (103.2 mg, 0.5 mmol), NaO^{t}Bu (96.1 mg, 1 mmol), toluene (0.8 mL), CO (5 bar), H_2 (15 bar). Yield was determined by ^{1}H NMR spectrum using mesitylene (43.2 mg, 0.36 mmol) as an internal standard ($\delta_{Mesitylene(standard)}$ = 7.07 (s, 3H), $\delta_{product}$ = 1.17 (d, 3H)). The spectral data are in agreement with the reported literature.[15]

^{1}H NMR (400 MHz, $CDCl_3$, 298 K) δ = 7.22-7.24 (m, 2H, ArC*H*), 7.14-7.15 (m, 3H, ArC*H*), 3.35-3.48 (ddd, 2H, *J* = 6.16, 10.45, 36.15 Hz, CH_2), 2.55-2.59 (m, 2H, CH_2), 1.54-1.58 (m, 3H, CH_2& C*H*), 1.29-1.30 (m, 9H, CH_2& O*H*), 0.87 (d, 3H, *J* = 6.71 Hz, CH_3).

^{13}C{^{1}H}-NMR (101 MHz, $CDCl_3$, 298 K) δ = 143.00 (C_{Ar}), 128.52 (CH_{Ar}), 128.35 (CH_{Ar}), 125.69 (CH_{Ar}), 68.53 (CH_2), 36.11 (CH_2), 35.88 (*C*H), 33.25 (CH_2), 31.63 (CH_2), 29.92 (CH_2), 29.42 (CH_2), 27.02 (CH_2), 16.71 (CH_3).

Isolated yield: 74%.

2-(methyl(phenyl)amino)propan-1-ol 3o: Prepared by following the general experimental procedure

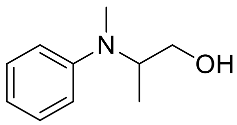

with: **1** (4.95 mg, 2.0 mol%), 2-(methyl(phenyl)amino)ethanol **2o** (75.6 mg, 0.5 mmol), NaO^{t}Bu (96.1 mg, 1 mmol), toluene (0.8 mL), CO (5 bar), H_2 (15 bar). Yield was determined by ^{1}H NMR spectrum using mesitylene (43.2 mg, 0.36 mmol)

as an internal standard ($\delta_{Mesitylene(standard)}$ = 2.52 (s, 9H), $\delta_{product}$ = 1.22 (d, 3H)). The spectral data are in agreement with the reported literature.[16]

^{1}H NMR (400 MHz, tol-d8, 298 K) δ = 7.13-7.17 (m, 2H, ArC*H*), 6.74-6.78 (m, 1H, ArC*H*), 6.69-6.73 (m, 2H, ArC*H*), 3.64-3.72 (m, 1H, C*H*), 3.20-3.30 (m, 2H, CH_2), 2.28 (s, 3H, CH_3), 1.54 (br. s, 1H, O*H*), 0.64 (d, 3H, *J* = 6.69 Hz, CH_3).

^{13}C{^{1}H}-NMR (101 MHz, tol-d8, 298 K) δ = 151.23 (C_{Ar}), 129.30 (CH_{Ar}), 118.43 (CH_{Ar}), 115.38 (CH_{Ar}), 63.74 (*C*H), 57.15 (CH_2), 29.72 (CH_3), 12.15 (CH_3).

Isolated yield: 66%.

7. Standard procedure for catalytic β-monomethylation of secondary alcohols

Mn-MACHO-^{i}Pr **1** (4.95 mg, 2 mol%) and NaO^{t}Bu (96.1 mg, 1 mmol) were measured into a glass inlet equipped with a stirring bar inside a glovebox. The glass inlet was closed with a septum and transferred into the bottom part of the 10 mL steel autoclave, where it was opened under a stream of argon. After sealing, the autoclave was evacuated and purged with argon three times. Secondary alcohol (0.5 mmol) and toluene (0.8 mL) were added at room temperature through a valve under argon. The autoclave was sealed, pressurized with CO (5 bar) and H_2 (15 bar) and heated to 150 ^{o}C temperature. After the certain reaction time, the autoclave was cooled to room temperature and slowly vented while stirring continued. Mesitylene was added as an internal standard to the reaction mixture that was then passed through a short path of acidic alumina before the composition was analyzed by NMR spectroscopy. The isolation of pure product was carried out via column chromatography over silica gel (100-200 mesh) using ethyl acetate/pentane (10 : 90) mixture as eluent.

2-methyl-1-phenylpropan-1-ol 8a: Prepared by following the general experimental procedure with:

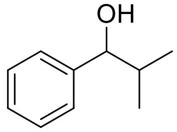

1 (4.95 mg, 2.0 mol%), 1-phenylpropan-1-ol **7a** (68.1 mg, 0.5 mmol), NaO^{t}Bu (96.1 mg, 1 mmol), toluene (0.8 mL), CO (5 bar), H_2 (15 bar) and reaction time: 24 h. Yield was determined by ^{1}H NMR spectrum using mesitylene (43.2 mg, 0.36 mmol) as an internal standard ($\delta_{Mesitylene(standard)}$ = 7.05 (s, 3H), $\delta_{product}$ = 4.54 (d, 1H)). The spectral data are in agreement with the reported literature.[5]

^{1}H NMR (400 MHz, $CDCl_3$, 298 K) δ = 7.20-7.29 (m, 5H, ArC*H*), 4.29 (d, 1H, *J* = 6.87 Hz, C*H*), 1.85-1.93 (m, 1H, C*H*), 1.78 (br s, 1H, O*H*), 0.93 (d, 3H, *J* = 6.67 Hz, CH_3), 0.73 (d, 3H, *J* =6.81 Hz, CH_3).

^{13}C{^{1}H}-NMR (101 MHz, CDCl3, 298 K) δ =143.78 (C_{Ar}), 128.32 (CH_{Ar}), 127.55 (CH_{Ar}), 126.70 (CH_{Ar}), 80.19 (*C*H), 35.40 (*C*H), 19.14 (CH_3), 18.37 (CH_3).

Isolated yield: 77%.

2-methyl-1-(*p*-tolyl)propan-1-ol 8b: Prepared by following the general experimental procedure with:

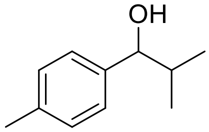

1 (4.95 mg, 2.0 mol%), 1-(*p*-tolyl)propan-1-ol **7b** (75.1 mg, 0.5 mmol), NaO^{t}Bu (96.1 mg, 1 mmol), toluene (0.8 mL), CO (5 bar), H_2 (15 bar) and reaction time: 24 h. Yield was determined by ^{1}H NMR spectrum using mesitylene (43.2 mg, 0.36 mmol) as an internal standard ($\delta_{Mesitylene(standard)}$ = 7.07 (s, 3H), $\delta_{product}$ = 4.53 (d, 1H)).The spectral data are in agreement with the reported literature.[5]

^{1}H NMR (400 MHz, $CDCl_3$, 298 K) δ =7.22 (d, 2H, ArC*H*), 7.17 (d, 2H, ArC*H*), 4.34 (d, 1H, *J* = 6.99 Hz, C*H*), 2.37 (s, 3H, CH_3), 1.92-2.01 (m, 1H, C*H*), 1.78 (br s, 1H, O*H*), 1.02 (d, 3H, *J* = 6.65 Hz, CH_3), 0.81 (d, 3H, *J* = 6.80 Hz, CH_3).

^{13}C{^{1}H}-NMR (101 MHz, CDCl3, 298 K) δ =140.81 (C_{Ar}), 137.16 (C_{Ar}), 129.00 (CH_{Ar}), 126.62 (CH_{Ar}), 80.09 (*C*H), 35.34 (*C*H), 21.25 (CH_3), 19.14 (CH_3), 18.50 (CH_3).

Isolated yield: 63%.

2-methyl-1,2,3,4-tetrahydronaphthalen-1-ol 8c: Prepared by following the general experimental procedure with: **1** (4.95 mg, 2.0 mol%), 1,2,3,4-tetrahydronaphthalen-1-ol **7c** (74.1 mg, 0.5 mmol), NaO^{t}Bu (96.1 mg, 1 mmol), toluene (0.8 mL), CO (5 bar), H_2 (15 bar) and reaction time: 36 h. Yield was determined by ^{1}H NMR spectrum using mesitylene (43.2 mg, 0.36 mmol) as an internal standard ($\delta_{Mesitylene(standard)}$ = 6.98 (s, 3H), $\delta_{product}$ = 4.72 (d, 1H), $\delta_{product}$ = 4.49 (d, 1H)). The spectral data are in agreement with the reported literature.[17, 18]

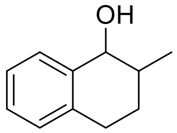

8. Standard procedure and reaction optimization for catalytic β-dimethylation of 1-phenylethanol

Mn-MACHO-^{i}Pr **1** (4.95 mg, 2 mol%) and NaO^{t}Bu were measured into a glass inlet equipped with a stirring bar inside a glovebox. The glass inlet was closed with a septum and transferred into the bottom part of the 10 mL steel autoclave, where it was opened under a stream of argon. After sealing, the autoclave was evacuated and purged with argon three times. Secondary alcohol (0.5 mmol) and toluene (0.8 mL) were added at room temperature through a valve under argon. The autoclave was sealed, pressurized with CO and H_2 and heated to 150 °C temperature. After the certain reaction time, the autoclave was cooled to room temperature and slowly vented while stirring continued. Mesitylene was added as an internal standard to the reaction mixture that was then passed through a short path of acidic alumina before the composition was analyzed by NMR spectroscopy.

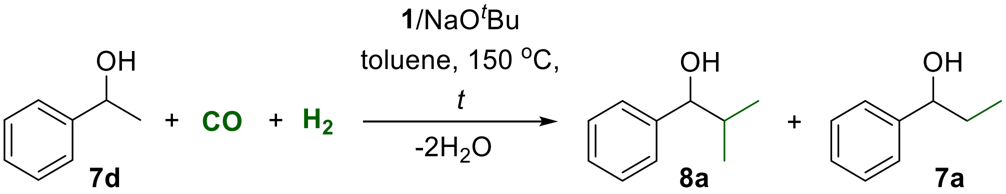

Table S2: Optimization reactions for β–methylation of **7d** using **1**.

Entry	CO (bar)	H_2 (bar)	Base (equiv.)	Time (h)	Conv. (%)^b	Yield (%) (8a: 7a)
1	5	15	NaO^{t}Bu (2)	24	>99	40 (73:27)
2	5	15	NaO^{t}Bu (4)	24	>99	64 (73:27)
3	5	15	NaO^{t}Bu (4)	36	>99	57 (100:0)
4	8	24	NaO^{t}Bu (4)	36	>99	78 (100:0)

9. Standard procedure for catalytic β-dimethylation of secondary alcohols

Mn-MACHO-^{i}Pr **1** (4.95 mg, 2 mol%) and NaO^{t}Bu (192.2 mg, 2 mmol) were measured into a glass inlet equipped with a stirring bar inside a glovebox. The glass inlet was closed with a septum and transferred into the bottom part of the 10 mL steel autoclave, where it was opened under a stream of argon. After sealing, the autoclave was evacuated and purged with argon three times. Secondary alcohol (0.5 mmol) and toluene (0.8 mL) were added at room temperature through a valve under argon. The autoclave was

sealed, pressurized with CO (8 bar) and H_2 (24 bar) and heated to 150 °C temperature. After 36 h, the autoclave was cooled to room temperature and slowly vented while stirring continued. Mesitylene was added as an internal standard to the reaction mixture that was then passed through a short path of acidic alumina before the composition was analyzed by NMR spectroscopy. The isolation of pure product was carried out using column chromatography over silica gel (100-200 mesh) using ethyl acetate/pentane (10 : 90) mixture as eluent.

2-methyl-1-phenylpropan-1-ol 8a: Prepared by following the general experimental procedure with: **1** (4.95 mg, 2.0 mol%), 1-phenylethanol **7d** (61.1 mg, 0.5 mmol), NaO^{t}Bu (192.2 mg, 2 mmol), toluene

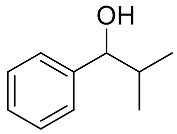

(0.8 mL), CO (8 bar), H_2 (24 bar) and reaction time: 36 h. Yield was determined by ^{1}H NMR spectrum using mesitylene (43.2 mg, 0.36 mmol) as an internal standard ($\delta_{Mesitylene(standard)}$ = 7.11 (s, 3H), $\delta_{product}$ = 4.60 (d, 1H)). The spectral data are in agreement with the reported literature.[5]

^{1}H NMR (400 MHz, $CDCl_3$, 298 K) δ = 7.20-7.29 (m, 5H, ArC*H*), 4.29 (d, 1H, *J* = 6.87 Hz, C*H*), 1.85-1.93 (m, 1H, C*H*), 1.78 (br s, 1H, O*H*), 0.93 (d, 3H, *J* = 6.67 Hz, CH_3), 0.73 (d, 3H, *J* =6.81 Hz, CH_3).

^{13}C{^{1}H}-NMR (101 MHz, CDCl3, 298 K) δ =143.78 (C_{Ar}), 128.32 (CH_{Ar}), 127.55 (CH_{Ar}), 126.70 (CH_{Ar}), 80.19 (*C*H), 35.40 (*C*H), 19.14 (CH_3), 18.37 (CH_3).

Isolated yield: 70%.

2-methyl-1-(naphthalen-2-yl)propan-1-ol 8d: Prepared by following the general experimental procedure

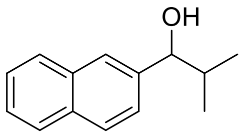

with: **1** (4.95 mg, 2.0 mol%), 1-(naphthalen-2-yl)ethanol **7e** (86.1 mg, 0.5 mmol), NaO^{t}Bu (192.2 mg, 2 mmol), toluene (0.8 mL), CO (8 bar), H_2 (24 bar) and reaction time: 36 h. Yield was determined by ^{1}H NMR spectrum using mesitylene (43.2 mg, 0.36 mmol) as an internal standard ($\delta_{Mesitylene(standard)}$ = 7.11 (s, 3H), $\delta_{product}$ = 4.60 (d, 1H)). The spectral data are in agreement with the reported literature.[15]

^{1}H NMR (300 MHz, $CDCl_3$, 298 K) δ = 7.75-7.85 (m, 3H, ArC*H*), 7.75 (br. s, 1H, ArC*H*), 7.45-7.50 (m, 3H, ArC*H*), 4.54 (d, 1H, *J* = 6.84 Hz, C*H*), 2.00-2.17 (m, 1H, C*H*), 1.90 (br. s, 1H, O*H*), 1.05 (d, 3H, *J* = 6.69 Hz, CH_3), 0.84 (d, 3H, *J* = 6.80 Hz. CH_3).

^{13}C{^{1}H}-NMR (75 MHz, CDCl3, 298 K) δ = 141.24 (C_{Ar}), 133.29 (C_{Ar}), 133.08 (C_{Ar}), 128.09 (CH_{Ar}), 128.06 (CH_{Ar}), 127.79 (CH_{Ar}), 126.19 (CH_{Ar}), 125.87 (CH_{Ar}), 125.54 (CH_{Ar}), 124.76 (CH_{Ar}), 80.30 (*C*H), 35.34 (*C*H), 19.29 (CH_3), 18.38 (CH_3).

Isolated yield: 48%.

10. Standard procedure for catalytic β-methylation of aliphatic alcohols

Mn-MACHO-^{i}Pr **1** (4.95 mg, 2 mol%) and NaO^{t}Bu (96.1 mg, 1 mmol) were measured into a glass inlet equipped with a stirring bar inside a glovebox. The glass inlet was closed with a septum and transferred into the bottom part of the 10 mL steel autoclave, where it was opened under a stream of argon. After sealing, the autoclave was purged with argon three times. Aliphatic alcohol (0.5 mmol) and toluene (0.8 mL) were added at room temperature through a valve under argon. The autoclave was sealed, pressurized with CO (5 bar) and H_2 (15 bar) and heated to 150 °C temperature. After the certain reaction time, the autoclave was cooled to room temperature and slowly vented while stirring continued. Mesitylene was added as an internal standard to the reaction mixture that was then passed through a short path of acidic alumina before the composition was analyzed by NMR spectroscopy. The isolation of pure product was carried out using column chromatography over silica gel (100-200 mesh) using ethyl acetate/pentane (10 : 90) mixture as eluent.

2-methylpropan-1-ol 10a: Prepared by following the general experimental procedure with: **1** (4.95 mg,

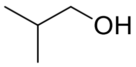

2.0 mol%), ethanol **9a** (23.0 mg, 0.5 mmol), NaO^{t}Bu (192.2 mg, 2 mmol), toluene (1.0 mL), CO (8 bar), H_2 (24 bar) and reaction time: 36 h. Yield was determined by ^{1}H NMR spectrum using mesitylene (43.2 mg, 0.36 mmol) as an internal standard ($\delta_{Mesitylene(standard)}$ = 6.69 (s, 3H), $\delta_{product}$ = 3.45 (d, 2H)). The spectral data are in agreement with the reported literature.[19]

^{1}H NMR (400 MHz, $CDCl_3$, 298 K) δ = 3.49 (d, 2H, *J* = 6.47 Hz, C*H*$_2$), 1.82-1.92 (m, 1H, C*H*), 1.53 (br. s, 1H, O*H*), 1.04 (d, 6H, C*H*$_3$).

2-methylpropan-1-ol 10a: Prepared by following the general experimental procedure with: **1** (4.95 mg,

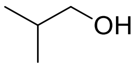

2.0 mol%), 1-propanol **9b** (30.0 mg, 0.5 mmol), NaO^{t}Bu (96.1 mg, 1 mmol), toluene (0.8 mL), CO (5 bar), H_2 (15 bar) and reaction time: 36 h. Yield was determined by ^{1}H NMR spectrum using mesitylene (43.2 mg, 0.36 mmol) as an internal standard ($\delta_{Mesitylene(standard)}$ = 7.07 (s, 3H), $\delta_{product}$ = 1.17 (d, 3H)). The spectral data are in agreement with the reported literature.[19]

^{1}H NMR (400 MHz, $CDCl_3$, 298 K) δ =3.49 (d, 2H, *J* = 6.47 Hz, C*H*$_2$), 1.82-1.92 (m, 1H, C*H*), 1.53 (br. s, 1H, O*H*), 1.04 (d, 6H, C*H*$_3$).

2-methylbutan-1-ol 10b: Prepared by following the general experimental procedure with: **1** (4.95 mg,

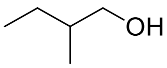

2.0 mol%), 1-butanol **9c** (37.1 mg, 0.5 mmol), NaO^{t}Bu (96.1 mg, 1 mmol), toluene

(0.8 mL), CO (5 bar), H_2 (15 bar) and reaction time: 36 h. Yield was determined by ^{1}H NMR spectrum using mesitylene (43.2 mg, 0.36 mmol) as an internal standard ($\delta_{Mesitylene(standard)}$ = 7.08 (s, 3H), $\delta_{product}$ = 3.62-3.75 (ddd, 2H)). The spectral data are in agreement with the reported literature.[20]

^{1}H NMR (400 MHz, $CDCl_3$, 298 K) δ = 3.37-3.51 (ddd, 2H, *J* = 6.15, 10.50, 37.05 Hz, C*H*$_2$), 1.79 (br s, 1H, O*H*), 1.38-1.58 (m, 2H, C*H*$_2$), 1.04-1.18 (m, 1H, C*H*), 0.87-0.91 (m, 6H, C*H*$_3$).

^{13}C{^{1}H}-NMR (101 MHz, $CDCl_3$, 298 K) δ = 68.11 (*C*H$_2$), 37.46 (*C*H), 25.86 (*C*H$_2$), 16.21 (*C*H$_3$), 11.43 (*C*H$_3$).

Isolated yield: 66%

2-methylpentan-1-ol 10c: Prepared by following the general experimental procedure with: **1** (4.95 mg, 2.0 mol%), 1-pentanol **9d** (44.1 mg, 0.5 mmol), NaO^{t}Bu (96.1 mg, 1 mmol), toluene (0.8 mL), CO (5 bar), H_2 (15 bar) and reaction time: 36 h. Yield was determined by ^{1}H NMR spectrum using mesitylene (43.2 mg, 0.36 mmol) as an internal standard ($\delta_{Mesitylene(standard)}$ = 7.08 (s, 3H), $\delta_{product}$ = 3.60-3.74 (ddd, 2H)).The spectral data are in agreement with the reported literature.[21]

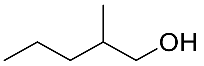

^{1}H NMR (400 MHz, $CDCl_3$, 298 K) δ = 3.34-3.49 (m, 2H, C*H*$_2$), 1.91 (br s, 1H, O*H*), 1.56-1.64 (m, 1H, O*H*), 1.21-1.40 (m, 3H, C*H*$_2$ & C*H*), 1.03-1.12 (m, 1H, C*H*), 0.86-0.89 (m, 6H, C*H*$_3$).

^{13}C{^{1}H}-NMR (101 MHz, $CDCl_3$, 298 K) δ = 68.40 (*C*H$_2$), 35.57 (*C*H), 35.52 (CH$_2$), 20.16 (*C*H$_2$), 16.63 (*C*H$_3$), 14.41 (*C*H$_3$).

Isolated yield: 71%

2-methylhexan-1-ol 10d: Prepared by following the general experimental procedure with: **1** (4.95 mg, 2.0 mol%), 1-hexanol **9e** (51.1 mg, 0.5 mmol), NaO^{t}Bu (96.1 mg, 1 mmol), toluene (0.8 mL), CO (5 bar), H_2 (15 bar) and reaction time: 36 h. Yield was determined by ^{1}H NMR spectrum using mesitylene (43.2 mg, 0.36 mmol) as an internal standard ($\delta_{Mesitylene(standard)}$ = 7.05 (s, 3H), $\delta_{product}$ = 3.58-3.72 (ddd, 2H)). The spectral data are in agreement with the reported literature.[22]

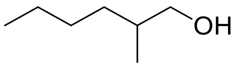

^{1}H NMR (400 MHz, $CDCl_3$, 298 K) δ = 3.54-3.68 (ddd, 2H, *J* = 6.26, 10.34, 38.20 Hz, C*H*$_2$), 1.74-1.80 (m, 1H, C*H*), 1.51-1.56 (m, 6H, C*H*$_2$ & C*H*), 1.25-1.34 (m, 1H, O*H*), 1.11-1.15 (m, 6H, C*H*$_3$).

2-methyloctan-1-ol 10e: Prepared by following the general experimental procedure with: **1** (4.95 mg, 2.0 mol%), 1-octanol **9f** (65.1 mg, 0.5 mmol), NaO^{t}Bu (96.1 mg, 1 mmol), toluene (0.8 mL), CO (5 bar), H_2 (15 bar) and reaction time: 36 h. Yield was determined by ^{1}H NMR spectrum using mesitylene (43.2 mg, 0.36 mmol) as an internal standard ($\delta_{Mesitylene(standard)}$ = 7.01 (s, 3H), $\delta_{product}$ = 3.55-3.66 (ddd, 2H)).The spectral data are in agreement with the reported literature.[23]

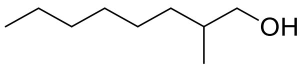

^{1}H NMR (400 MHz, $CDCl_3$, 298 K) δ = 3.39-3.53 (ddd, 2H, *J* = 6.16, 10.47, 37.8 Hz, C*H*$_2$), 1.55-1.65 (m, 1H, C*H*), 1.42 (m, 1H, O*H*), 1.08 -1.27 (m, 10H, C*H*$_2$) 0.91 (d, 3H, *J* = 6.72 Hz, C*H*$_3$), 0.88 (t, 3H, *J* = 6.78 Hz, CH_3).
^{13}C{^{1}H}-NMR (101 MHz, $CDCl_3$, 298 K) δ = 68.60 (*C*H$_2$), 35.92 (*C*H), 33.30 (*C*H$_2$), 32.01 (*C*H$_2$), 29.76 (*C*H$_2$), 27.09 (*C*H$_2$), 22.82 (*C*H$_2$), 16.74 (*C*H$_3$), 14.26 (*C*H$_3$).
Isolated yield: 73%.

2-methyldecan-1-ol 10f: Prepared by following the general experimental procedure with: **1** (4.95 mg,

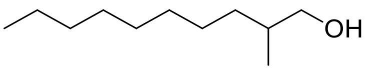

2.0 mol%), 1-decanol **9g** (79.1 mg, 0.5 mmol), NaO^{t}Bu (96.1 mg, 1 mmol), toluene (0.8 mL), CO (5 bar), H_2 (15 bar) and reaction time: 24 h. Yield was determined by ^{1}H NMR spectrum using mesitylene (43.2 mg, 0.36 mmol) as an internal standard ($\delta_{Mesitylene(standard)}$ = 7.10 (s, 3H), $\delta_{product}$ = 3.61-3.76 (ddd, 2H)). The spectral data are in agreement with the reported literature.[24]
^{1}H NMR (400 MHz, $CDCl_3$, 298 K) δ = 3.39-3.52 (ddd, 2H, *J* = 6.14, 10.45, 38.25 Hz, C*H*$_2$), 1.55-1.62 (m, 1H, C*H*), 1.26-1.30 (m, 14H, C*H*$_2$), 1.06-1.12 (m, 1H, O*H*), 0.86-0.92 (m, 6H, C*H*$_3$).
^{13}C{^{1}H}-NMR (101 MHz, $CDCl_3$, 298 K) δ = 68.57 (*C*H$_2$), 35.91 (*C*H), 33.29 (*C*H$_2$), 32.04 (*C*H$_2$), 30.09 (*C*H$_2$), 29.74 (*C*H$_2$), 29.47 (*C*H$_2$), 27.12 (*C*H$_2$), 22.81 (*C*H$_2$), 16.72 (*C*H$_3$), 14.25 (*C*H$_3$).
Isolated yield: 73%.

2-methylundec-10-en-1-ol 10g: Prepared by following the general experimental procedure with: **1**

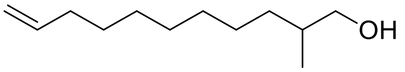

(4.95 mg, 2.0 mol%), Undec-10-en-1-ol **9h** (85.2 mg, 0.5 mmol), NaO^{t}Bu (96.1 mg, 1 mmol), toluene (0.8 mL), CO (5 bar), H_2 (15 bar) and reaction time: 24 h. Yield was determined by ^{1}H NMR spectrum using mesitylene (43.2 mg, 0.36 mmol) as an internal standard ($\delta_{Mesitylene(standard)}$ = 7.03 (s, 3H), $\delta_{product}$ = 3.57-3.70 (ddd, 2H)). The spectral data are in agreement with the reported literature.[25]
^{1}H NMR (400 MHz, $CDCl_3$, 298 K) δ = 5.76-5.86 (m, 1H, C*H*), 4.91-5.01 (m, 2H, C*H*$_2$), 3.39-3.52 (ddd, 2H, *J* = 6.15, 10.42, 37.27 Hz, C*H*$_2$), 2.01-2.06 (dd, 2H, *J* = 6.87, 14.22 Hz, C*H*$_2$), 1.56-1.62 (m, 1H, C*H*), 1.28-1.39 (m, 12H, C*H*$_2$), 1.06-1.13 (m, 1H, O*H*), 0.91 (d, 3H, *J* = 6.69 Hz, C*H*$_3$).
^{13}C{^{1}H}-NMR (101 MHz, $CDCl_3$, 298 K) δ = 139.37 (*C*H), 114.25 (*C*H$_2$), 68.57 (*C*H$_2$), 35.91 (*C*H), 33.94 (*C*H$_2$), 33.27 (*C*H$_2$), 30.01 (*C*H$_2$), 29.60 (*C*H$_2$), 29.26 (*C*H$_2$), 29.06 (*C*H$_2$), 27.10 (*C*H$_2$), 16.72 (*C*H$_3$).
Isolated yield: 61%.

2-methyldodecan-1-ol 10h: Prepared by following the general experimental procedure with: **1** (4.95 mg,

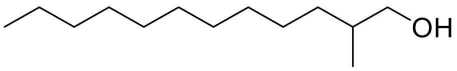

2.0 mol%), dodecan-1-ol **9i** (93.2 mg, 0.5 mmol), NaO^{t}Bu

(96.1 mg, 1 mmol), toluene (0.8 mL), CO (5 bar), H_2 (15 bar) and reaction time: 24 h. Yield was determined by ^{1}H NMR spectrum using mesitylene (43.2 mg, 0.36 mmol) as an internal standard ($\delta_{Mesitylene(standard)}$ = 6.99 (s, 3H), $\delta_{product}$ = 3.53-3.68 (ddd, 2H)). The spectral data are in agreement with the reported literature.[26]

^{1}H NMR (400 MHz, $CDCl_3$, 298 K) δ = 3.32-3.46 (ddd, 2H, *J* = 6.08, 10.41, 38.64 Hz, C*H*), 1.48-1.57 (m, 1H, CH), 1.19-1.23 (m, 18H, C*H₂*), 1.01-1.04 (m, 1H, O*H*), 0.79-0.85 (m, 6H, C*H₃*).

^{13}C{^{1}H}-NMR (101 MHz, $CDCl_3$, 298 K) δ = 68.56 (*C*H₂), 35.90 (*C*H), 33.28 (*C*H₂), 32.06 (*C*H₂), 30.09 (*C*H₂), 29.81 (*C*H₂), 29.80 (*C*H₂), 29.78 (*C*H₂), 29.76 (*C*H₂), 29.49 (*C*H₂), 27.12 (*C*H₂), 22.83 (*C*H₂), 16.72 (*C*H₃), 14.26 (*C*H₃).

Isolated yield: 85%.

2-methyloctadecan-1-ol 10i: Prepared by following the general experimental procedure with: **1** (4.95 mg, 2.0 mol%), octadecan-1-ol **9j** (135.3 mg, 0.5 mmol), NaO^{t}Bu (96.1 mg, 1 mmol), toluene (0.8 mL), CO (5 bar), H_2 (15 bar) and reaction time: 24 h. Yield was determined by ^{1}H NMR spectrum using mesitylene (43.2 mg, 0.36 mmol) as an internal standard ($\delta_{Mesitylene(standard)}$ = 7.0 (s, 3H), $\delta_{product}$ = 3.55-3.68 (ddd, 2H)).

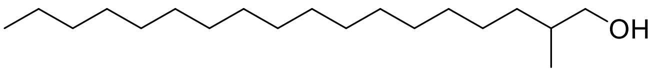

^{1}H NMR (400 MHz, $CDCl_3$, 298 K) δ = 3.32-3.46 (ddd, 2H, *J* = 6.11, 10.45, 38.34, C*H*), 1.48-1.58 (m, 1H, C*H*), 1.19-1.23 (m, 30H, CH2), 0.99-1.08 (m, 1H, O*H*), 0.79-0.85 (m, 6H, C*H₃*).

^{13}C{^{1}H}-NMR (101 MHz, $CDCl_3$, 298 K) δ = 68.58 (*C*H₂), 35.91 (*C*H), 33.29 (*C*H₂), 32.08 (*C*H₂), 30.10 (*C*H₂), 29.85 (*C*H₂), 29.84 (*C*H₂), 29.81 (*C*H₂), 29.52 (*C*H₂), 27.13 (*C*H₂), 22.85 (*C*H₂), 16.73 (*C*H₃), 14.28 (*C*H₃).

Isolated yield: 71%.

11. Postulated Catalytic Sequence

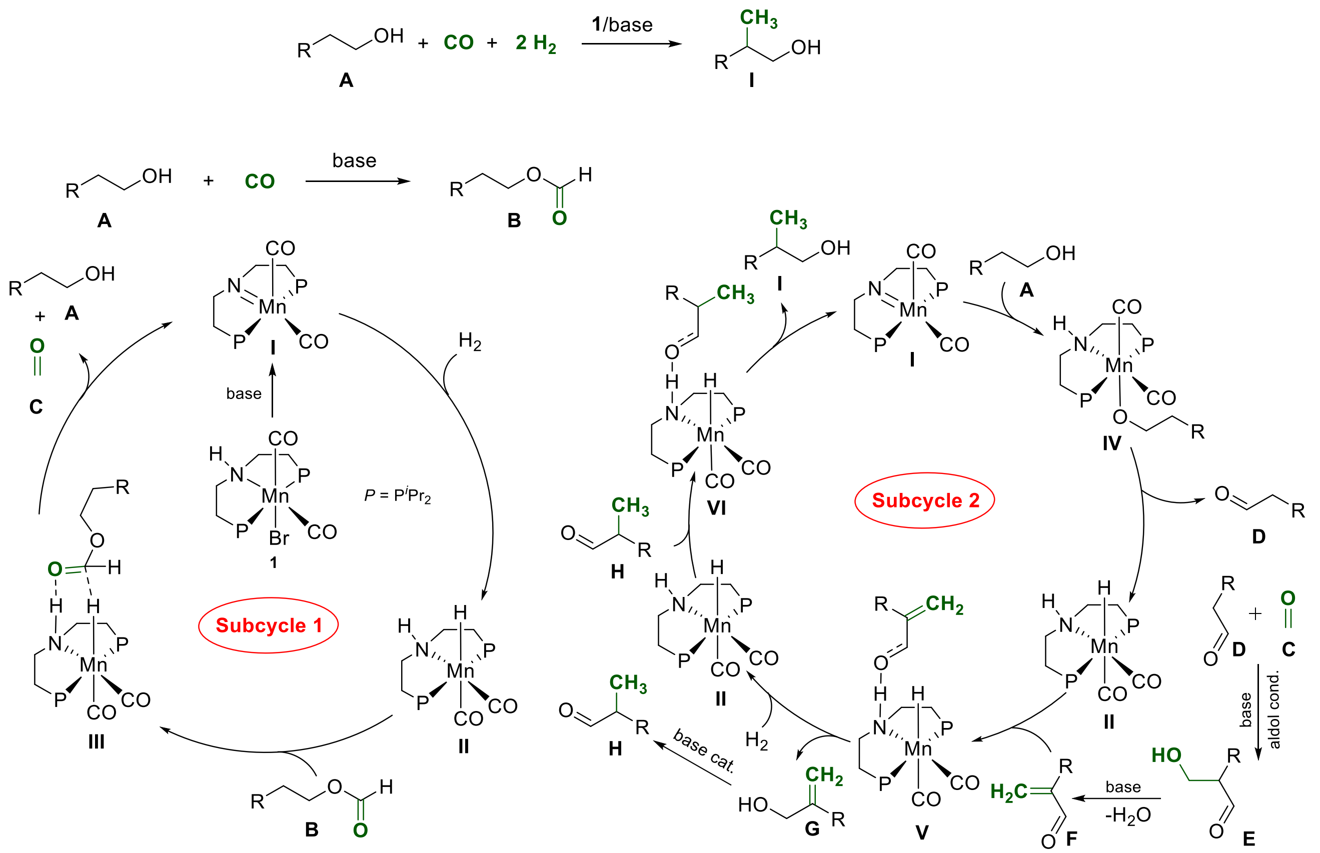

12. Alternate reaction mechanism

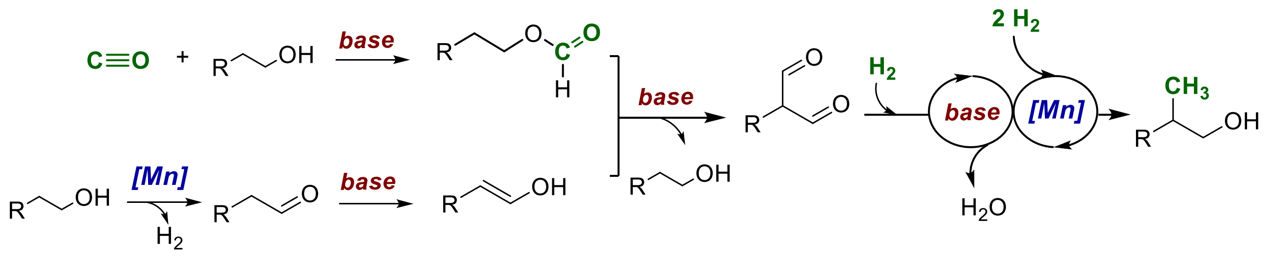

13. NMR spectra of isolated products

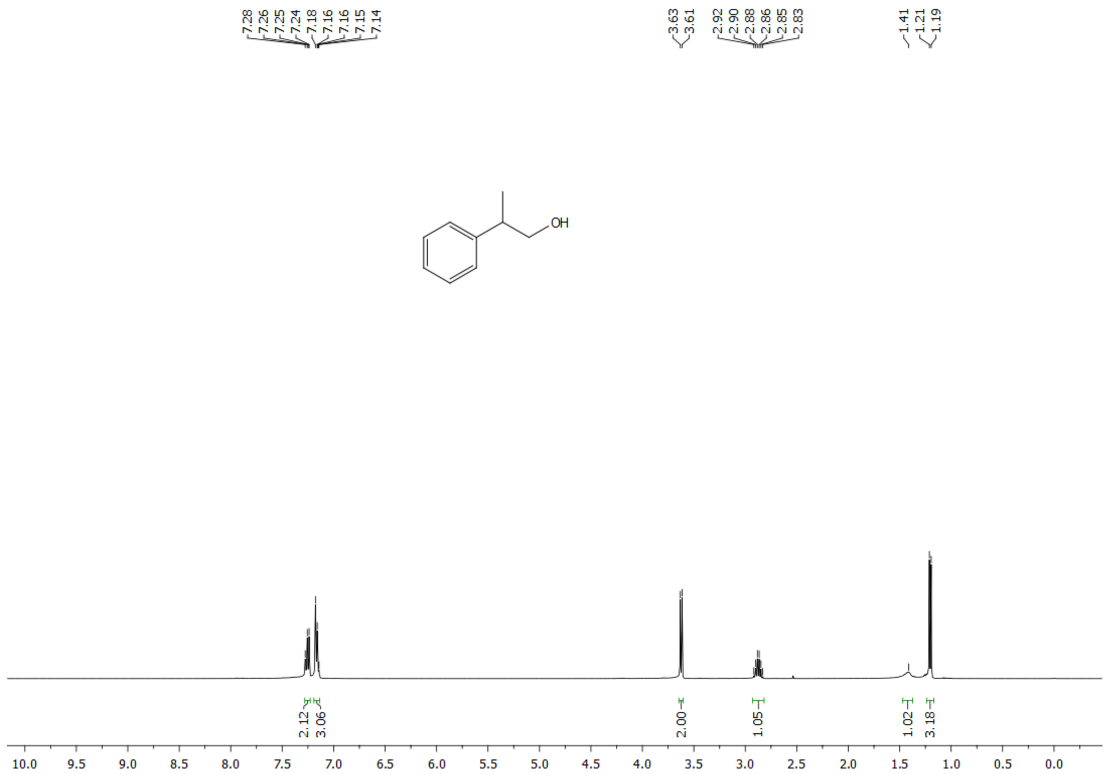

Figure S15: ^{1}H NMR (400 MHz, $CDCl_3$, 298 K) spectrum of 2-phenylpropan-1-ol 3a.

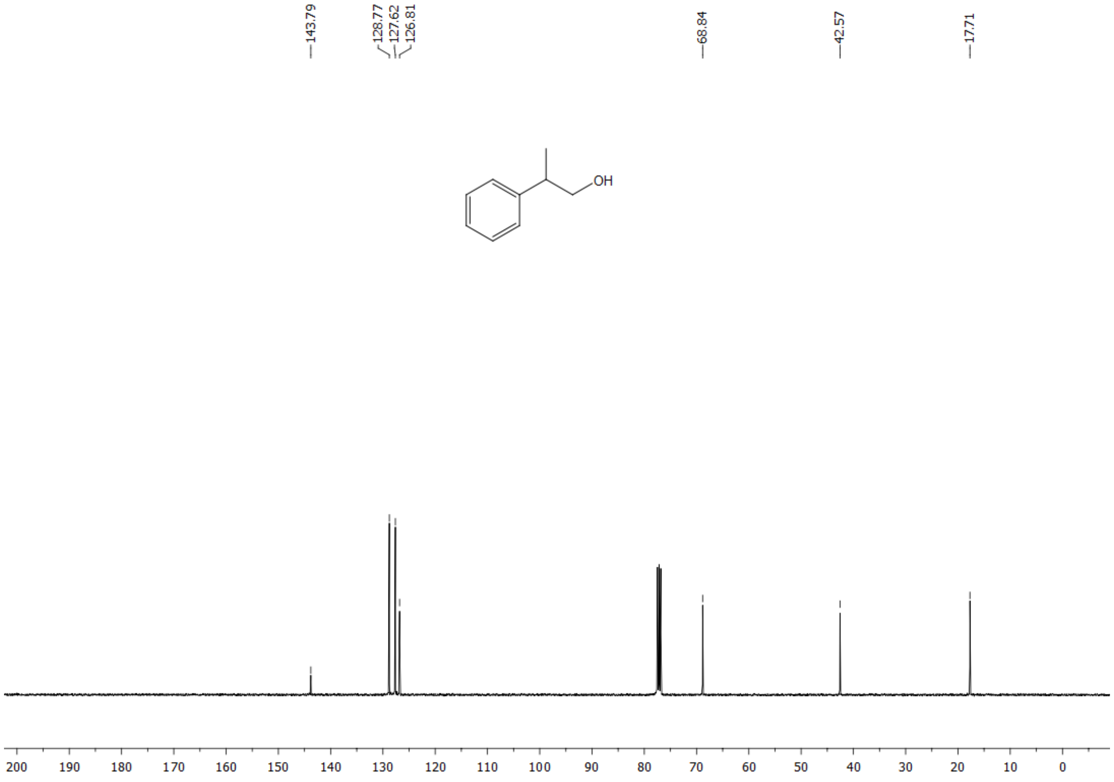

Figure S16: $^{13}C\{^1H\}$ NMR (101 MHz, $CDCl_3$, 298 K) spectrum of 2-phenylpropan-1-ol 3a.

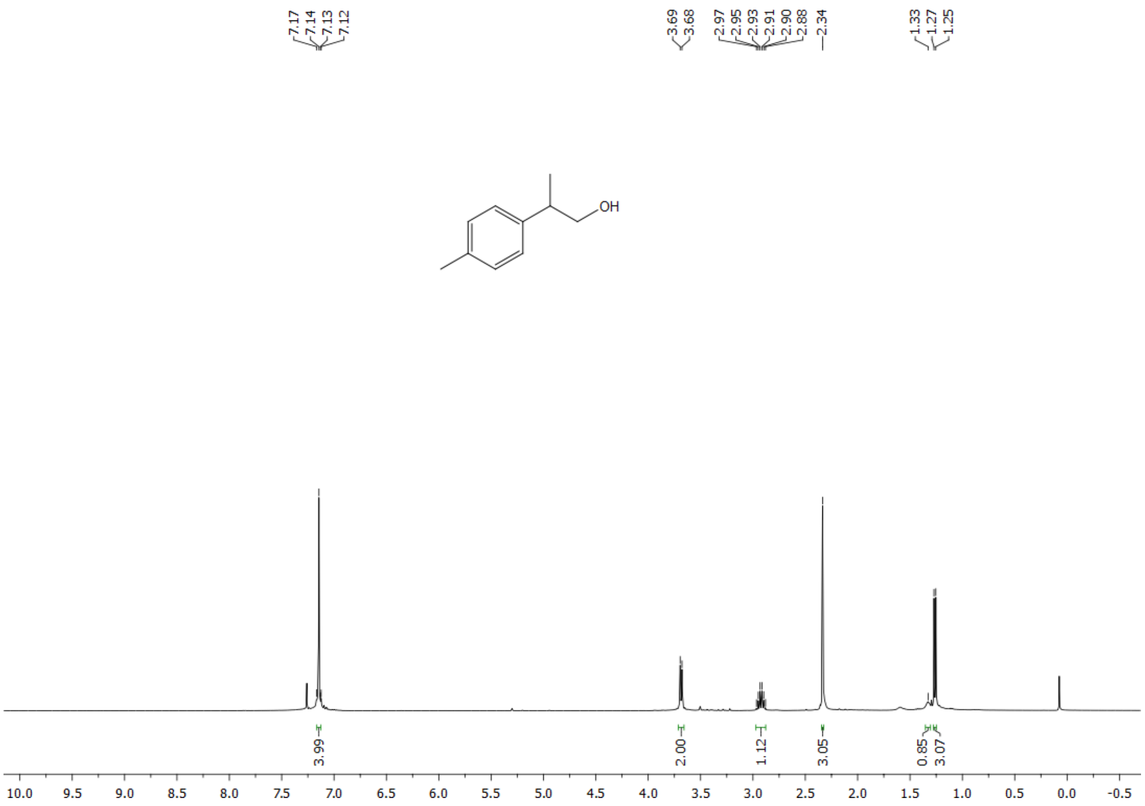

Figure S17: ^{1}H NMR (400 MHz, $CDCl_3$, 298 K) spectrum of 2-(*p*-tolyl)propan-1-ol 3b.

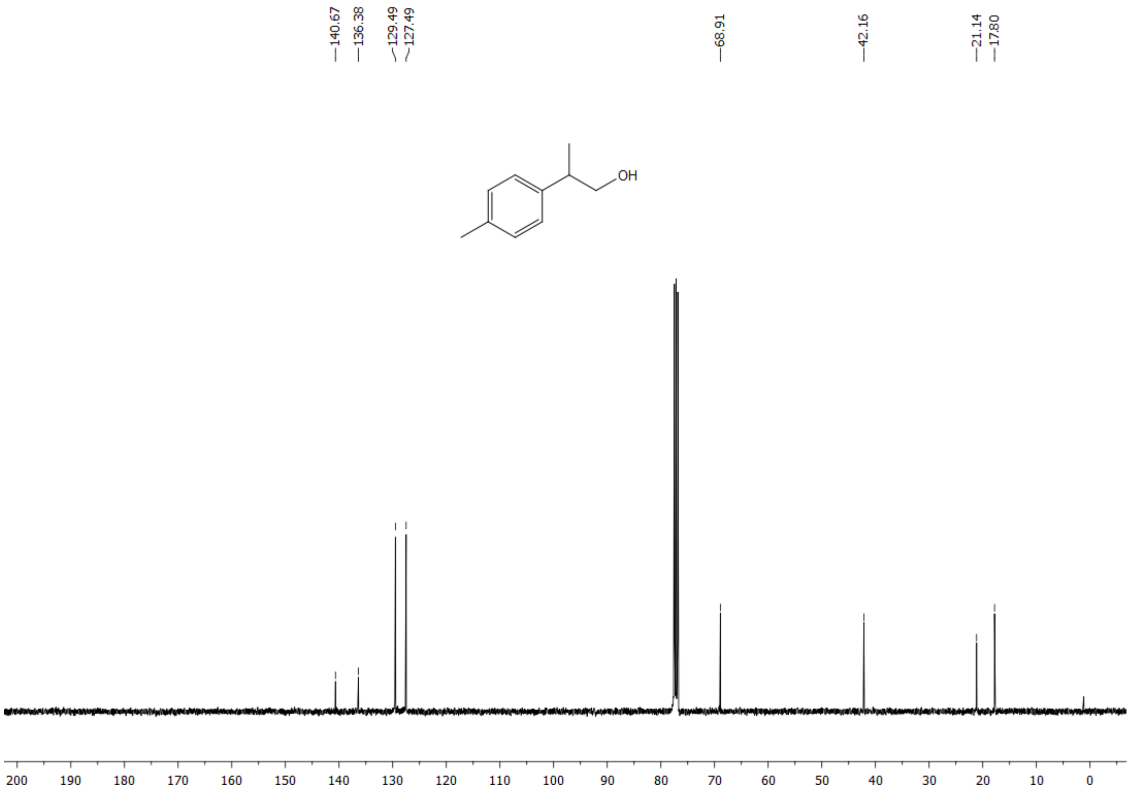

Figure S18: ^{13}C{^{1}H} NMR (101 MHz, $CDCl_3$, 298 K) spectrum of 2-(*p*-tolyl)propan-1-ol 3b.

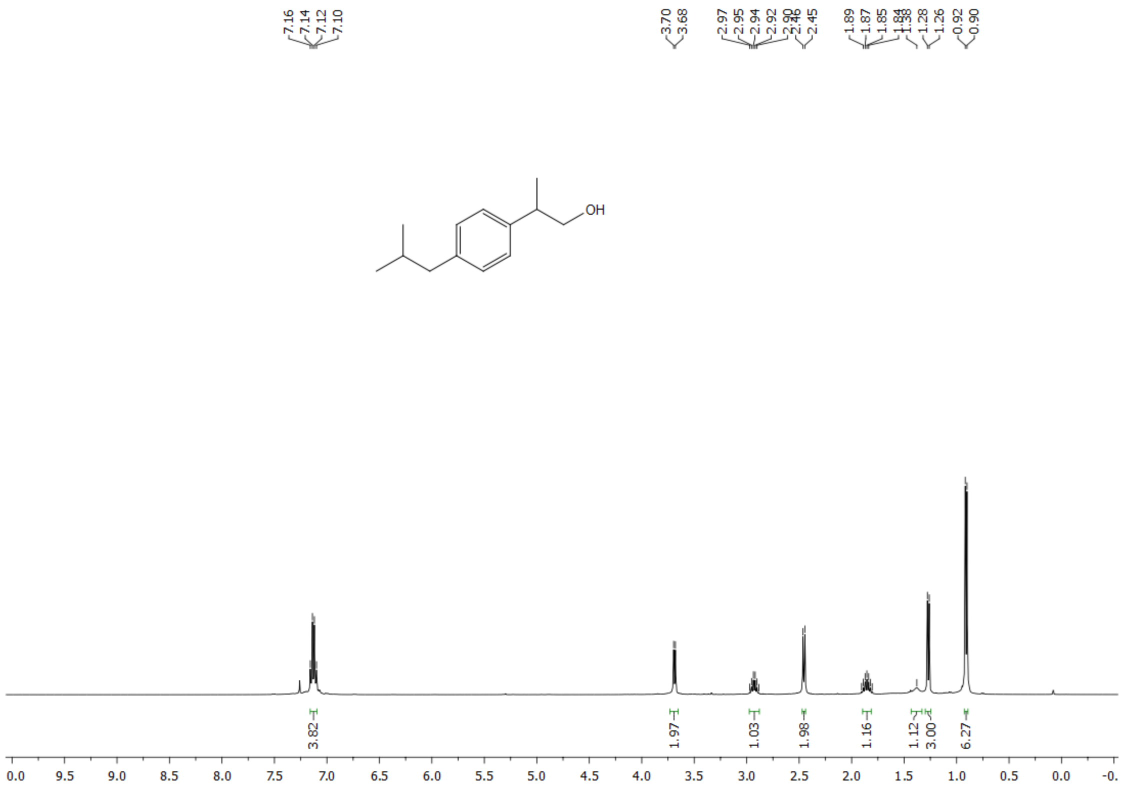

Figure S19: ^{1}H NMR (400 MHz, $CDCl_3$, 298 K) spectrum of 2-(4-isobutylphenyl)ethanol 3c.

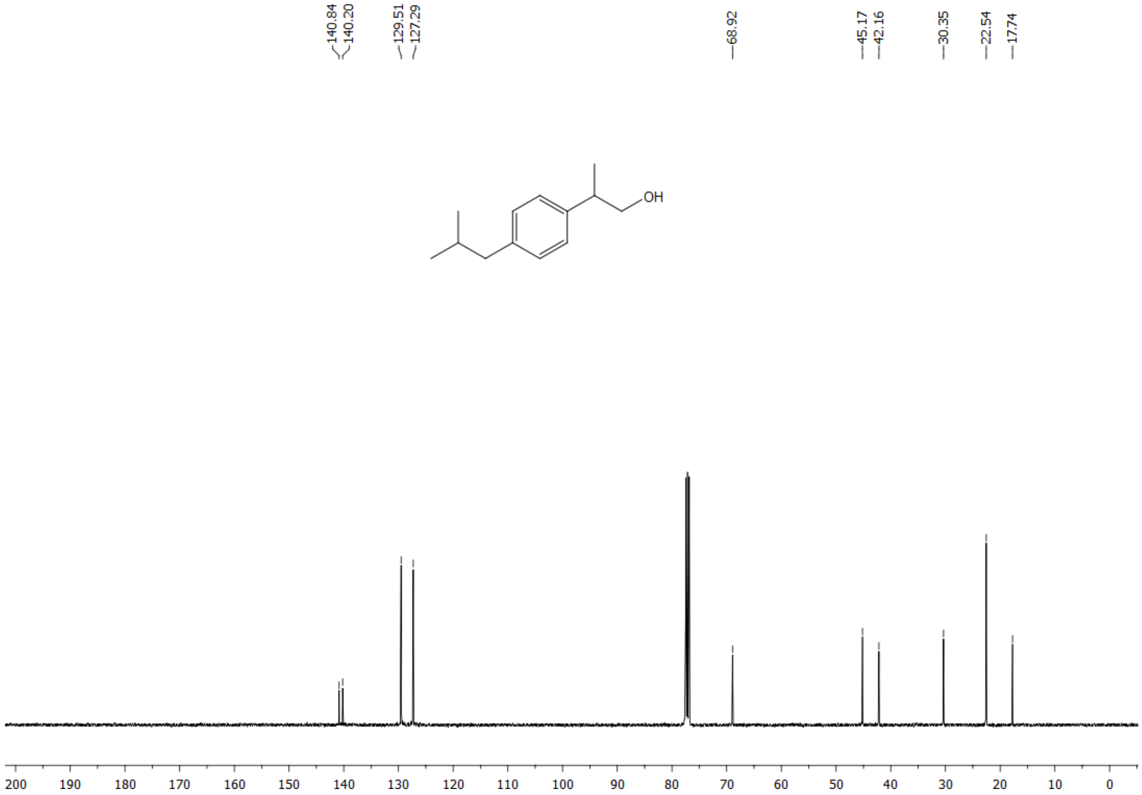

Figure S20: $^{13}C\{^{1}H\}$ NMR (101 MHz, $CDCl_3$, 298 K) spectrum of 2-(4-isobutylphenyl)ethanol 3c.

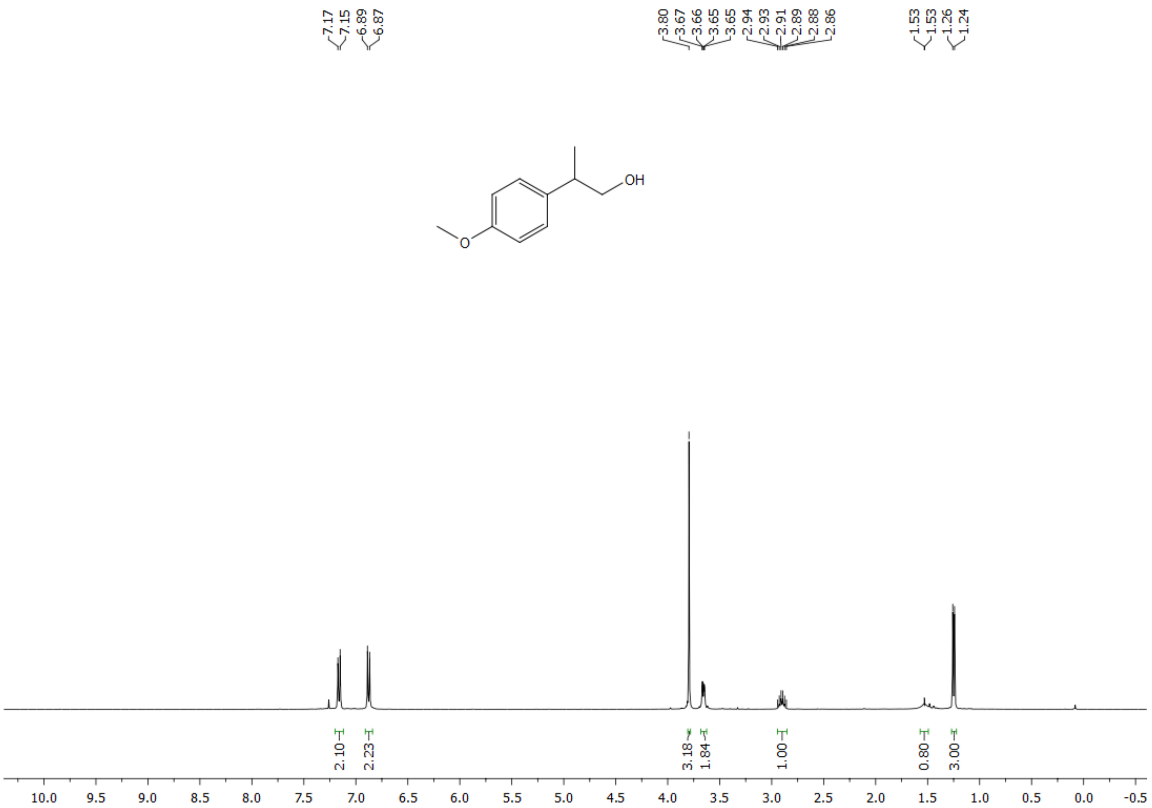

Figure S21: ^{1}H NMR (400 MHz, $CDCl_3$, 298 K) spectrum of 2-(4-methoxyphenyl)propan-1-ol 3d.

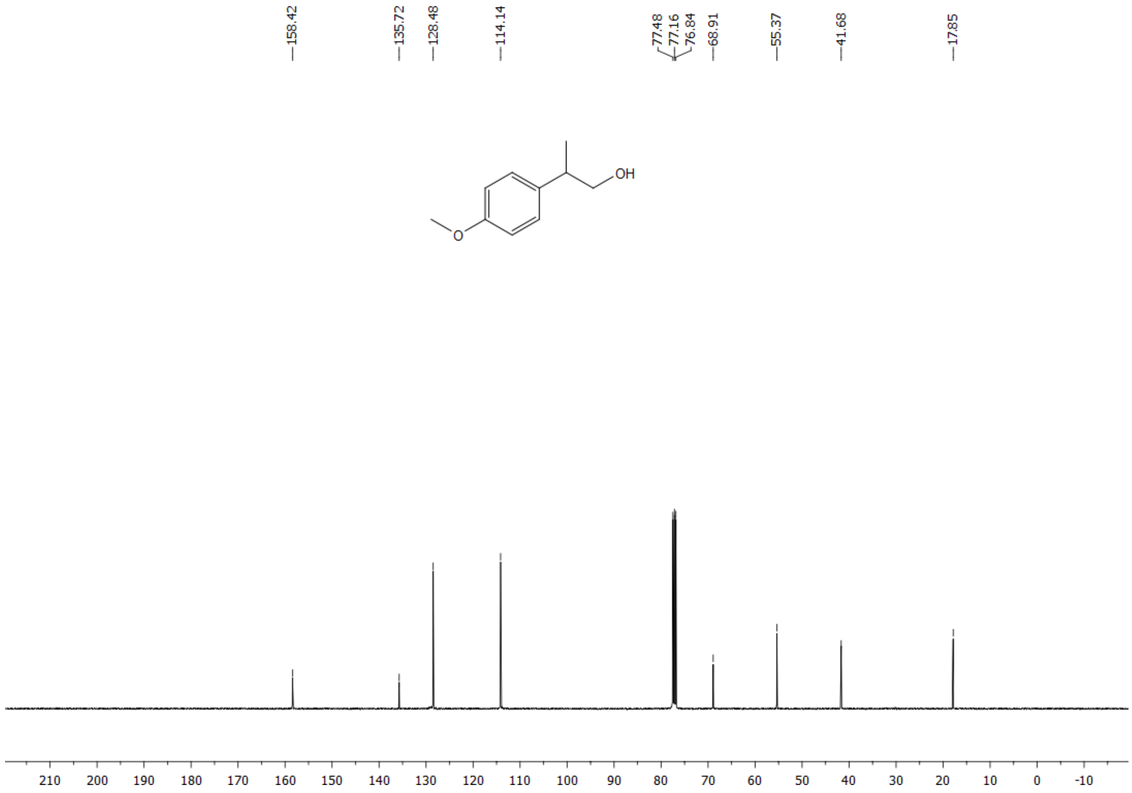

Figure S22: $^{13}C\{^{1}H\}$ NMR (101 MHz, $CDCl_3$, 298 K) spectrum of 2-(4-methoxyphenyl)propan-1-ol 3d.

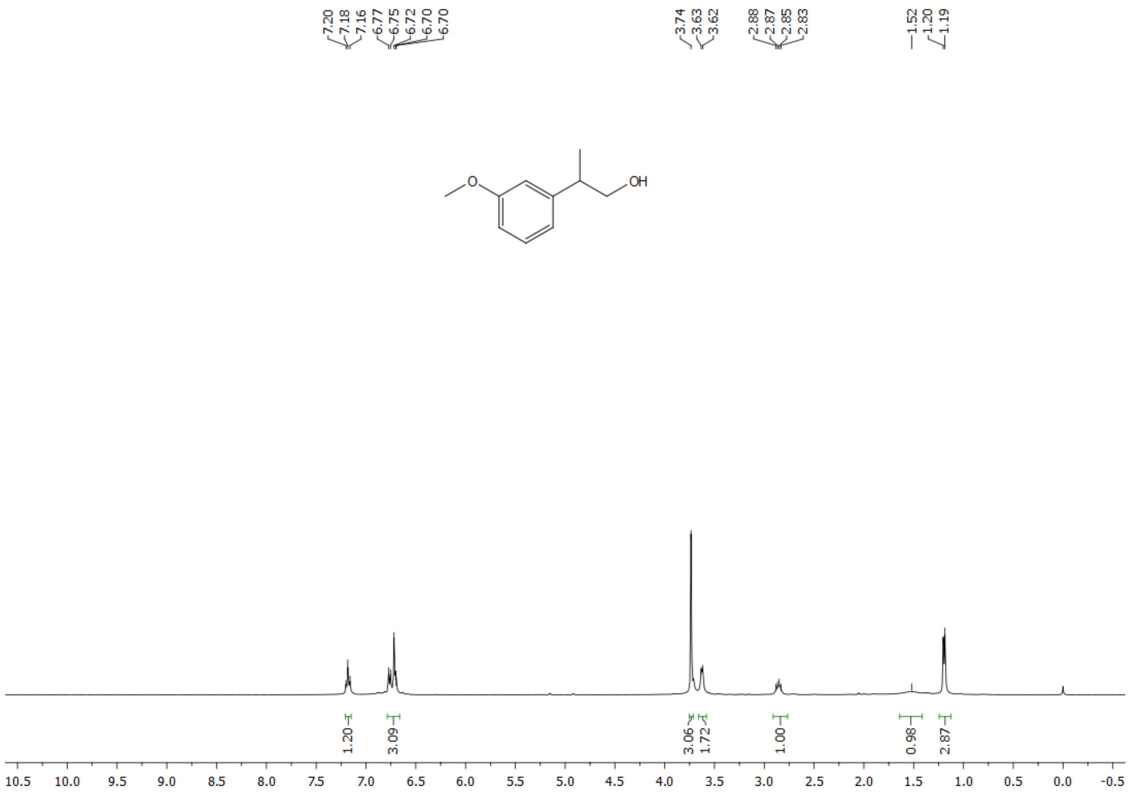

Figure S23: ^{1}H NMR (400 MHz, $CDCl_3$, 298 K) spectrum of 2-(3-methoxyphenyl)propan-1-ol 3e.

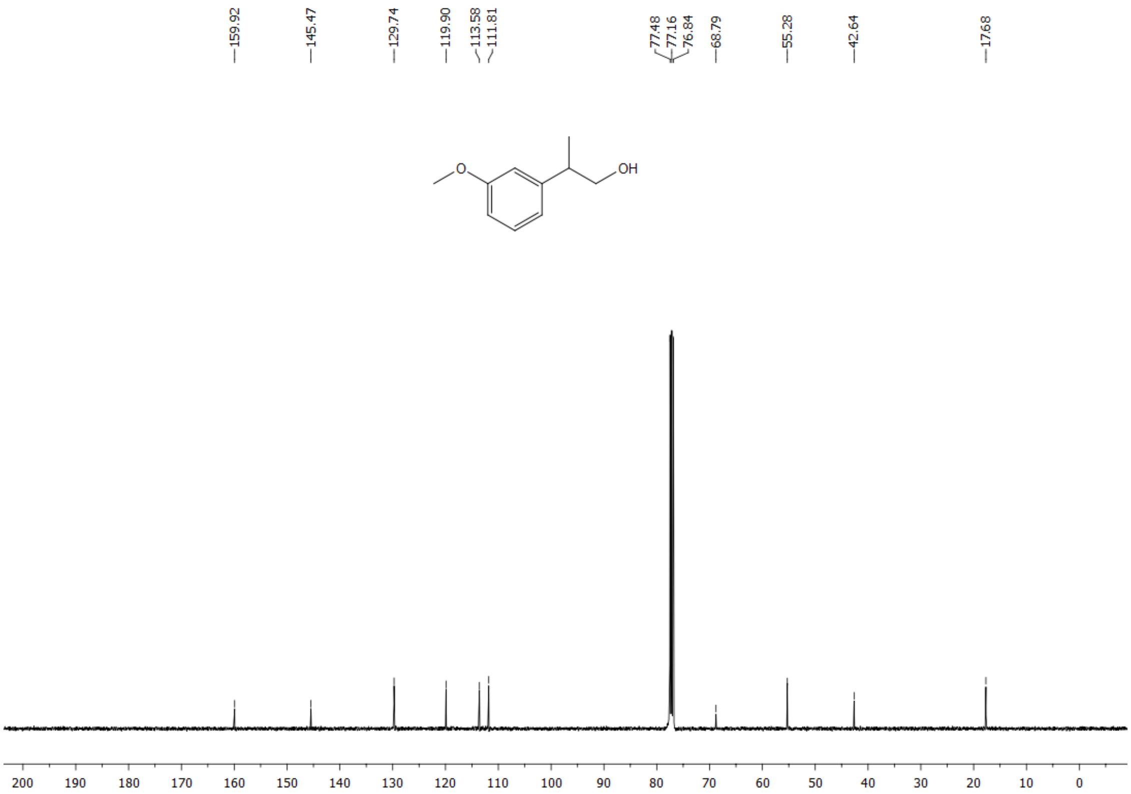

Figure S24:^{13}C{^{1}H} NMR (101 MHz, $CDCl_3$, 298 K) spectrum of 2-(3-methoxyphenyl)propan-1-ol 3e.

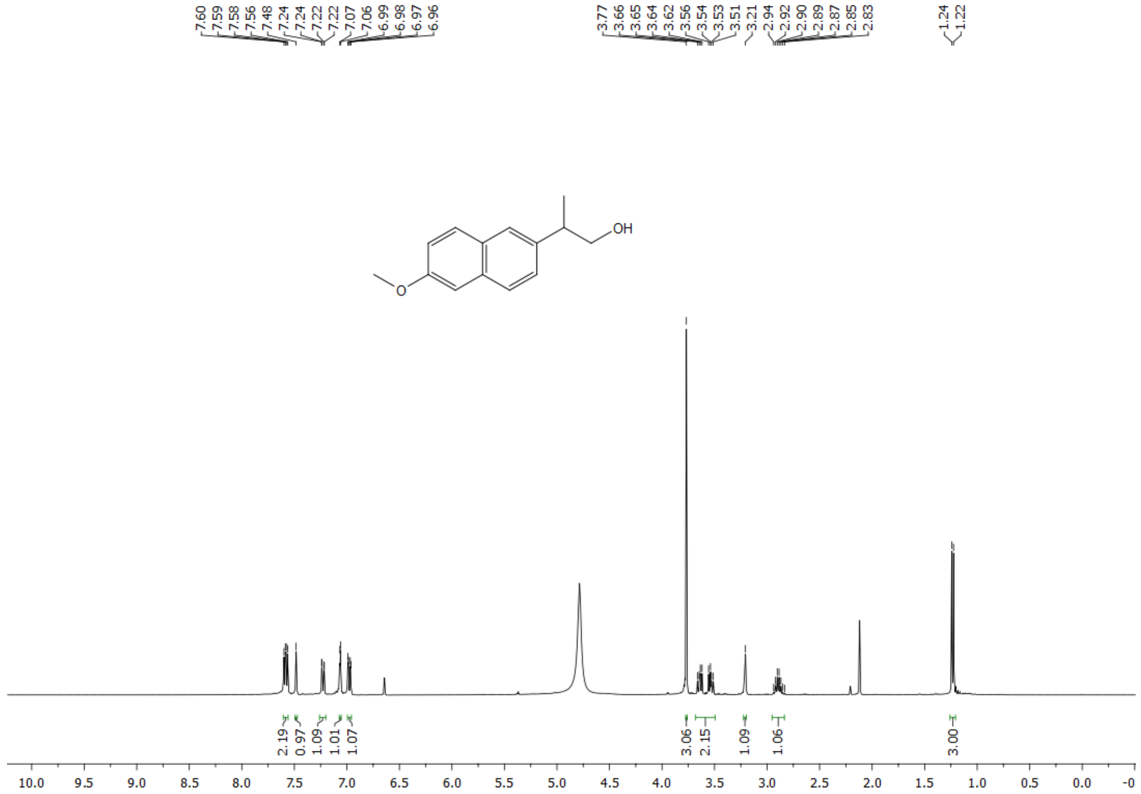

Figure S25: ^{1}H NMR (400 MHz, CD_3OD, 298 K) spectrum of 2-(6-methoxynaphthalen-2-yl)propan-1-ol 3f.

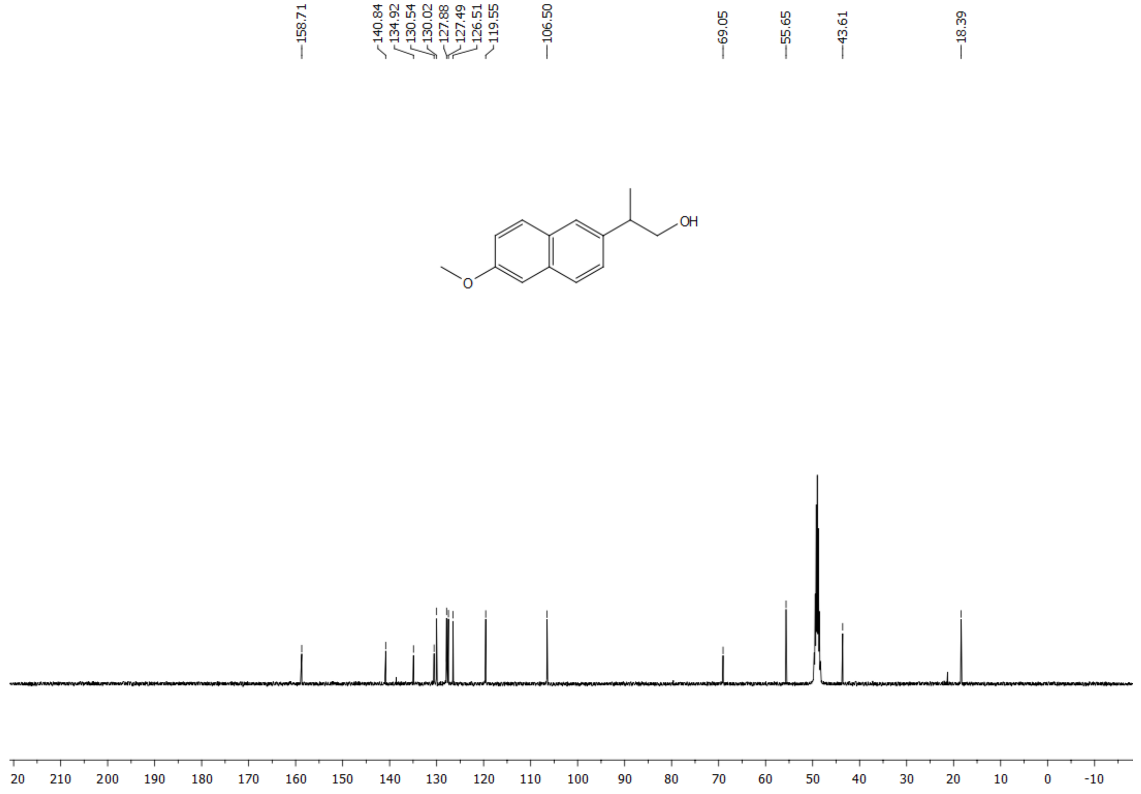

Figure S26: ^{13}C{^{1}H} NMR (101 MHz, CD_3OD, 298 K) spectrum of 2-(6-methoxynaphthalen-2-yl)propan-1-ol 3f.

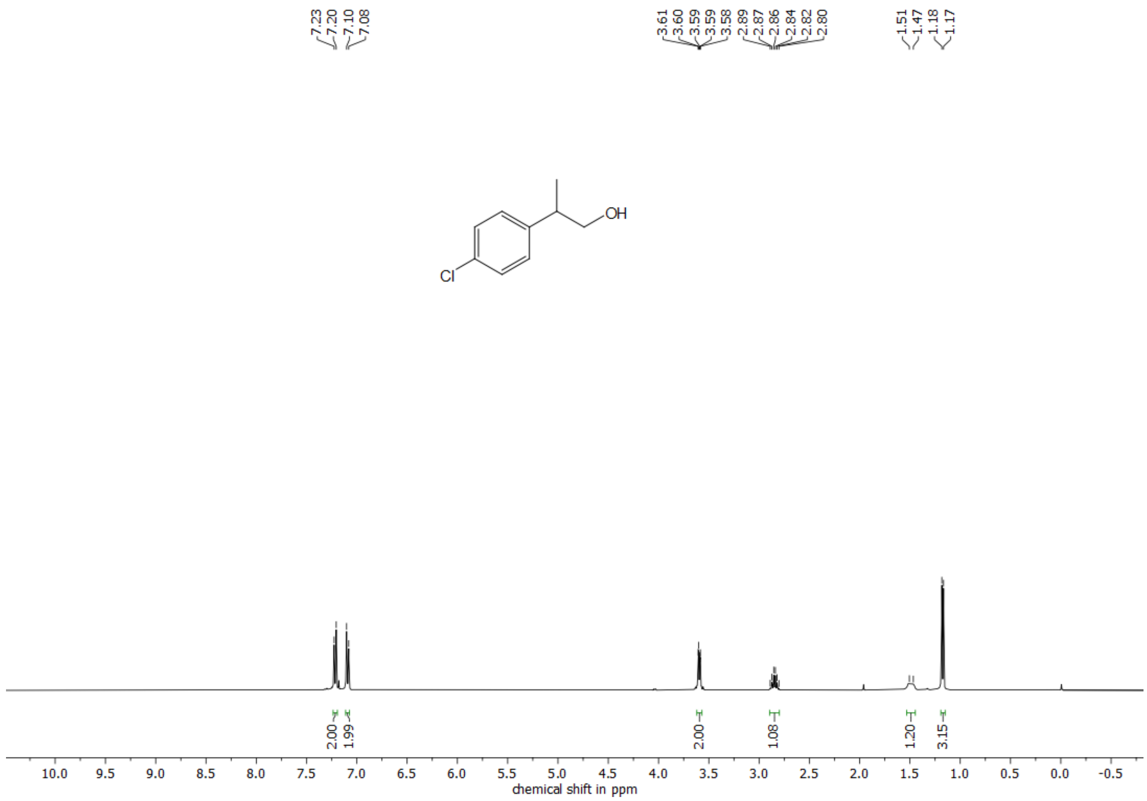

Figure S27:^{1}H NMR (400 MHz, $CDCl_3$, 298 K) spectrum of 2-(4-chlorophenyl)propan-1-ol 3g.

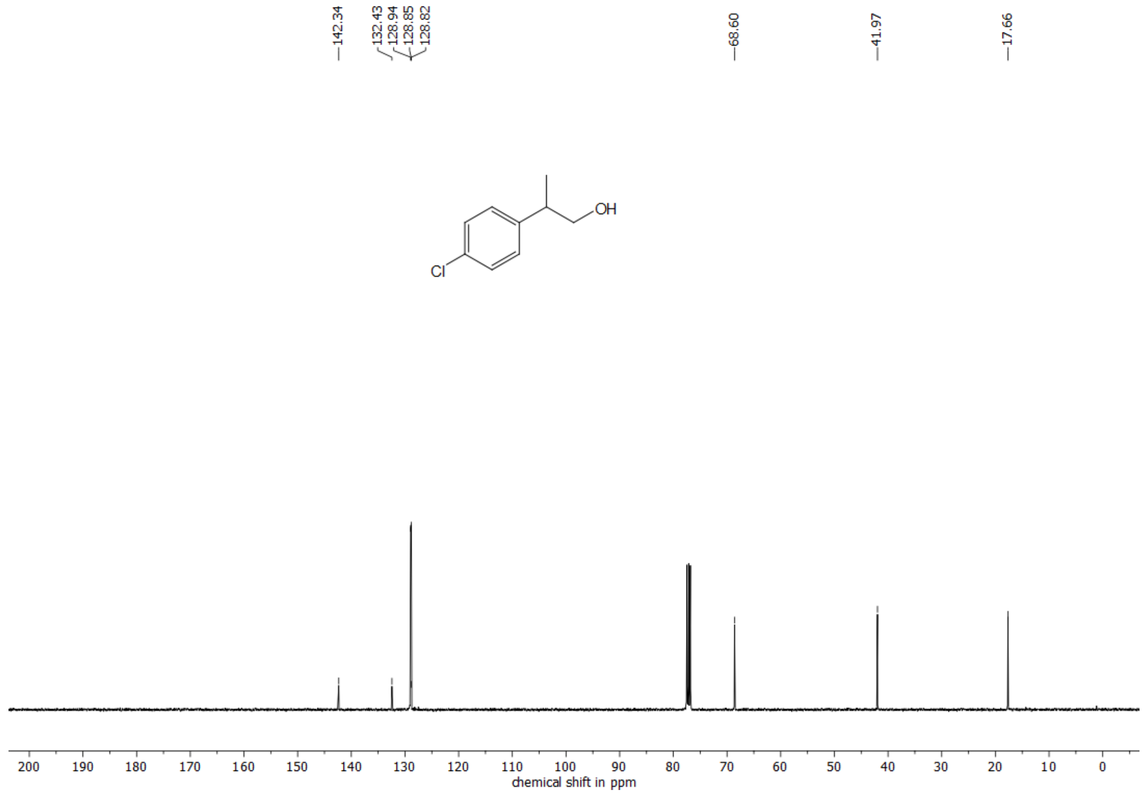

Figure S28:^{13}C{^{1}H} NMR (101 MHz, $CDCl_3$, 298 K) spectrum of 2-(4-chlorophenyl)propan-1-ol 3g.

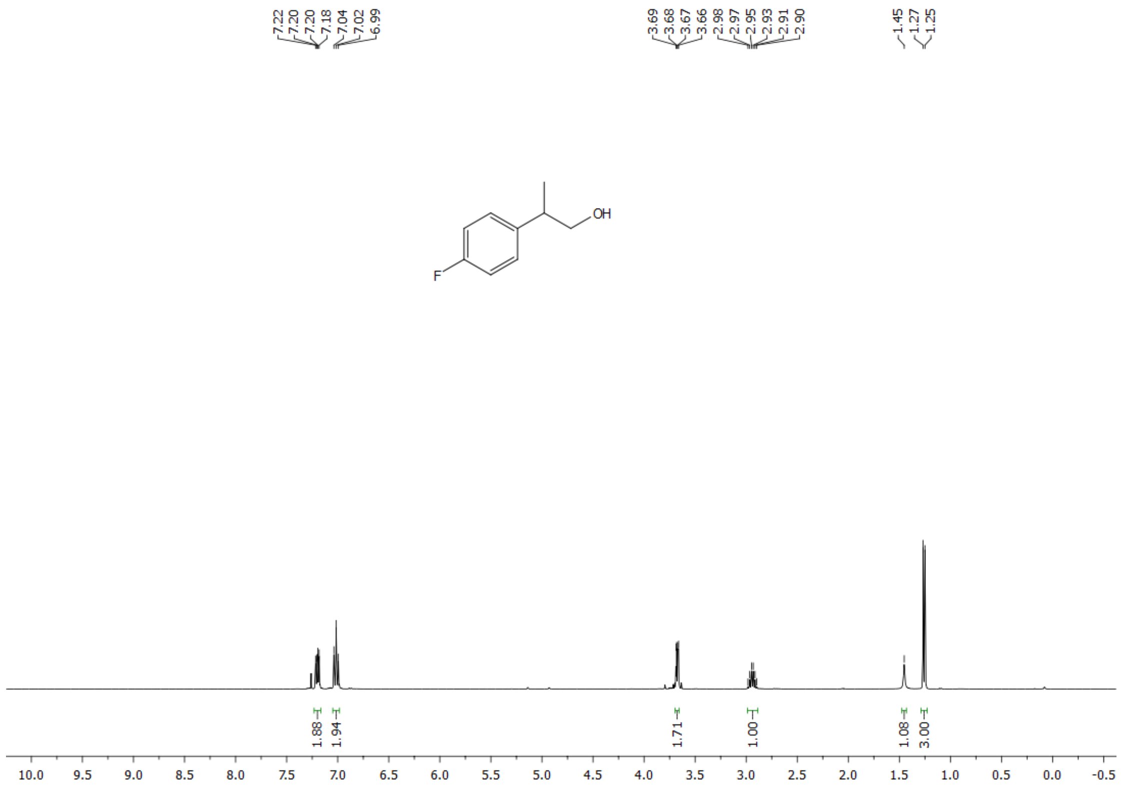

Figure S29: ^{1}H NMR (400 MHz, $CDCl_3$, 298 K) spectrum of 2-(4-fluorophenyl)propan-1-ol 3h.

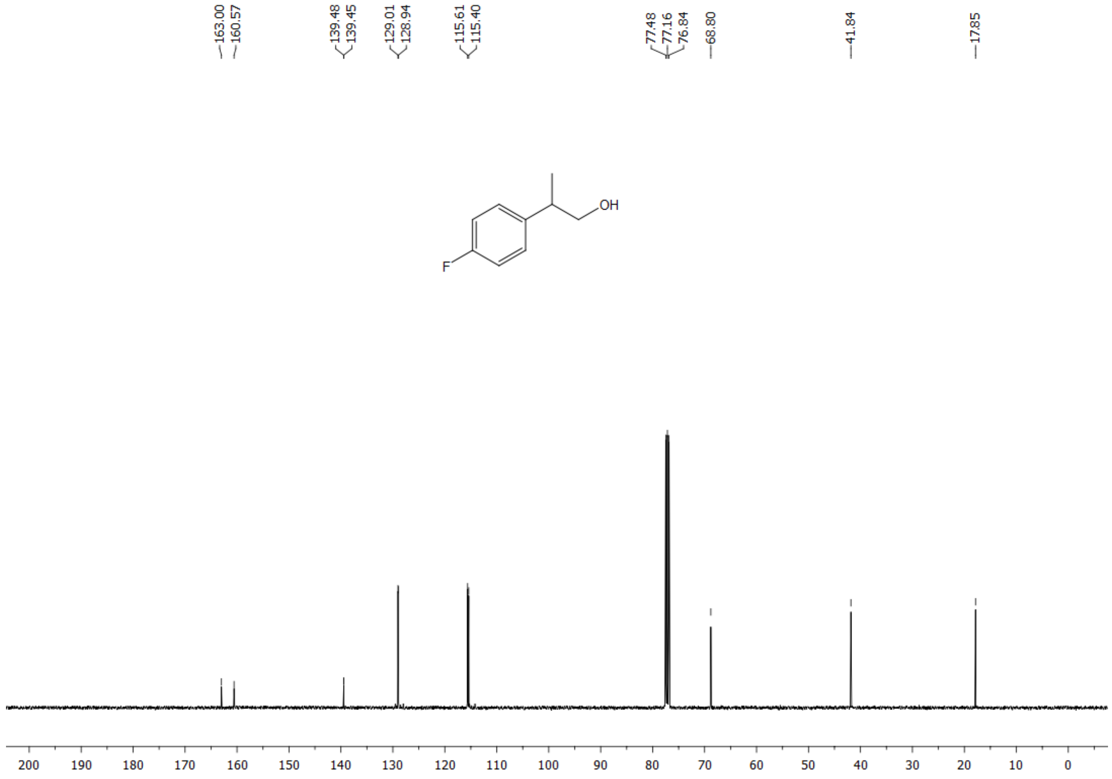

Figure S30: $^{13}C\{^{1}H\}$ NMR (101 MHz, $CDCl_3$, 298 K) spectrum of 2-(4-fluorophenyl)propan-1-ol 3h.

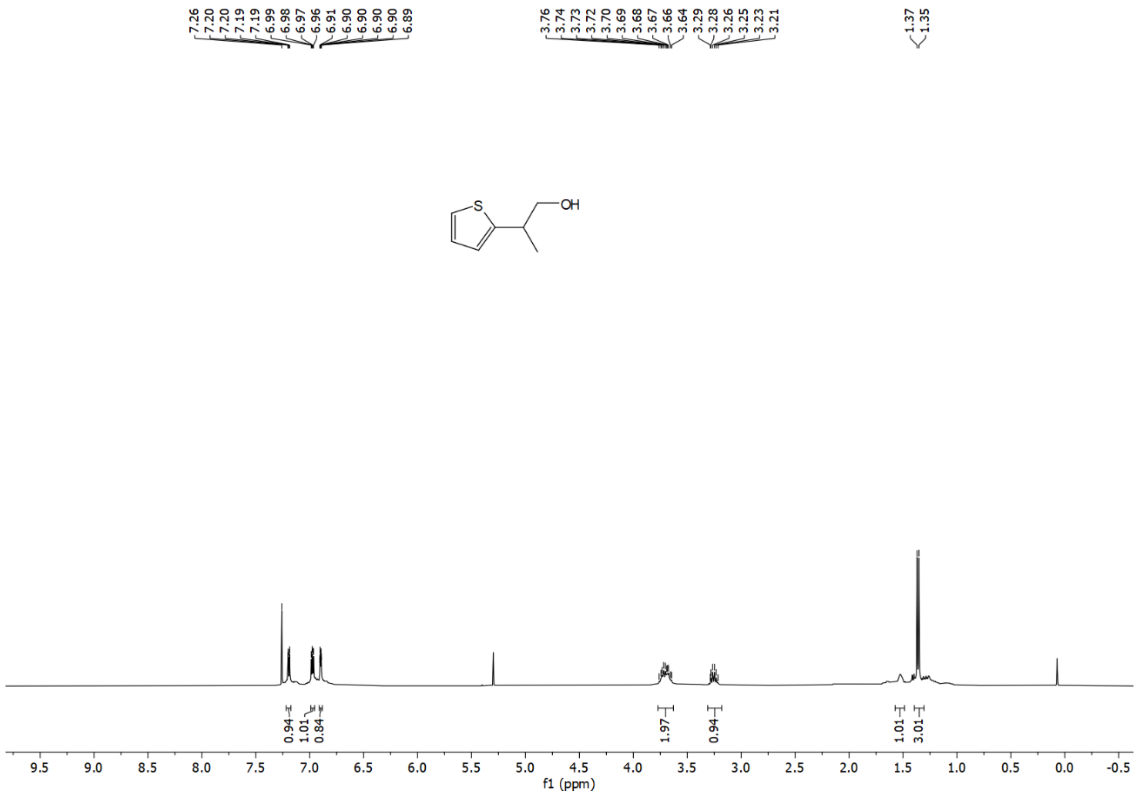

Figure S31: ^{1}**H NMR (400 MHz, CDCl$_3$, 298 K) spectrum of 2-(thiophen-2-yl)propan-1-ol 3i.**

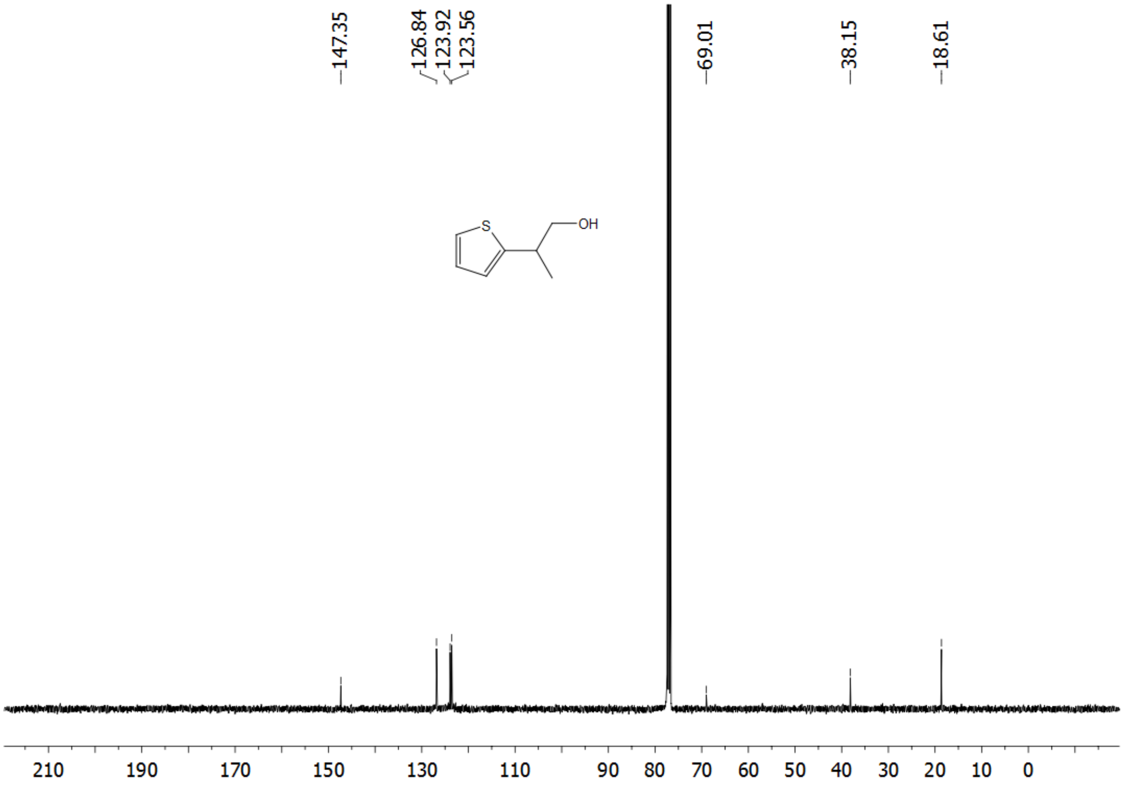

Figure S32: ^{13}**C{^{1}H} NMR (101 MHz, CDCl$_3$, 298 K) spectrum of 2-(thiophen-2-yl)propan-1-ol 3i.**

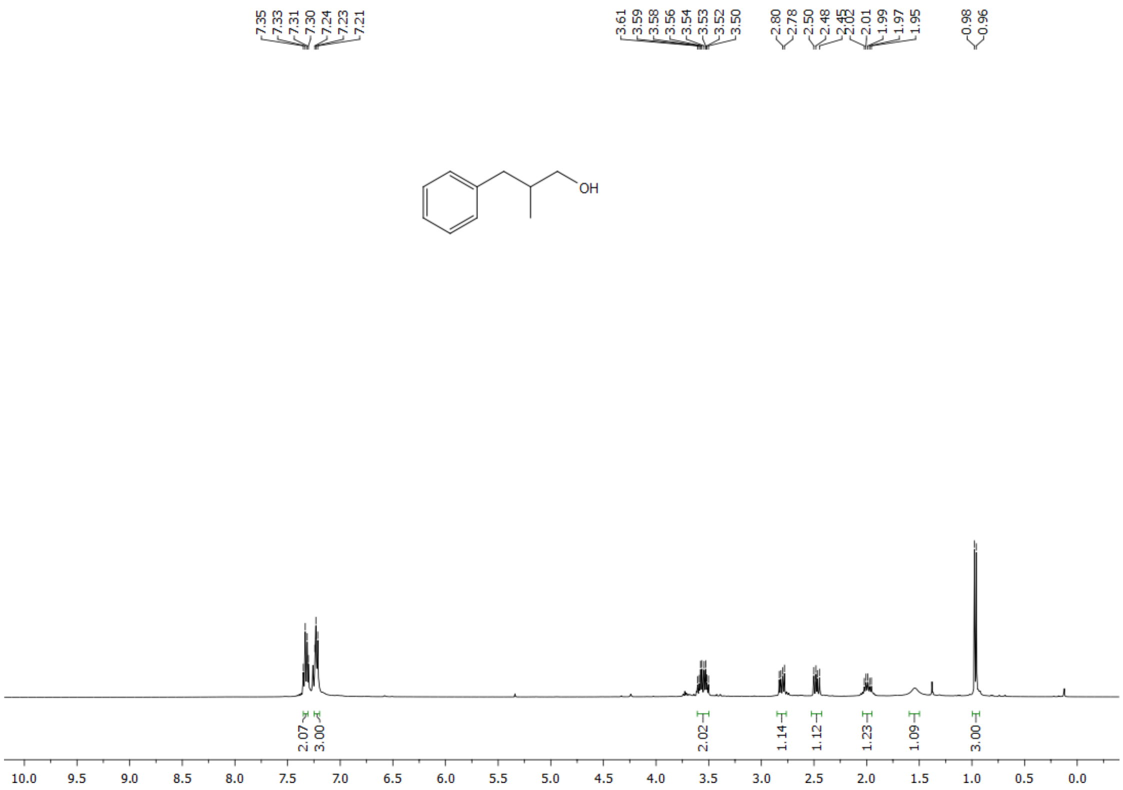

Figure S33: ^{1}H NMR (400 MHz, $CDCl_3$, 298 K) spectrum of 2-methyl-3-phenylpropan-1-ol 3j.

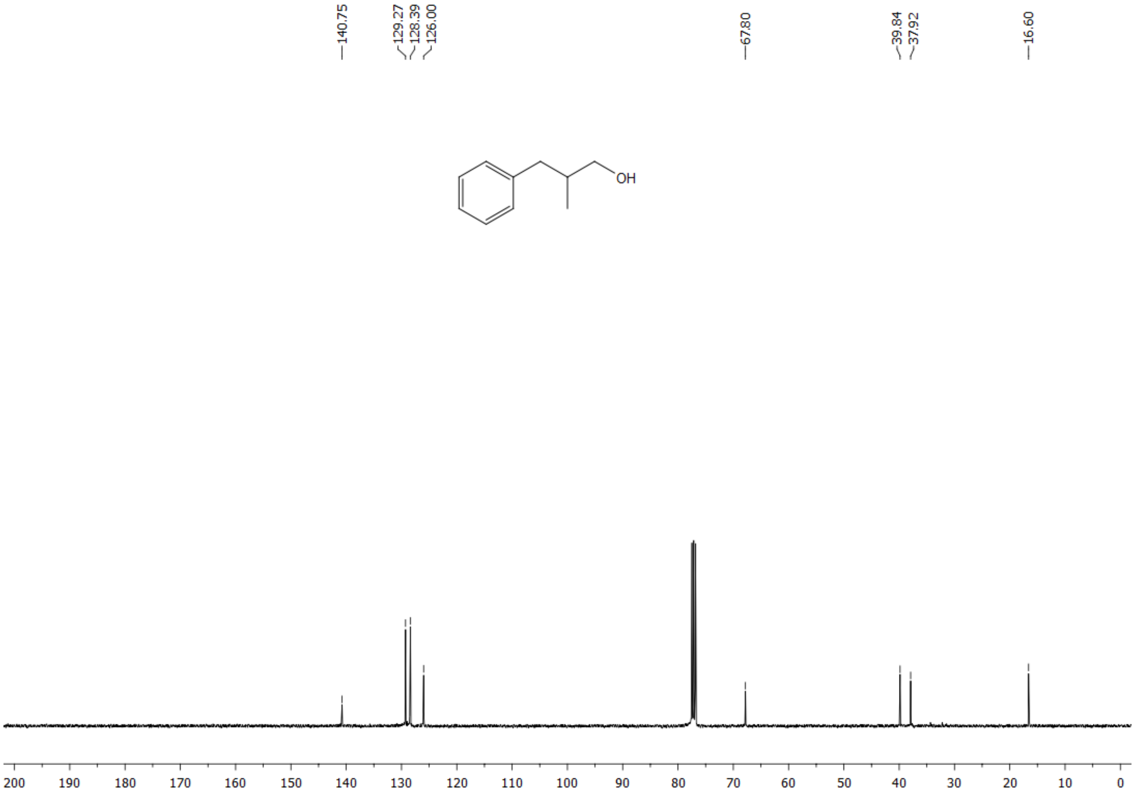

Figure S34:$^{13}C\{^1H\}$ NMR (101 MHz, $CDCl_3$, 298 K) spectrum of 2-methyl-3-phenylpropan-1-ol 3j.

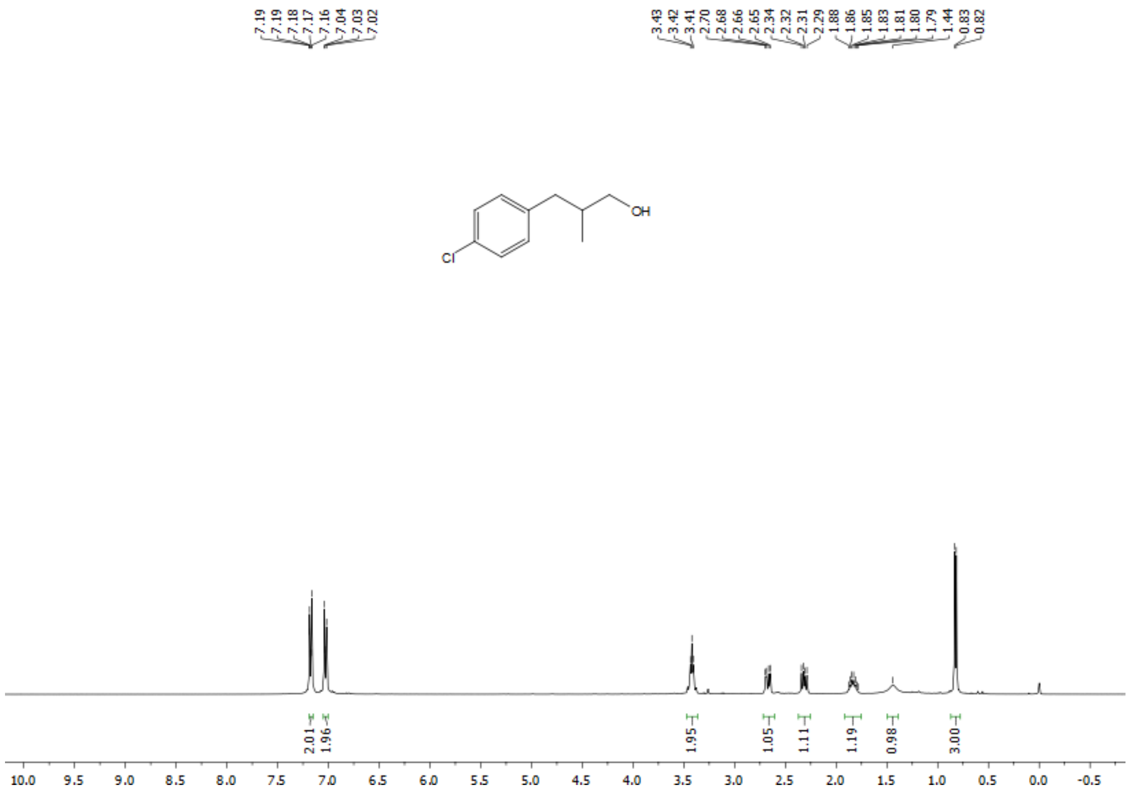

Figure S35: ^{1}H NMR (400 MHz, $CDCl_3$, 298 K) spectrum of 3-(4-chlorophenyl)-2-methylpropan-1-ol 3k.

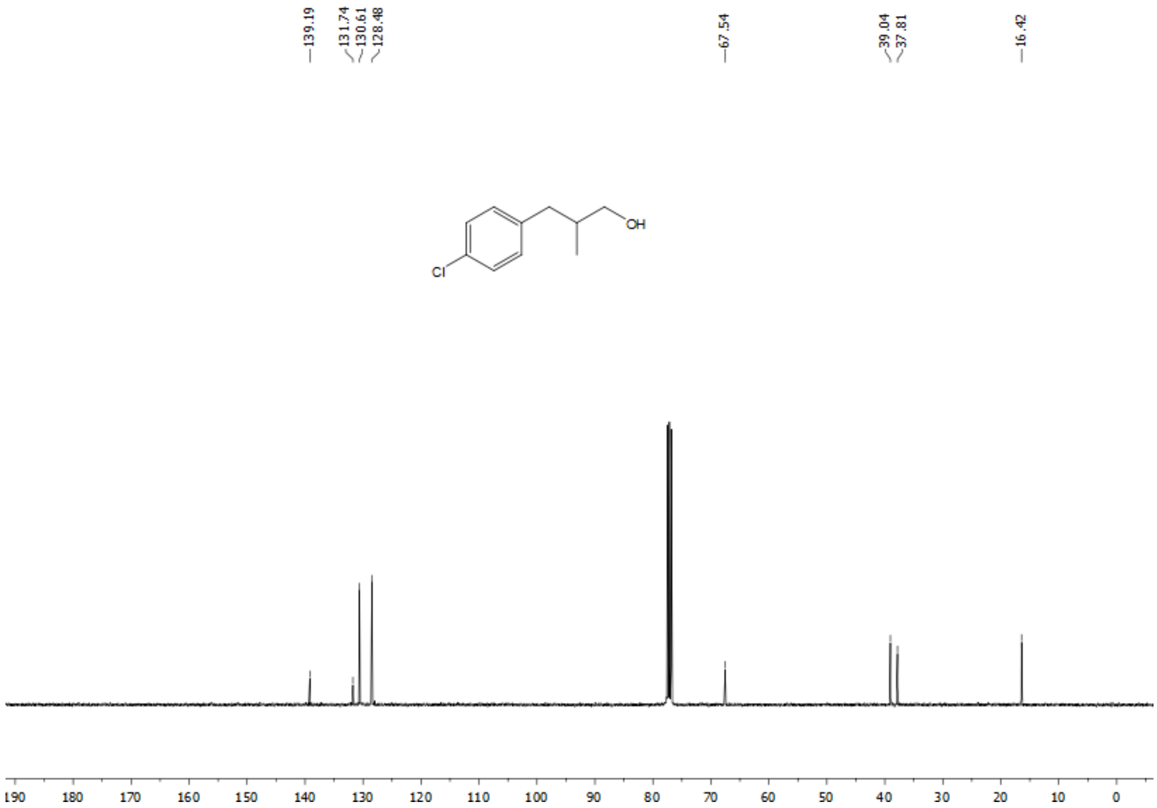

Figure S36: ^{13}C{^{1}H} NMR (101 MHz, $CDCl_3$, 298 K) spectrum of 3-(4-chlorophenyl)-2-methylpropan-1-ol 3k.

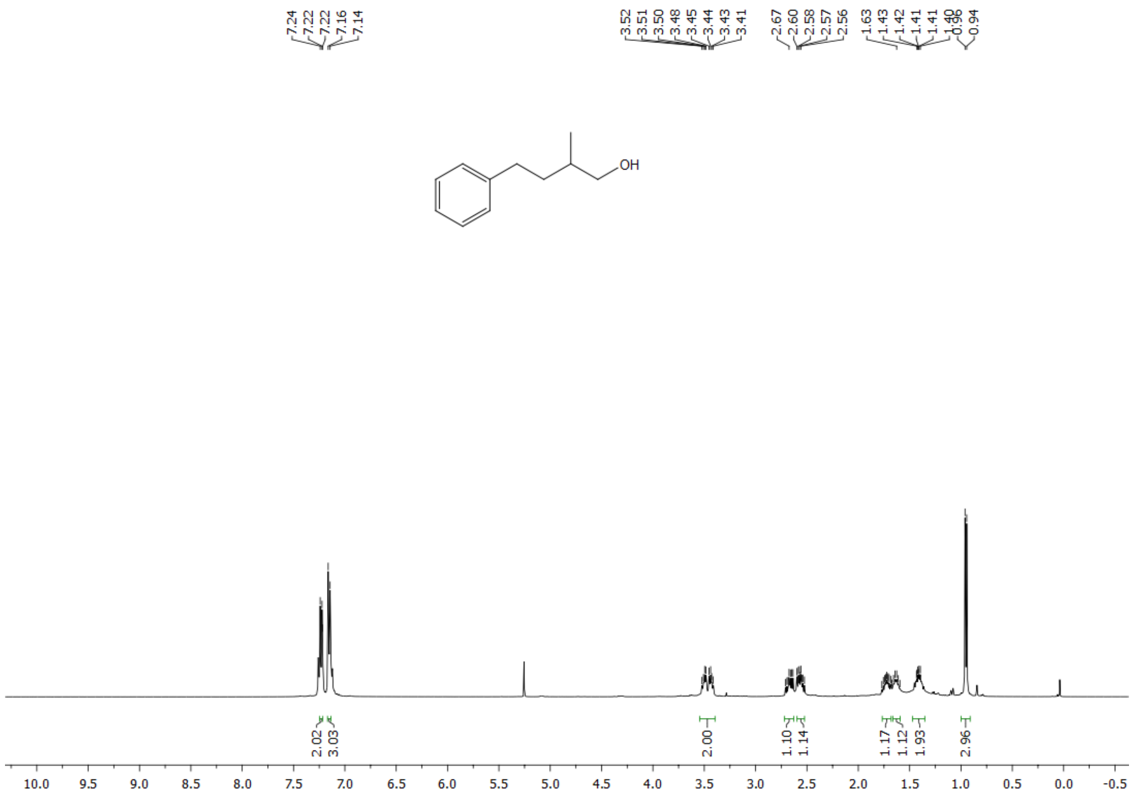

Figure S37: ^{1}H NMR (400 MHz, $CDCl_3$, 298 K) spectrum of 2-methyl-4-phenylbutan-1-ol 3l.

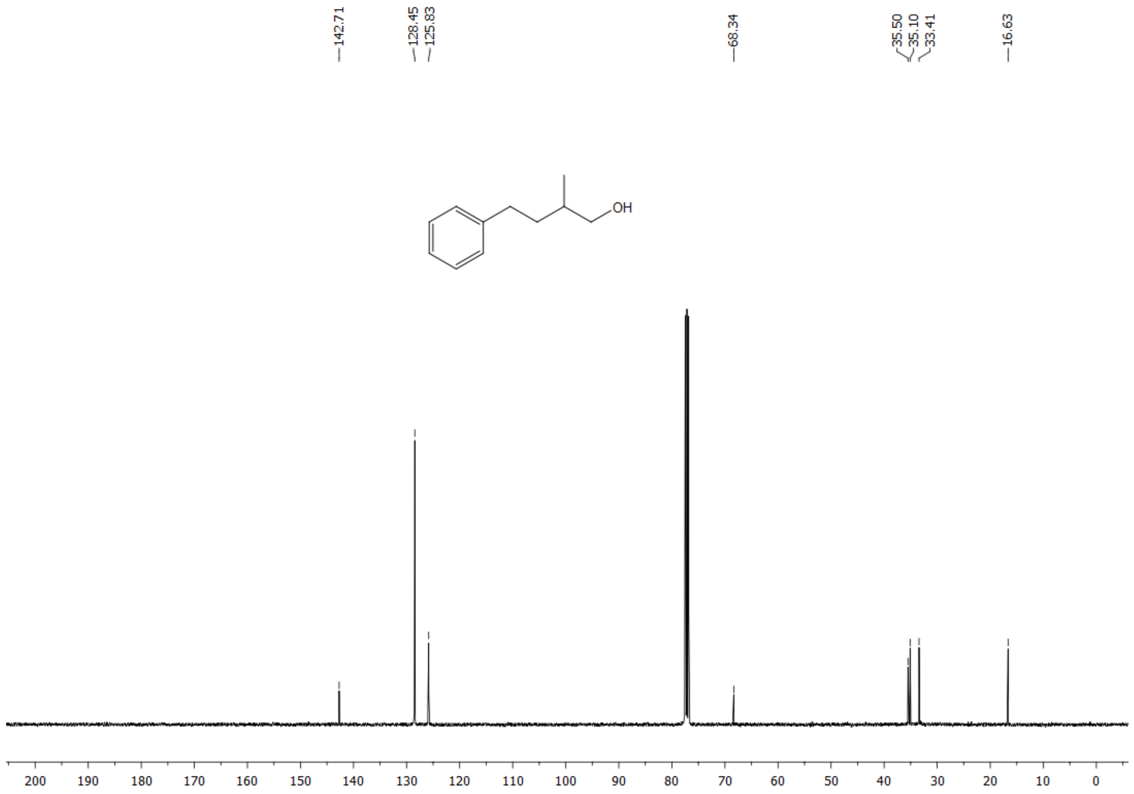

Figure S38: $^{13}C\{^{1}H\}$ NMR (101 MHz, $CDCl_3$, 298 K) spectrum of 2-methyl-4-phenylbutan-1-ol 3l.

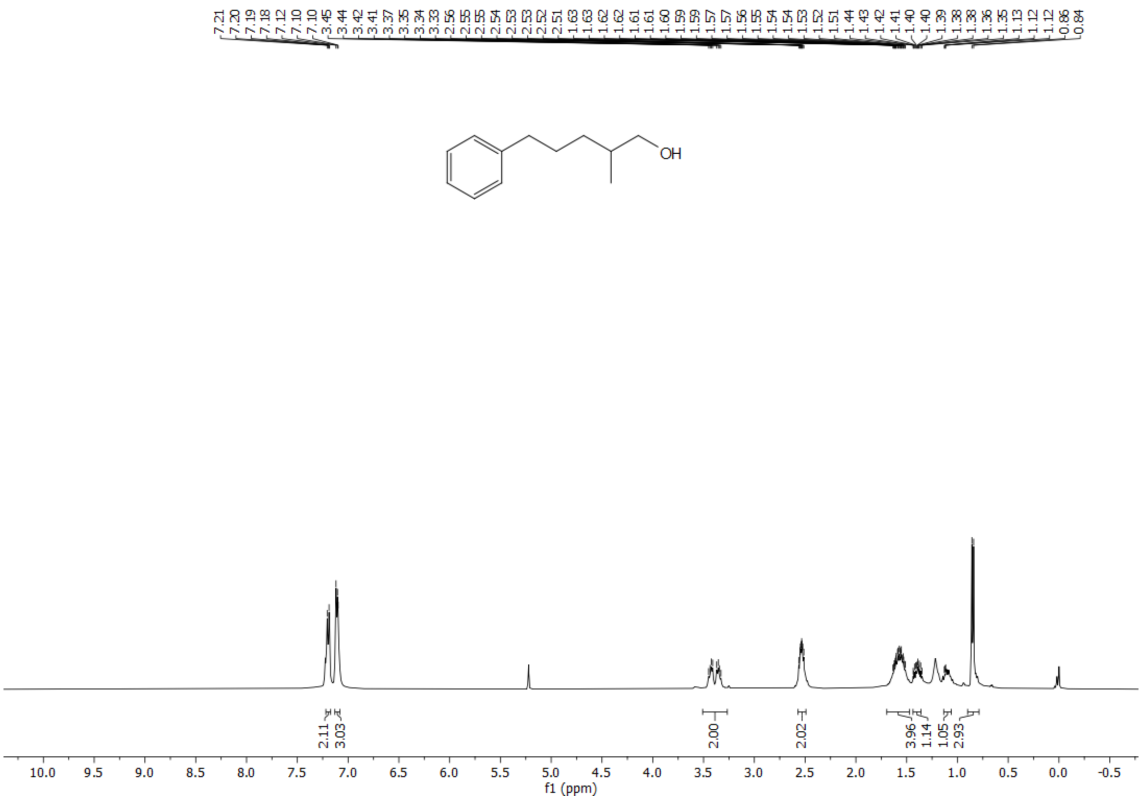

Figure S39:^{1}H NMR (400 MHz, $CDCl_3$, 298 K) spectrum of 2-methyl-5-phenylpentan-1-ol 3m.

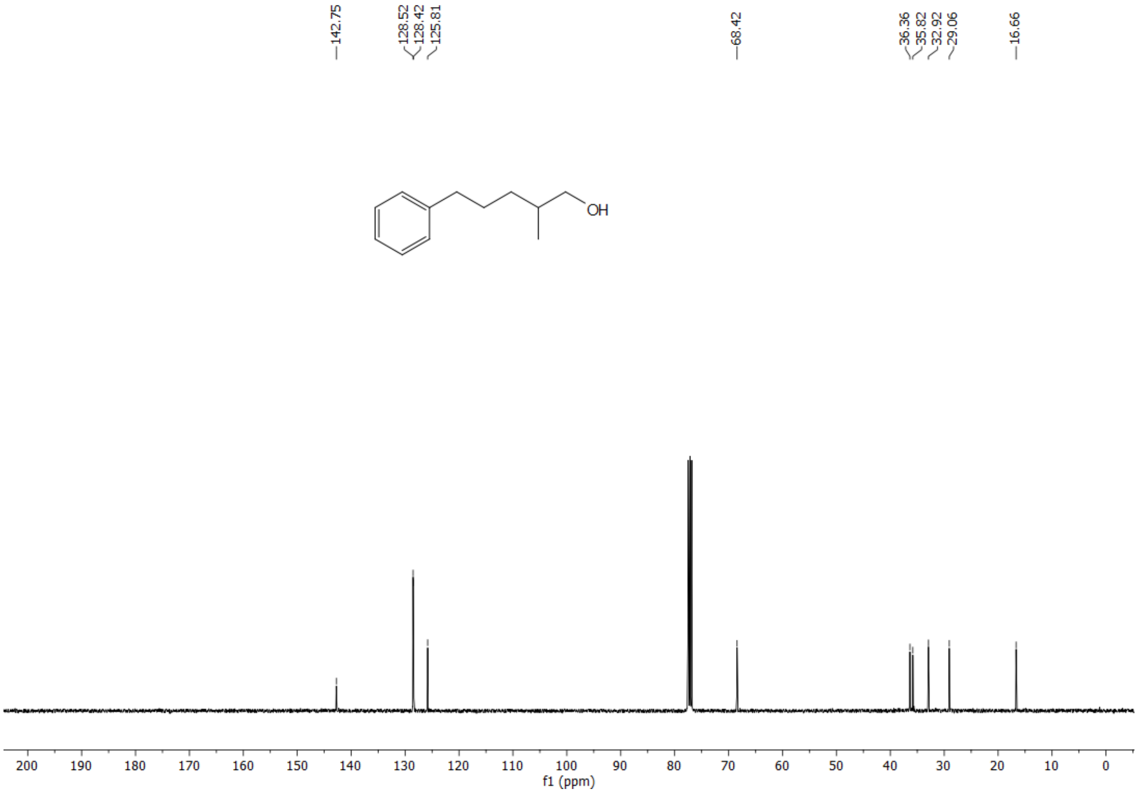

Figure S40:^{13}C{^{1}H} NMR (101 MHz, $CDCl_3$, 298 K) spectrum of 2-methyl-5-phenylpentan-1-ol 3m.

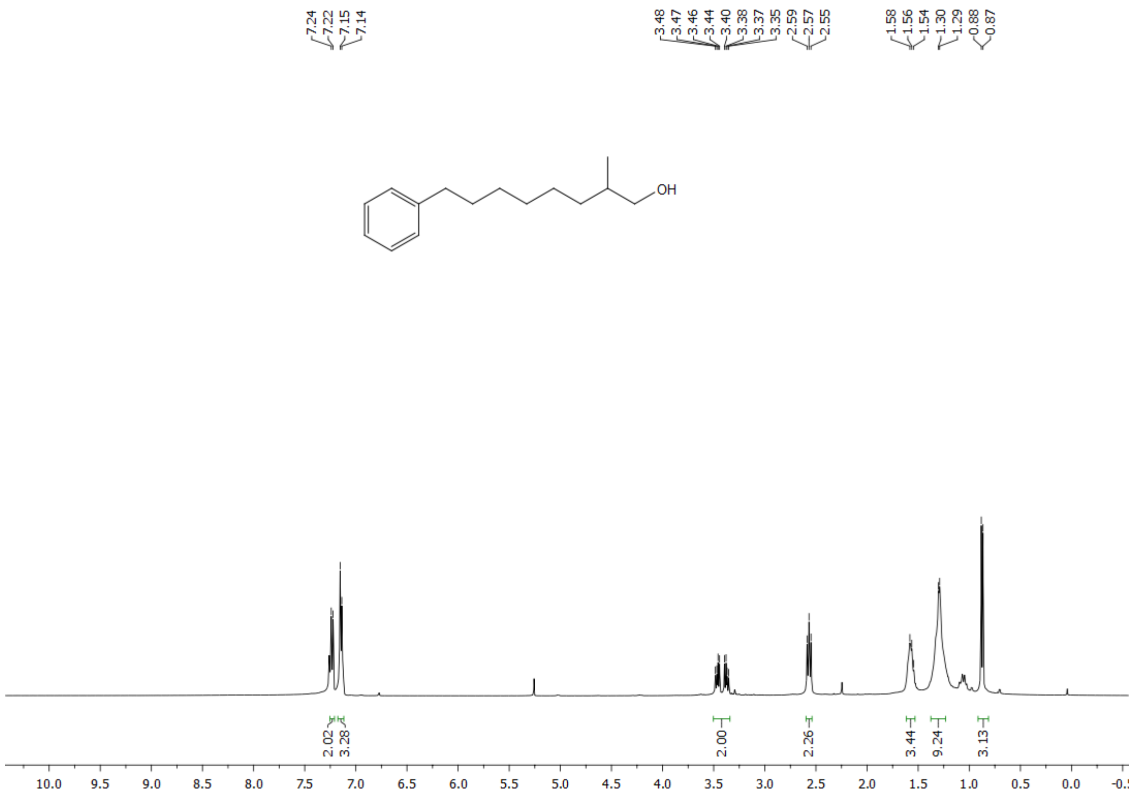

Figure S41:[1]**H NMR (400 MHz, $CDCl_3$, 298 K) spectrum of 2-methyl-8-phenyloctan-1-ol 3n.**

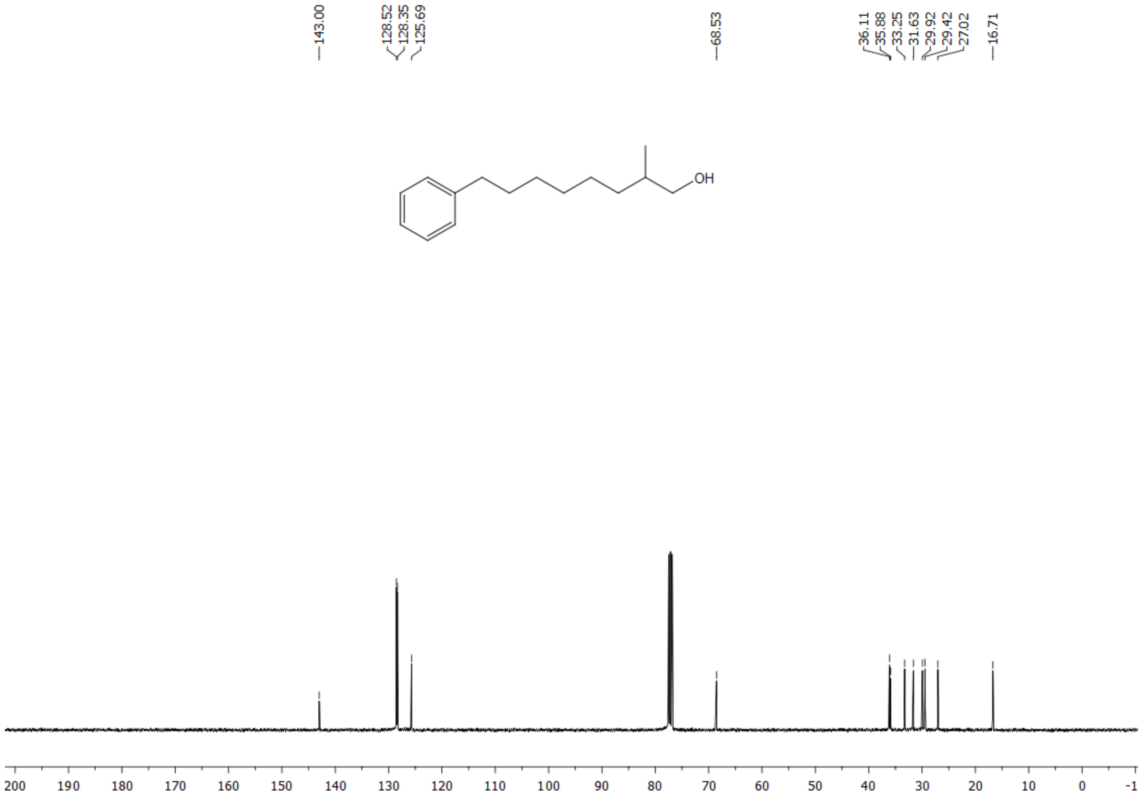

Figure S42: ^{13}**C{**^{1}**H} NMR (101 MHz, $CDCl_3$, 298 K) spectrum of 2-methyl-8-phenyloctan-1-ol 3n.**

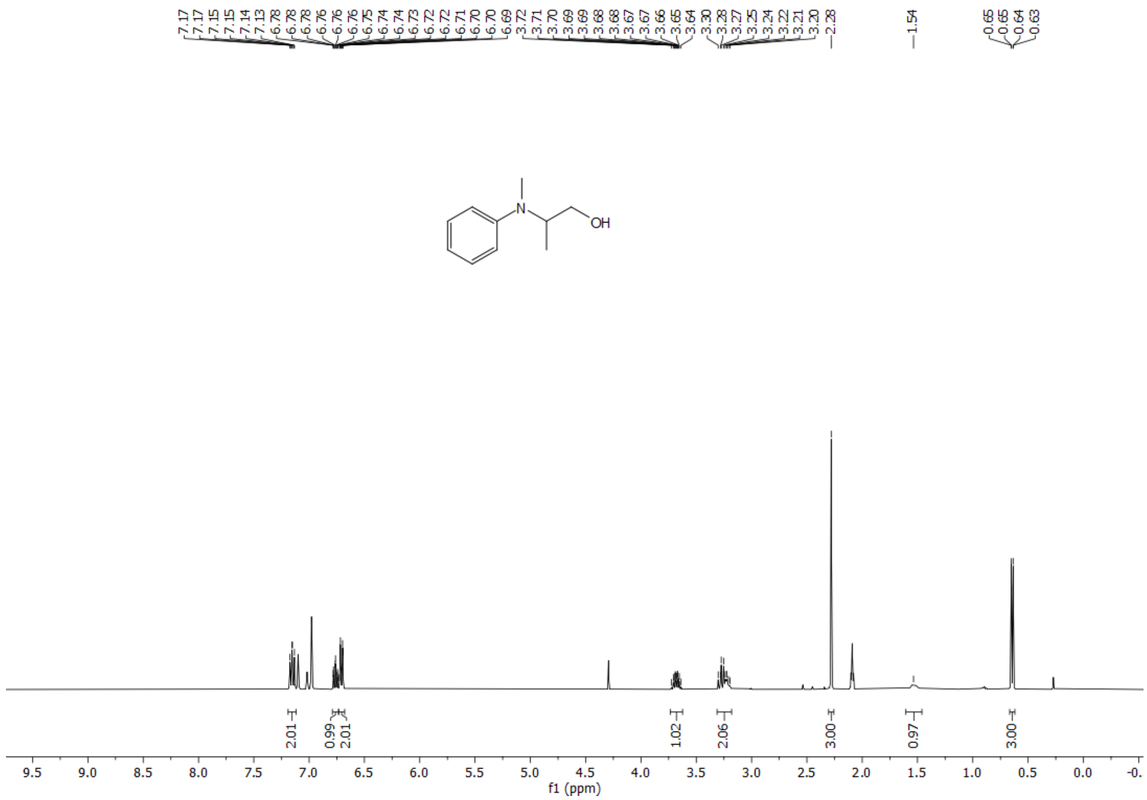

Figure S43: ^{1}H NMR (400 MHz, Tol-d8, 298 K) spectrum of 2-(methyl(phenyl)amino)propan-1-ol 3o.

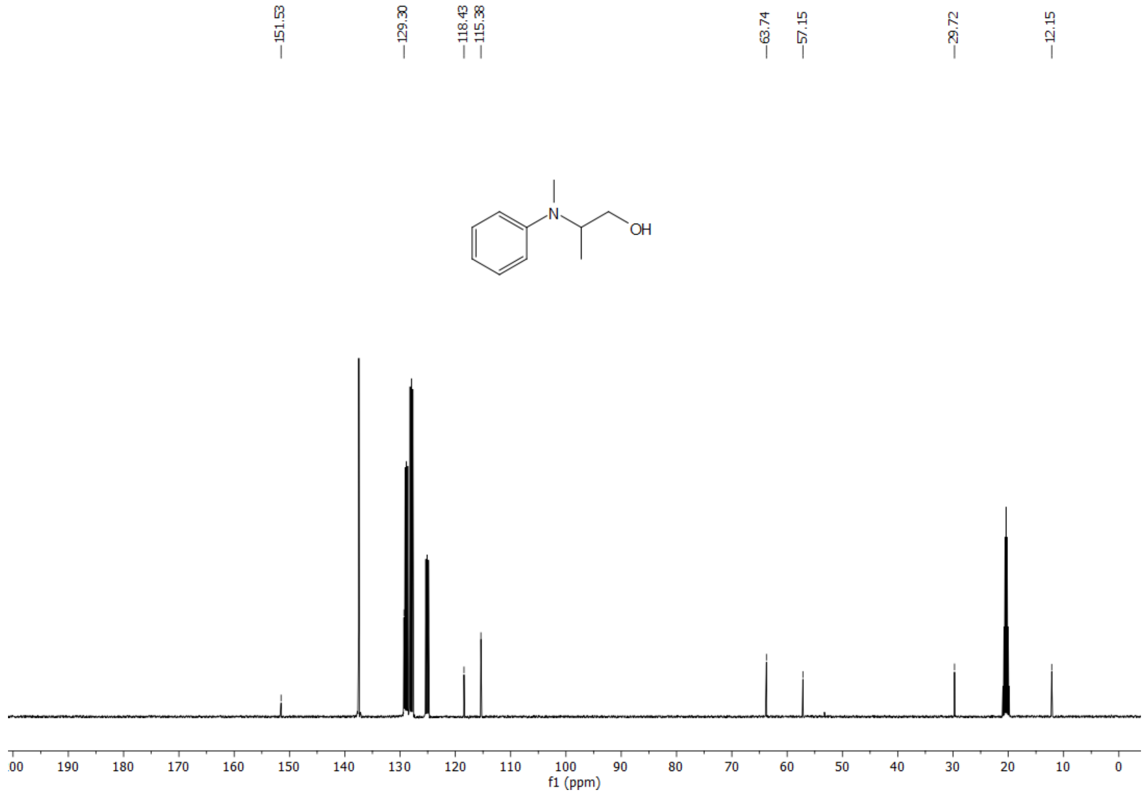

Figure S44: ^{13}C{^{1}H} NMR (101 MHz, Tol-d8, 298 K) spectrum of 2-(methyl(phenyl)amino)propan-1-ol 3o.

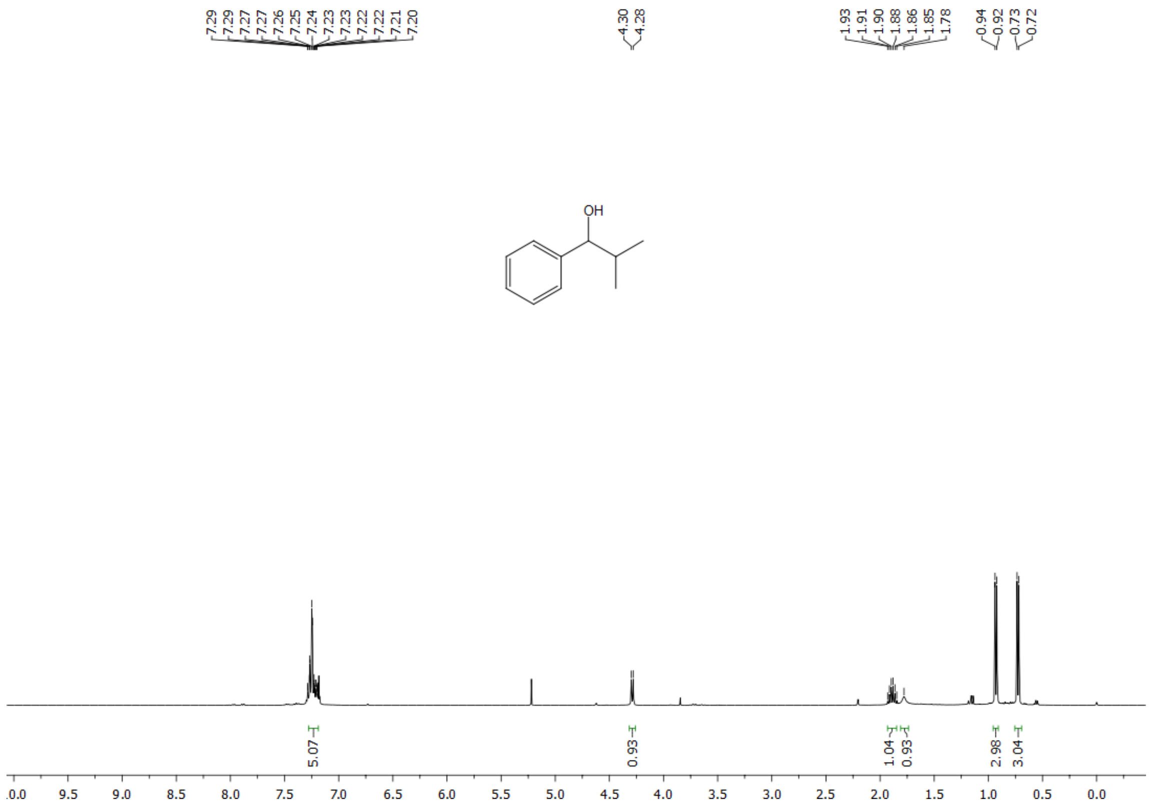

Figure S45: ^{1}H NMR (400 MHz, $CDCl_3$, 298 K) spectrum of 2-methyl-1-phenylpropan-1-ol 8a.

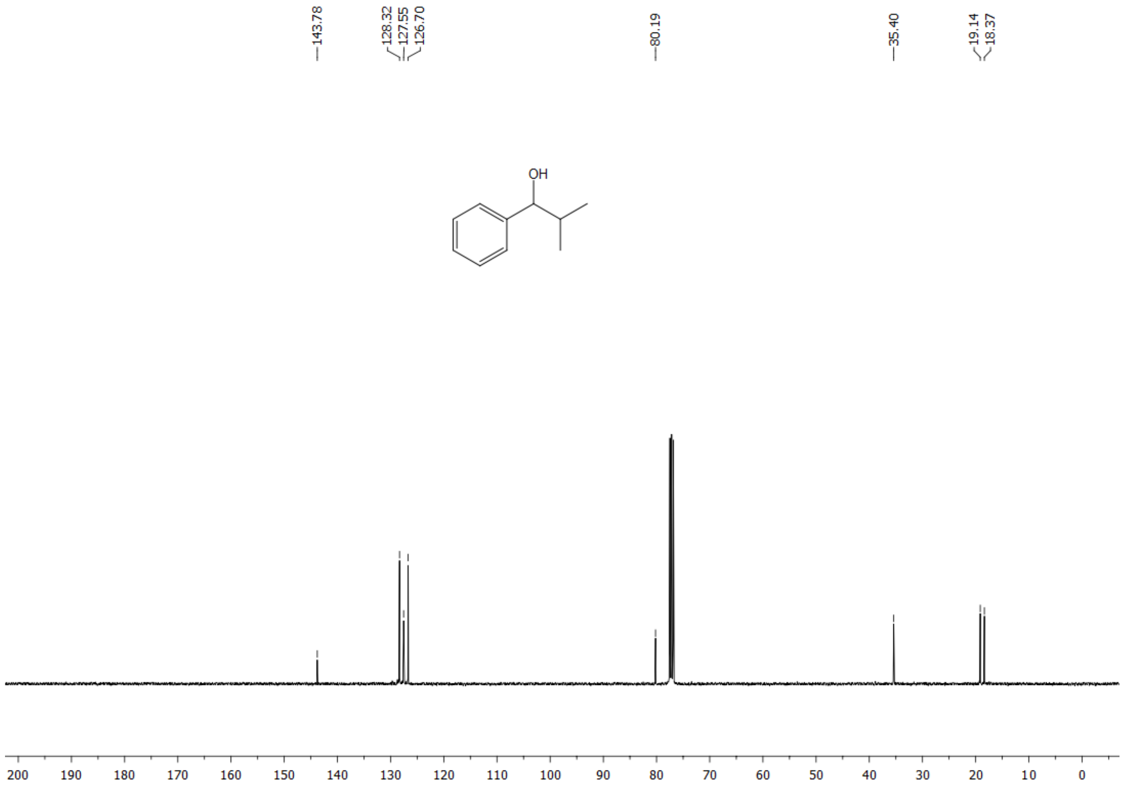

Figure S46: $^{13}C\{^{1}H\}$ NMR (101 MHz, $CDCl_3$, 298 K) spectrum of 2-methyl-1-phenylpropan-1-ol 8a.

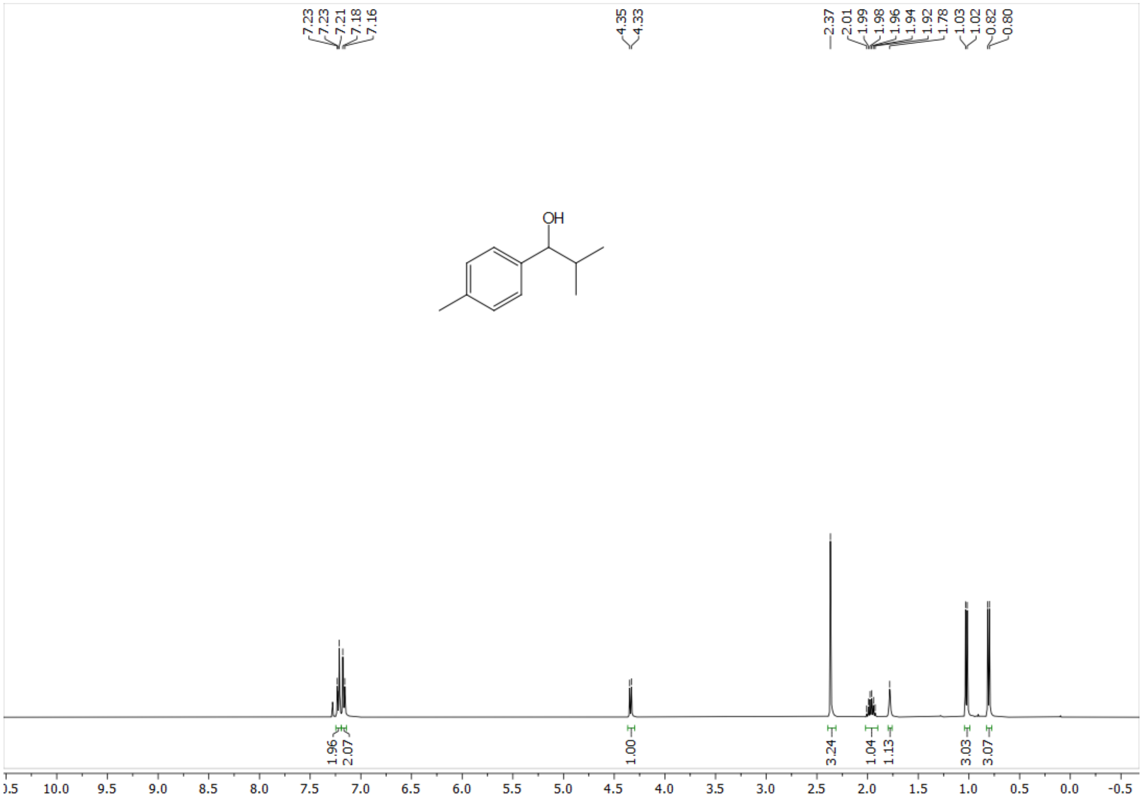

Figure S47: ^{1}H NMR (400 MHz, $CDCl_3$, 298 K) spectrum of 2-methyl-1-(p-tolyl)propan-1-ol 8b.

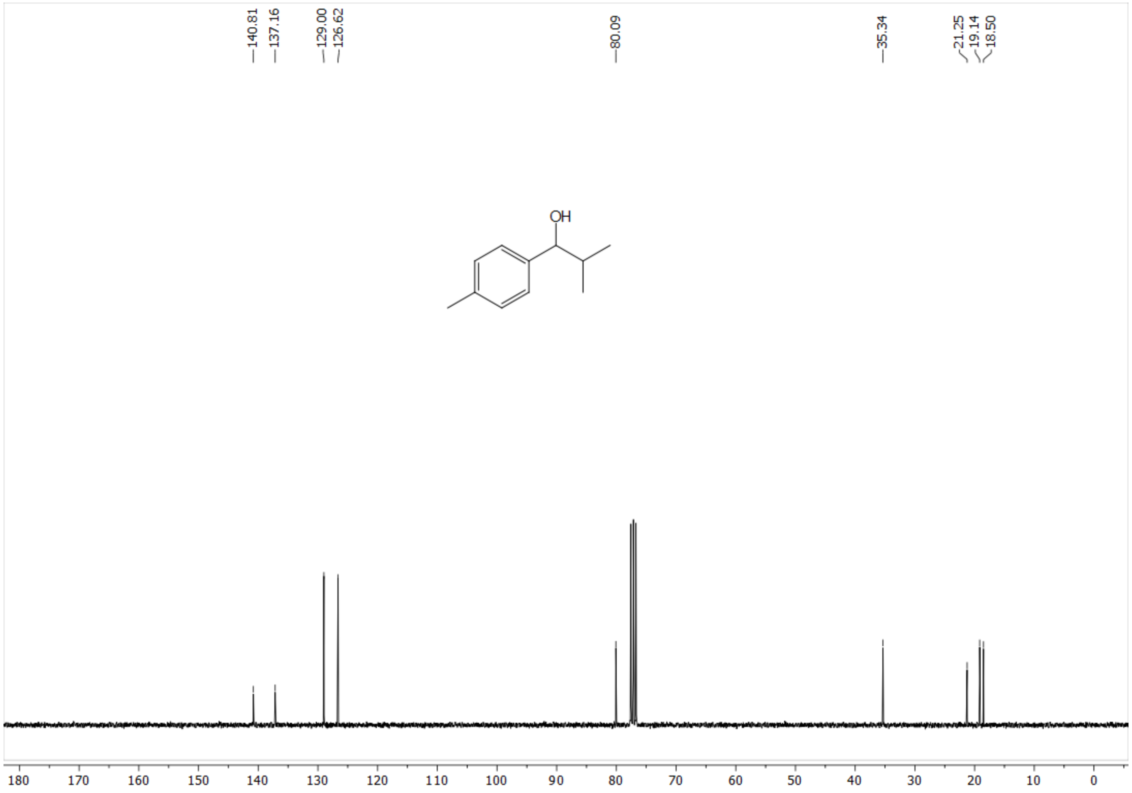

Figure S48: $^{13}C\{^{1}H\}$ NMR (101 MHz, $CDCl_3$, 298 K) spectrum of 2-methyl-1-(p-tolyl)propan-1-ol 8b.

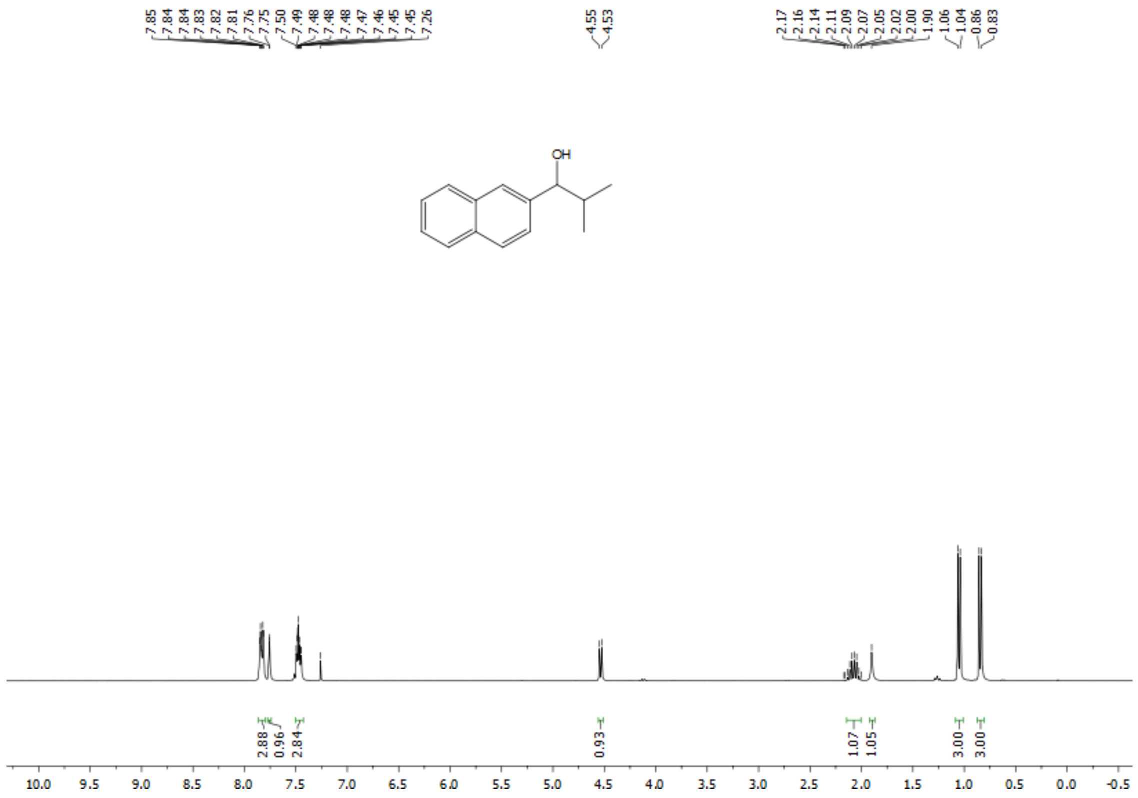

Figure S49: ^{1}H NMR (400 MHz, $CDCl_3$, 298 K) spectrum of 2-methyl-1-(naphthalen-2-yl)propan-1-ol 8d.

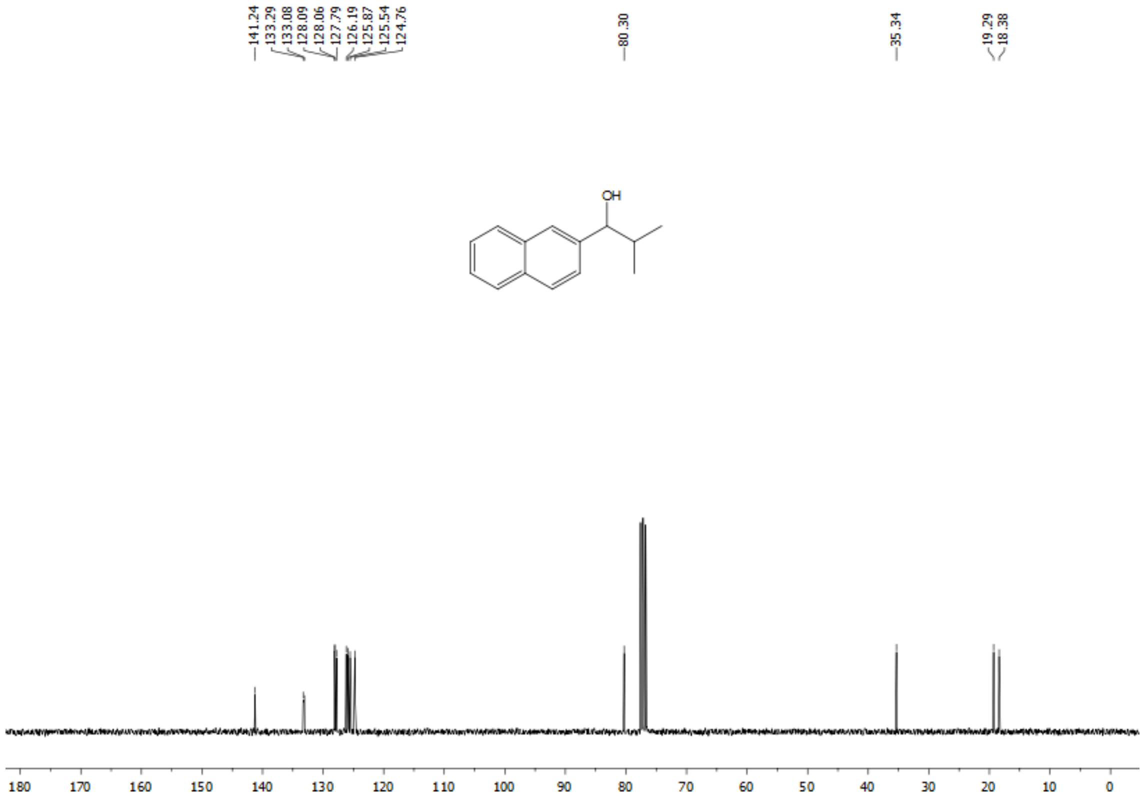

Figure S50: $^{13}C\{^{1}H\}$ NMR (101 MHz, $CDCl_3$, 298 K) spectrum of 2-methyl-1-(naphthalen-2-yl)propan-1-ol 8d.

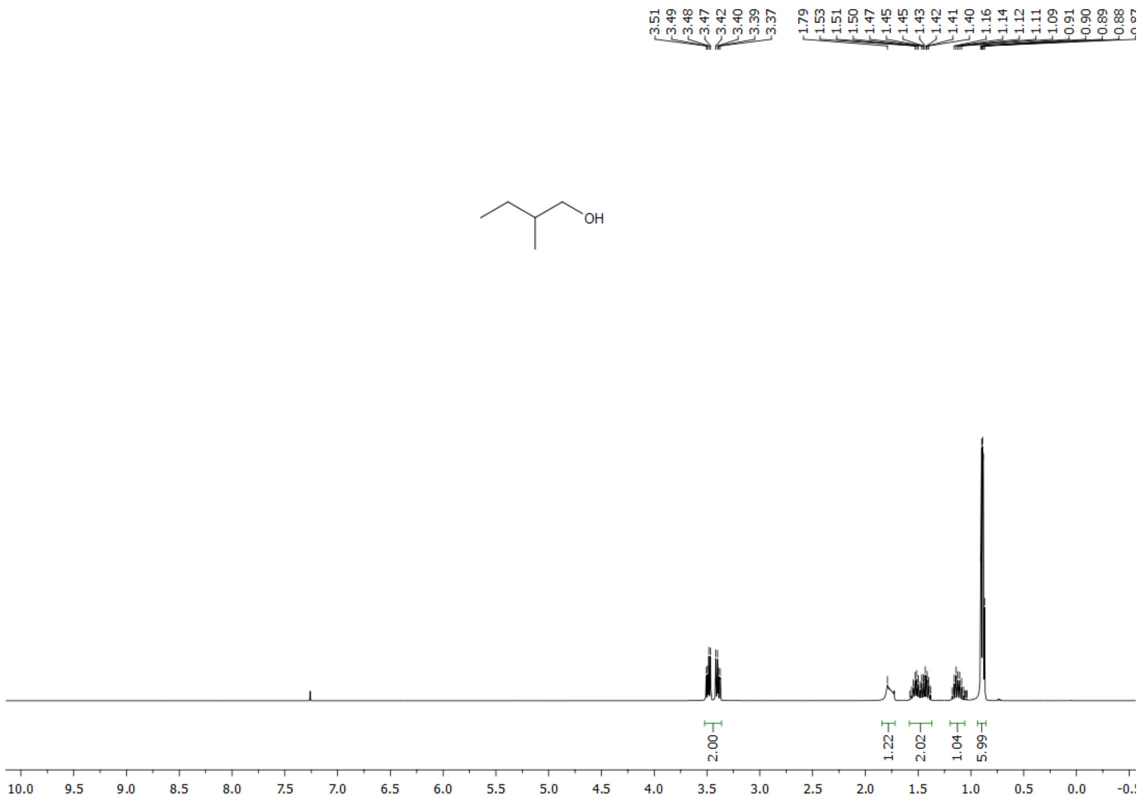

Figure S51: ^{1}H NMR (400 MHz, $CDCl_3$, 298 K) spectrum of 2-methylbutan-1-ol 10b.

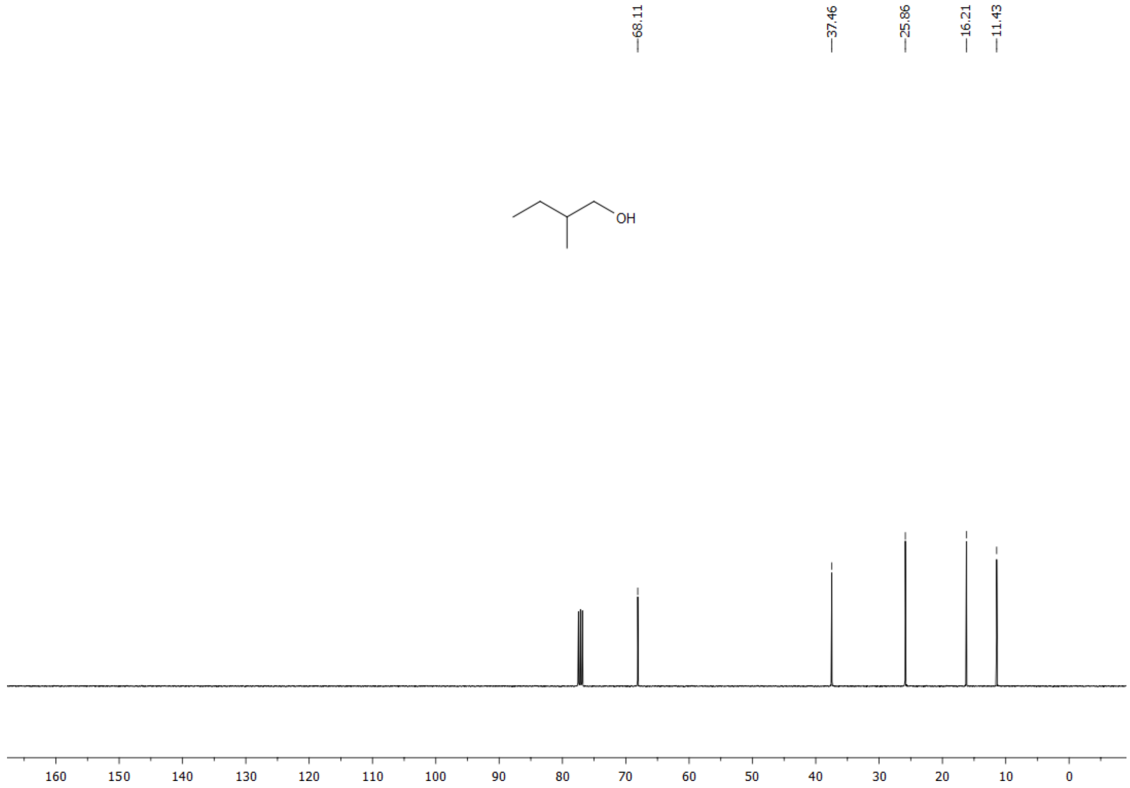

Figure S52: $^{13}C\{^{1}H\}$ NMR (101 MHz, $CDCl_3$, 298 K) spectrum of 2-methylbutan-1-ol 10b.

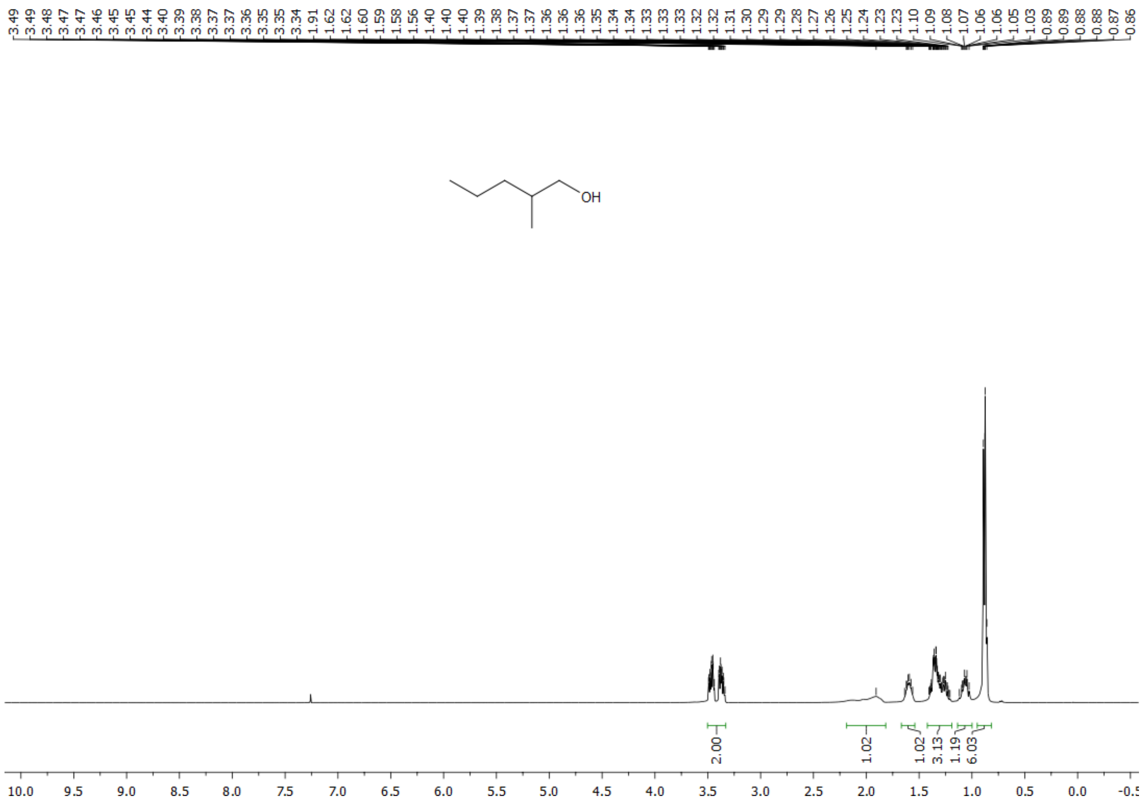

Figure S53: ^{1}H NMR (400 MHz, $CDCl_3$, 298 K) spectrum of 2-methylpentan-1-ol 10c.

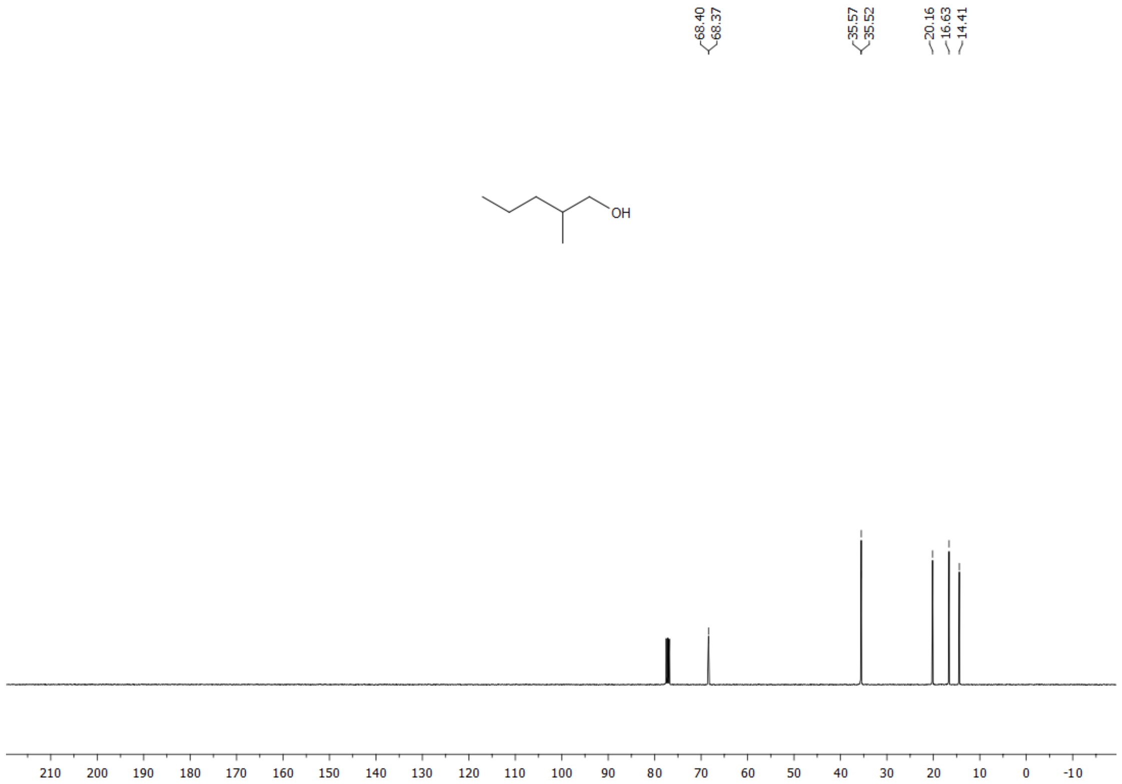

Figure S54: $^{13}C\{^{1}H\}$ NMR (101 MHz, $CDCl_3$, 298 K) spectrum of 2-methylpentan-1-ol 10c.

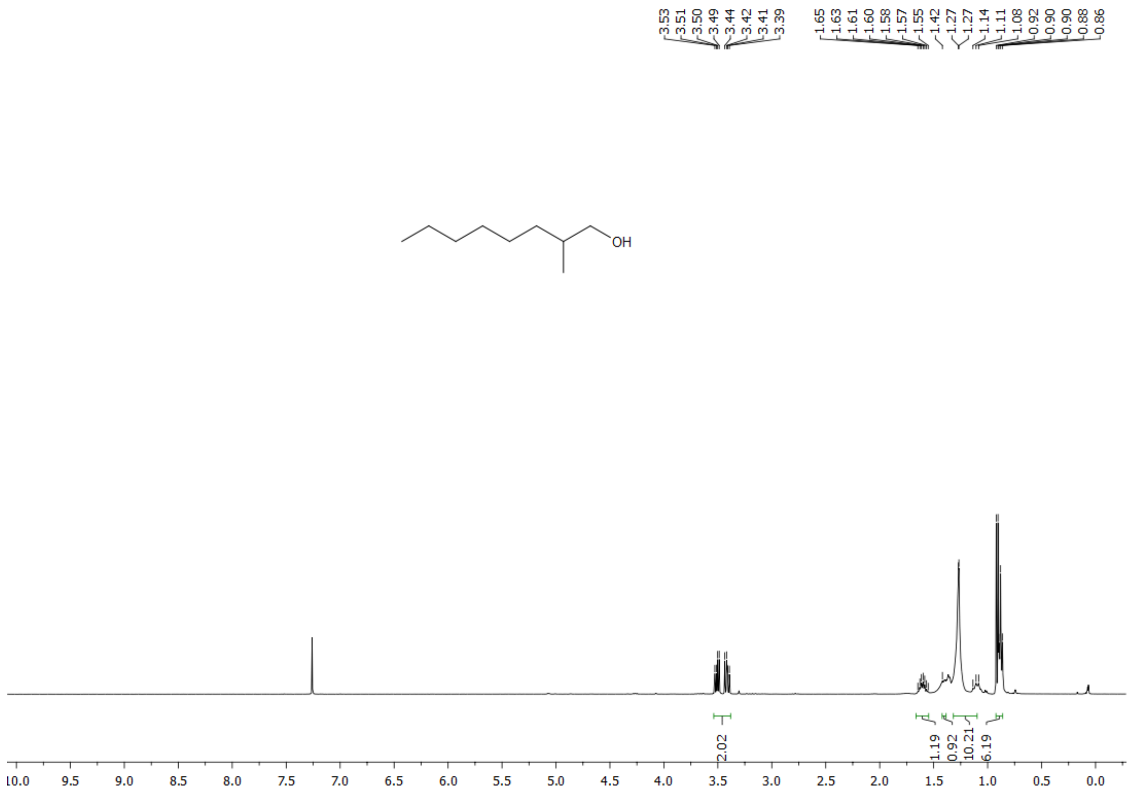

Figure S55: ^{1}H NMR (400 MHz, $CDCl_3$, 298 K) spectrum of 2-methyloctan-1-ol 10e.

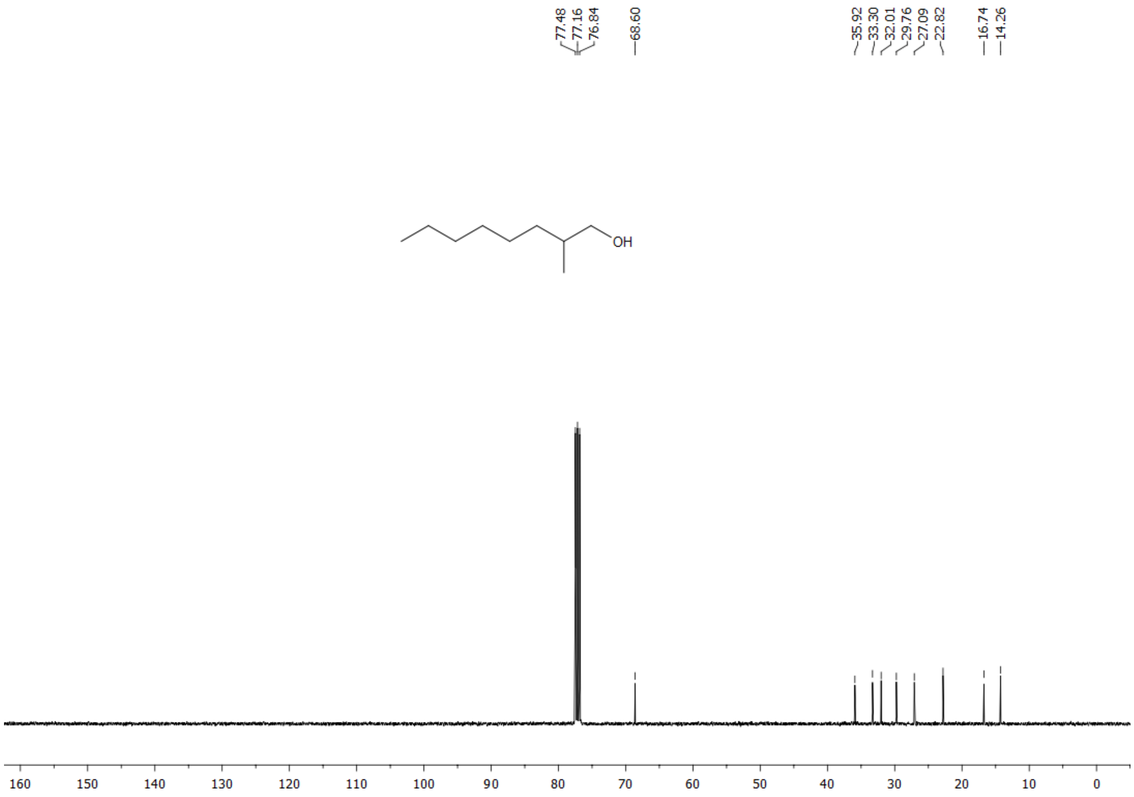

Figure S56: $^{13}C\{^{1}H\}$ NMR (101 MHz, $CDCl_3$, 298 K) spectrum of 2-methyloctan-1-ol 10e.

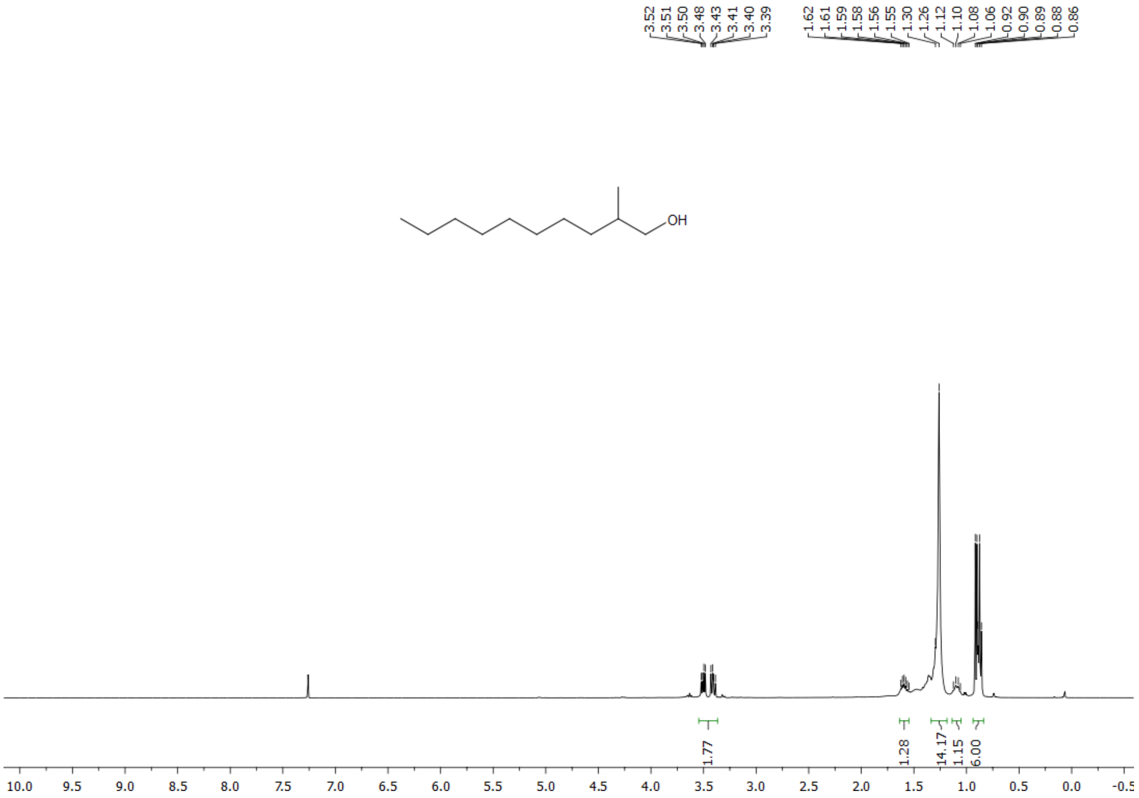

Figure S57: ^{1}H NMR (400 MHz, $CDCl_3$, 298 K) spectrum of 2-methyldecan-1-ol 10f.

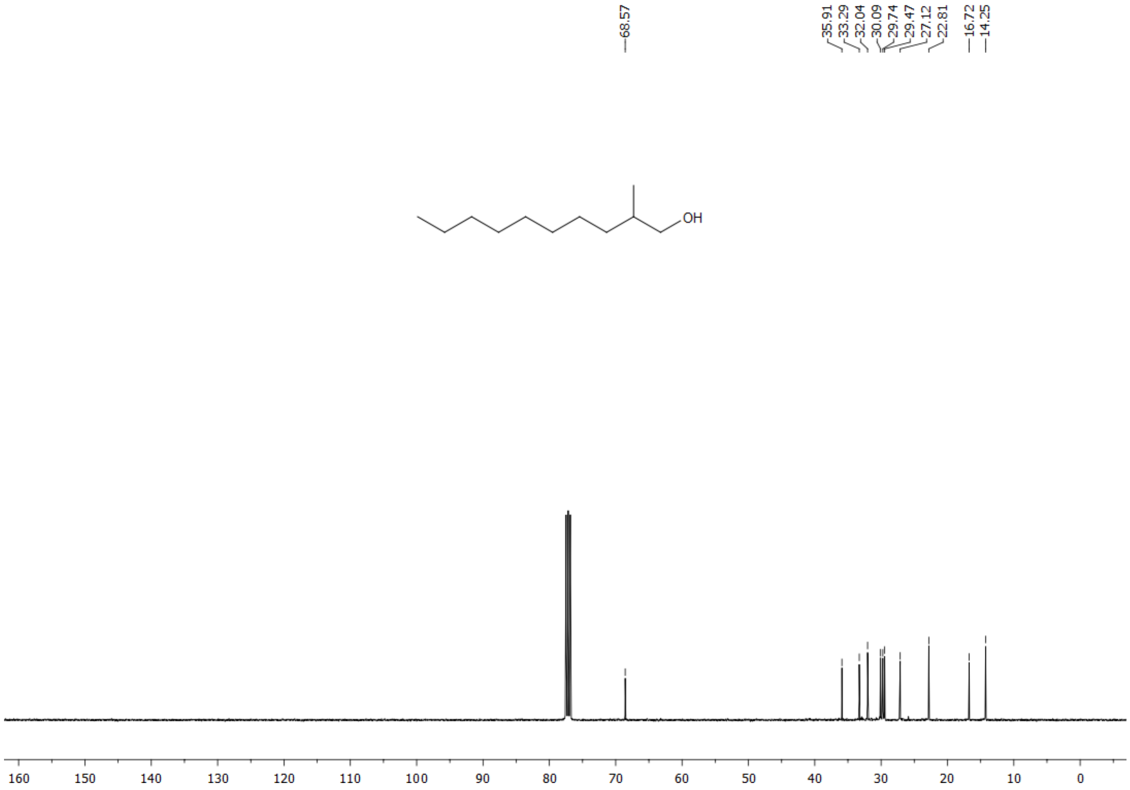

Figure S58: $^{13}C\{^{1}H\}$ NMR (101 MHz, $CDCl_3$, 298 K) spectrum of 2-methyldecan-1-ol 10f.

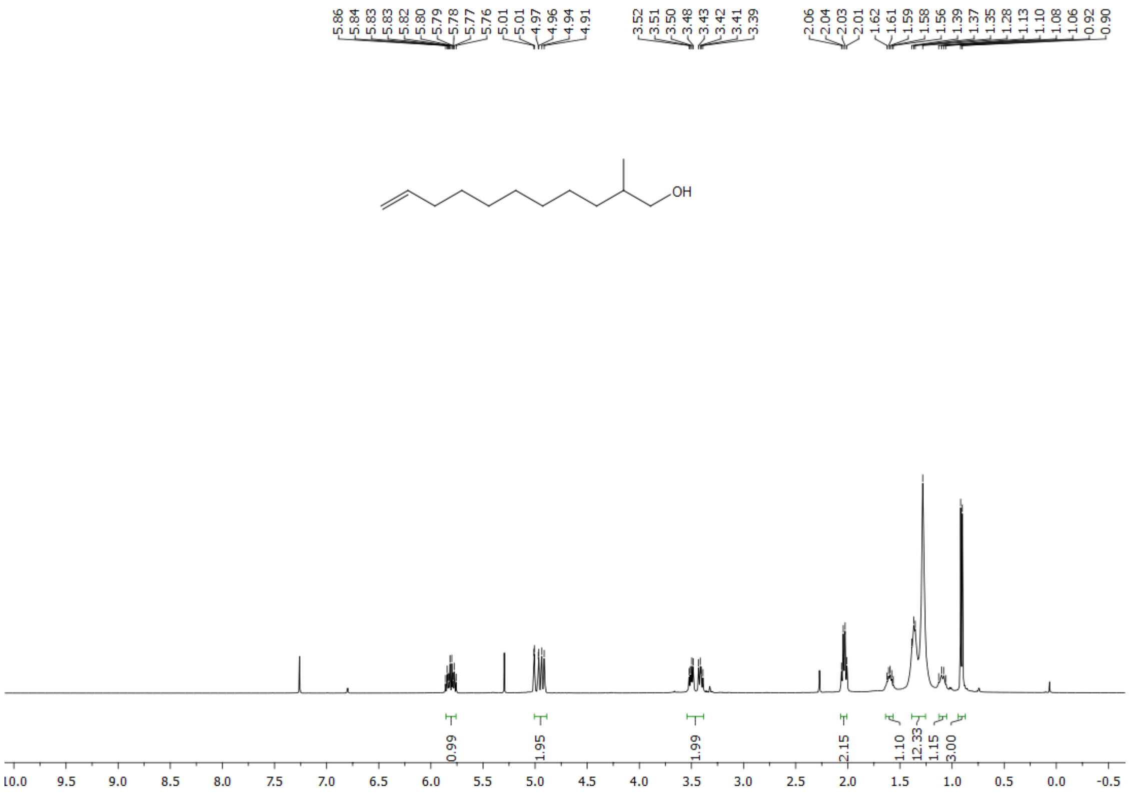

Figure S59: ^{1}H NMR (400 MHz, $CDCl_3$, 298 K) spectrum of 2-methylundec-10-en-1-ol 10g.

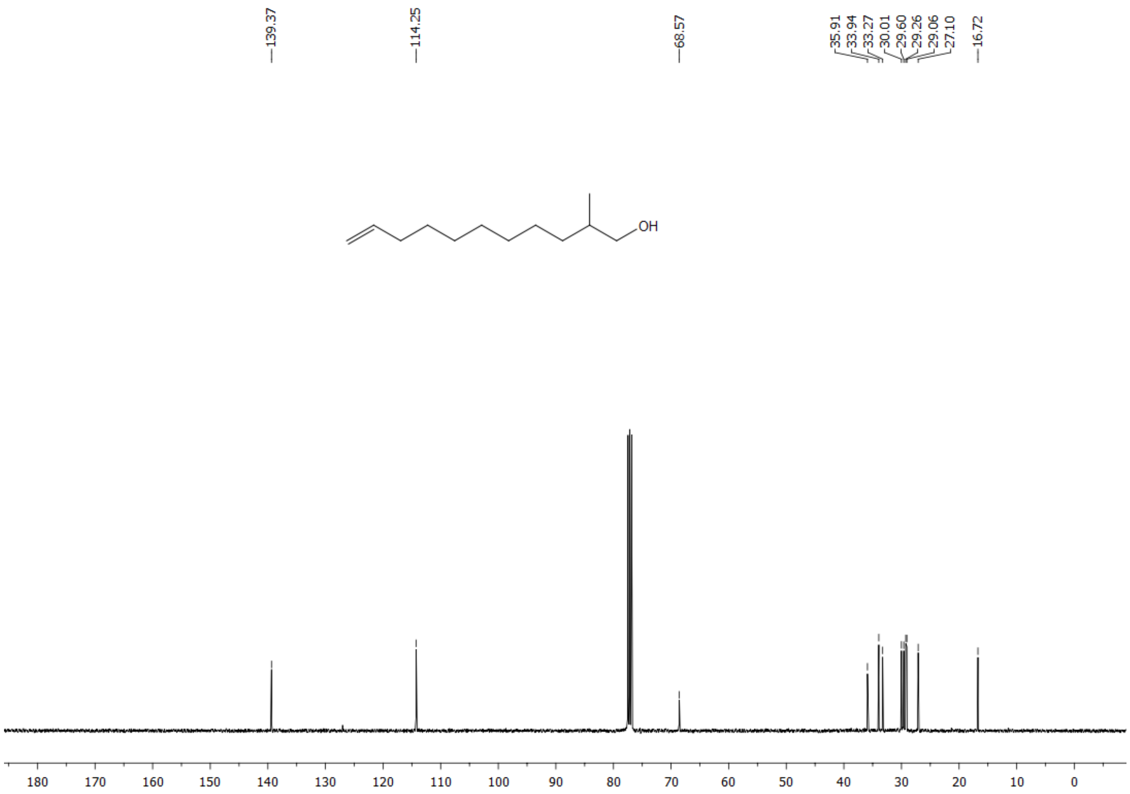

Figure S60: $^{13}C\{^{1}H\}$ NMR (101 MHz, $CDCl_3$, 298 K) spectrum of 2-methylundec-10-en-1-ol 10g.

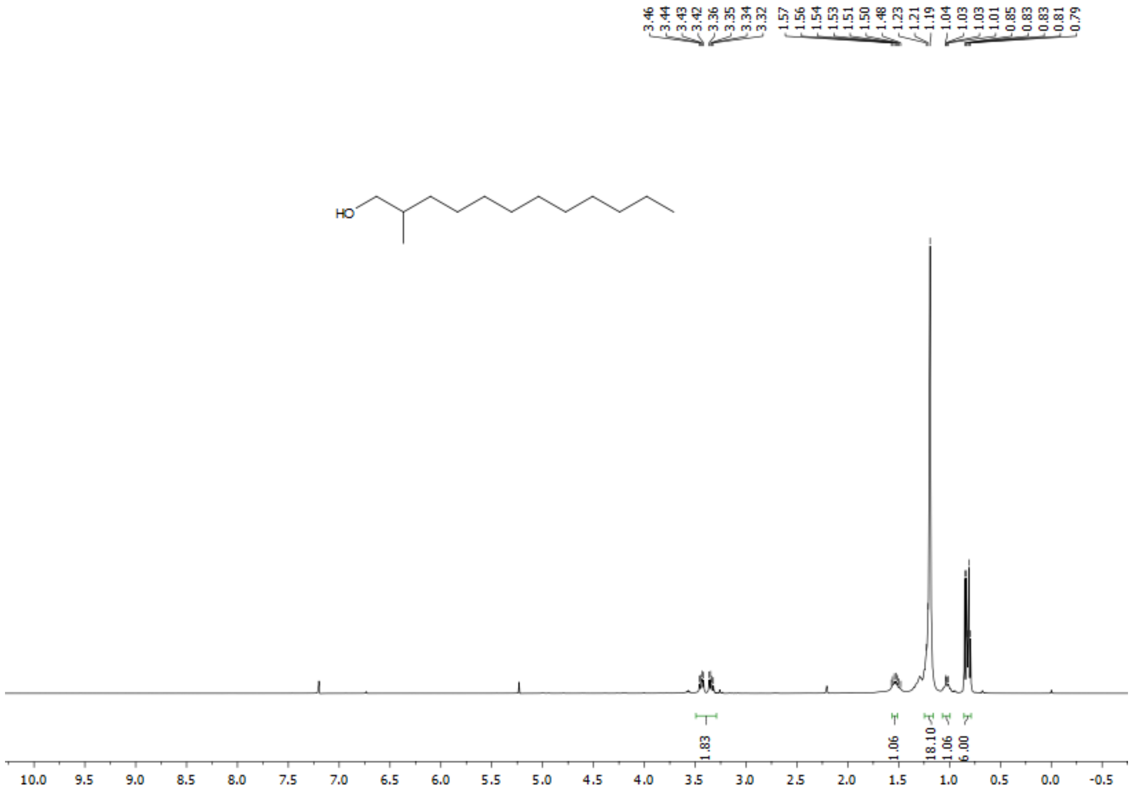

Figure S61: ^{1}H NMR (400 MHz, $CDCl_3$, 298 K) spectrum of 2-methyldodecan-1-ol 10h.

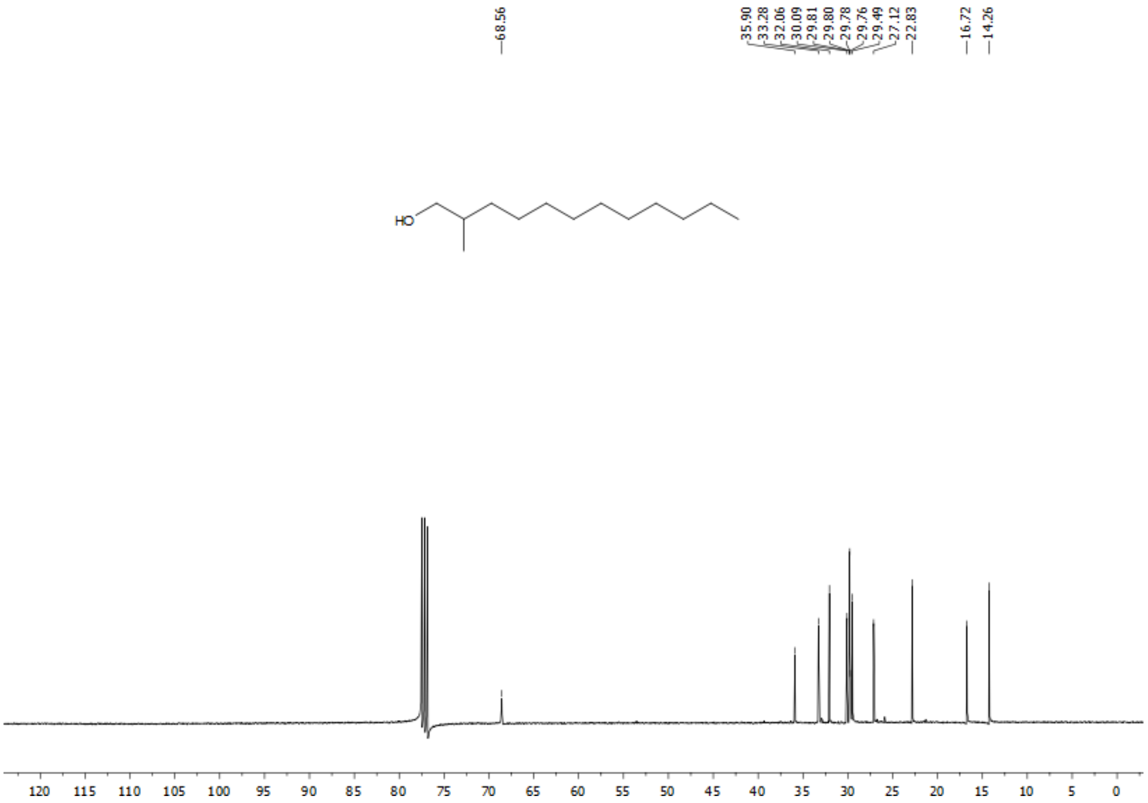

Figure S62: ^{13}C{^{1}H} NMR (101 MHz, $CDCl_3$, 298 K) spectrum of 2-methyldodecan-1-ol 10h.

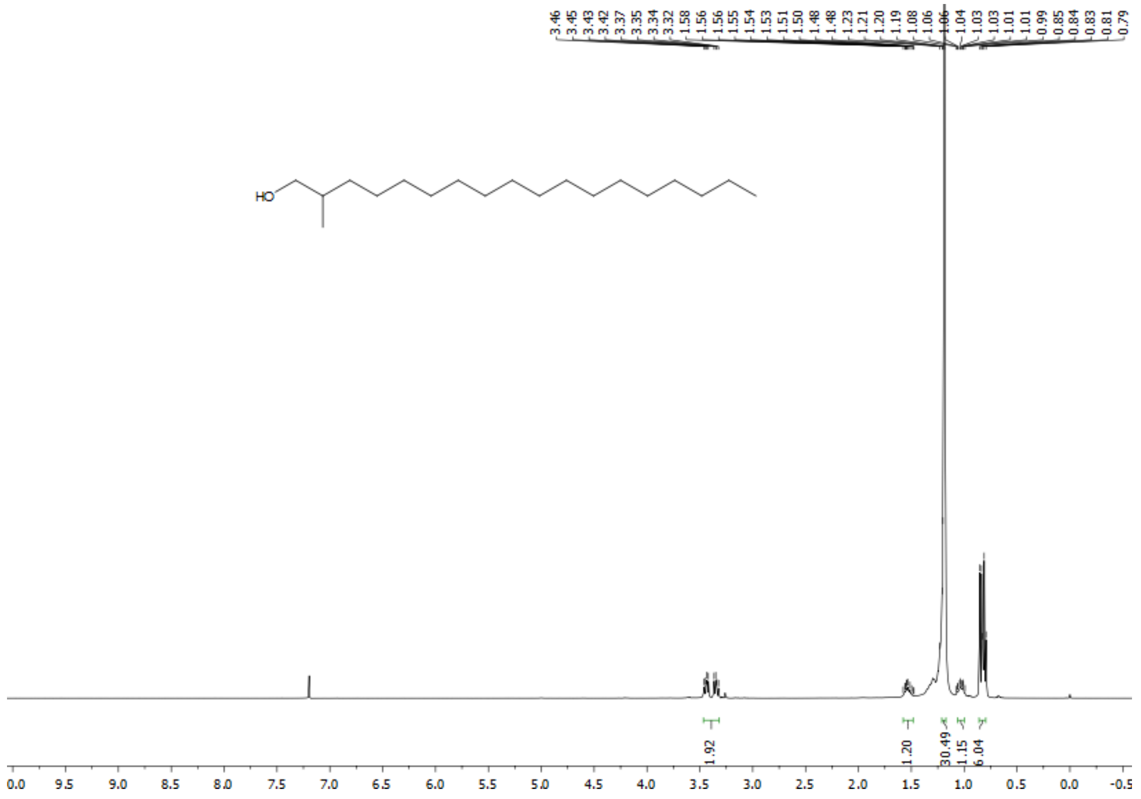

Figure S63: ^{1}H NMR (400 MHz, $CDCl_3$, 298 K) spectrum of 2-methyloctadecan-1-ol 10i.

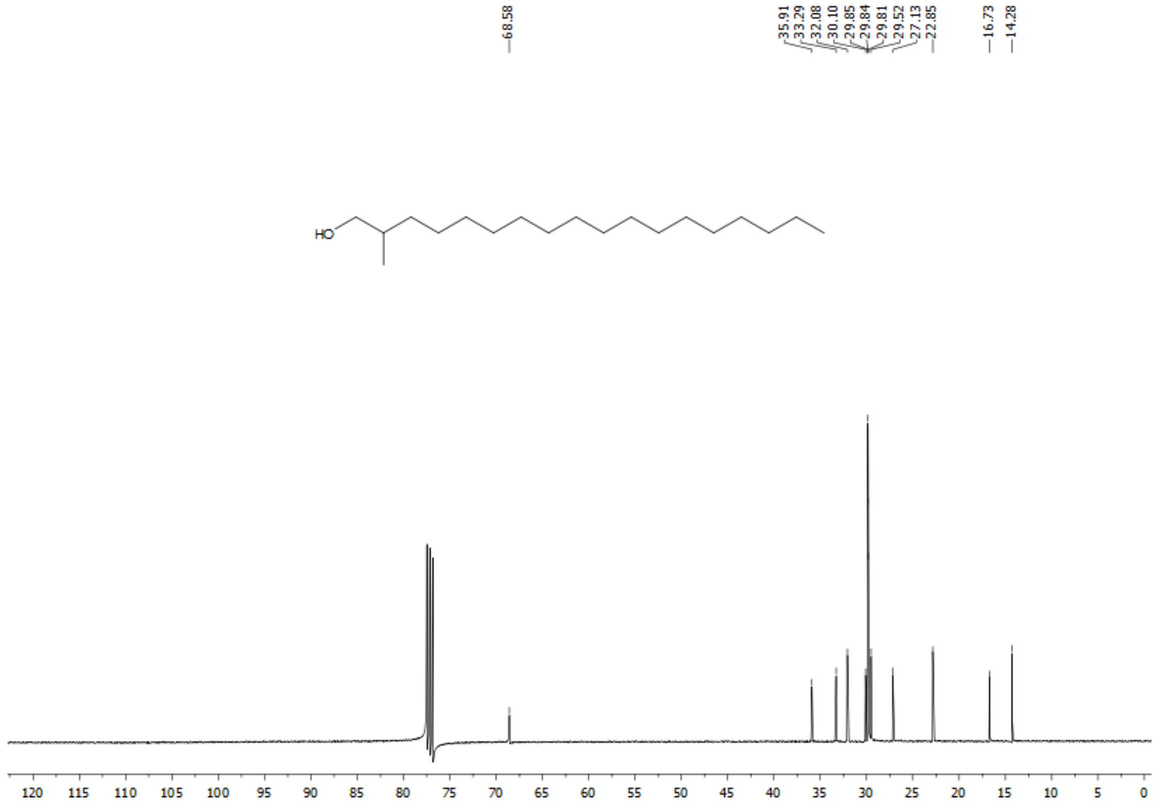

Figure S64: $^{13}C\{^{1}H\}$ NMR (101 MHz, $CDCl_3$, 298 K) spectrum of 2-methyloctadecan-1-ol 10i.

14. ^{1}H NMR reaction mixture spectra of β-methylated products

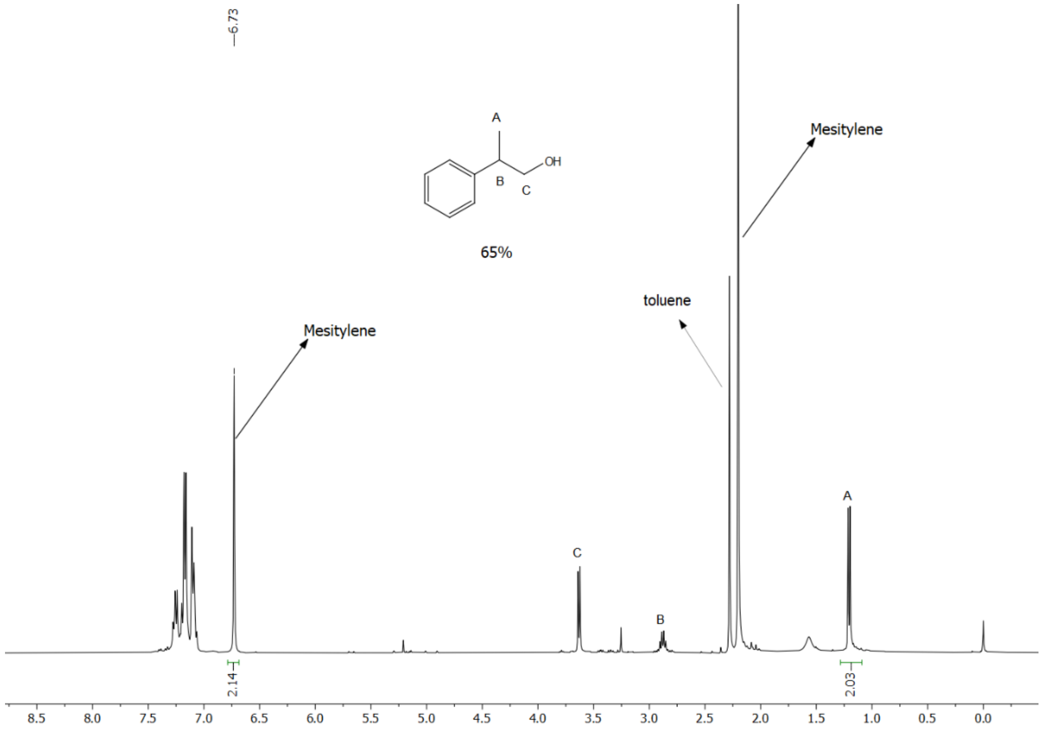

Figure S65: ^{1}H NMR (400 MHz, $CDCl_3$, 298 K) reaction mixture spectrum for table S1, entry 1.

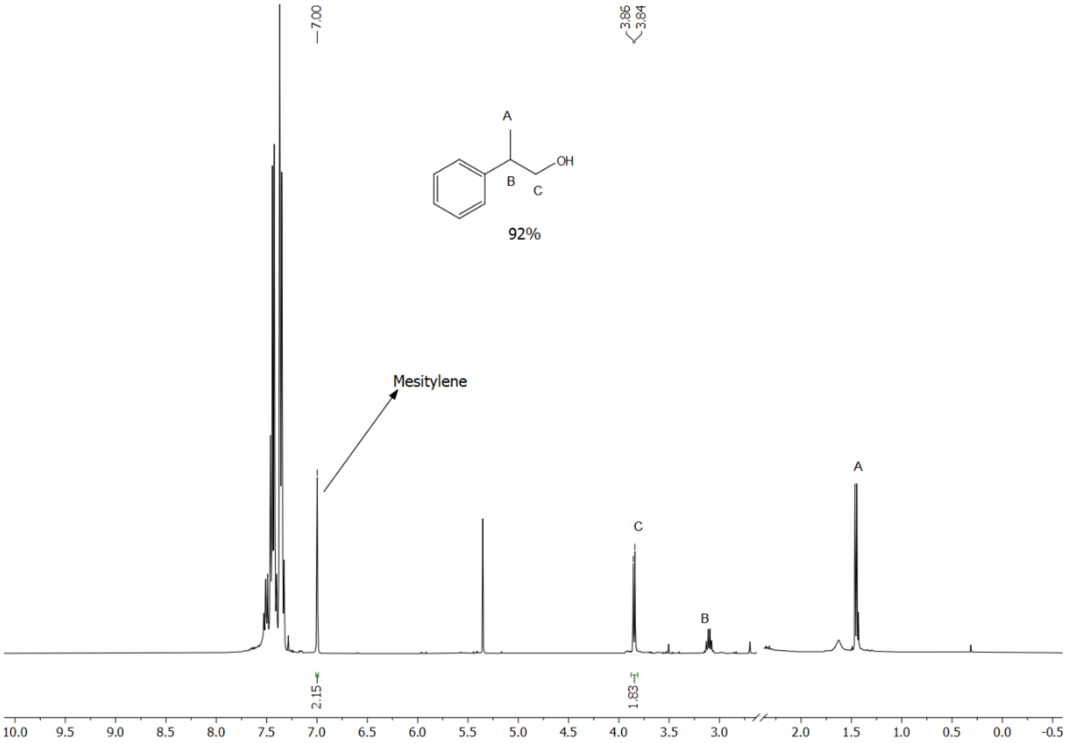

Figure S66: ^{1}H NMR (400 MHz, $CDCl_3$, 298 K) reaction mixture spectrum for table S1, entry 2.

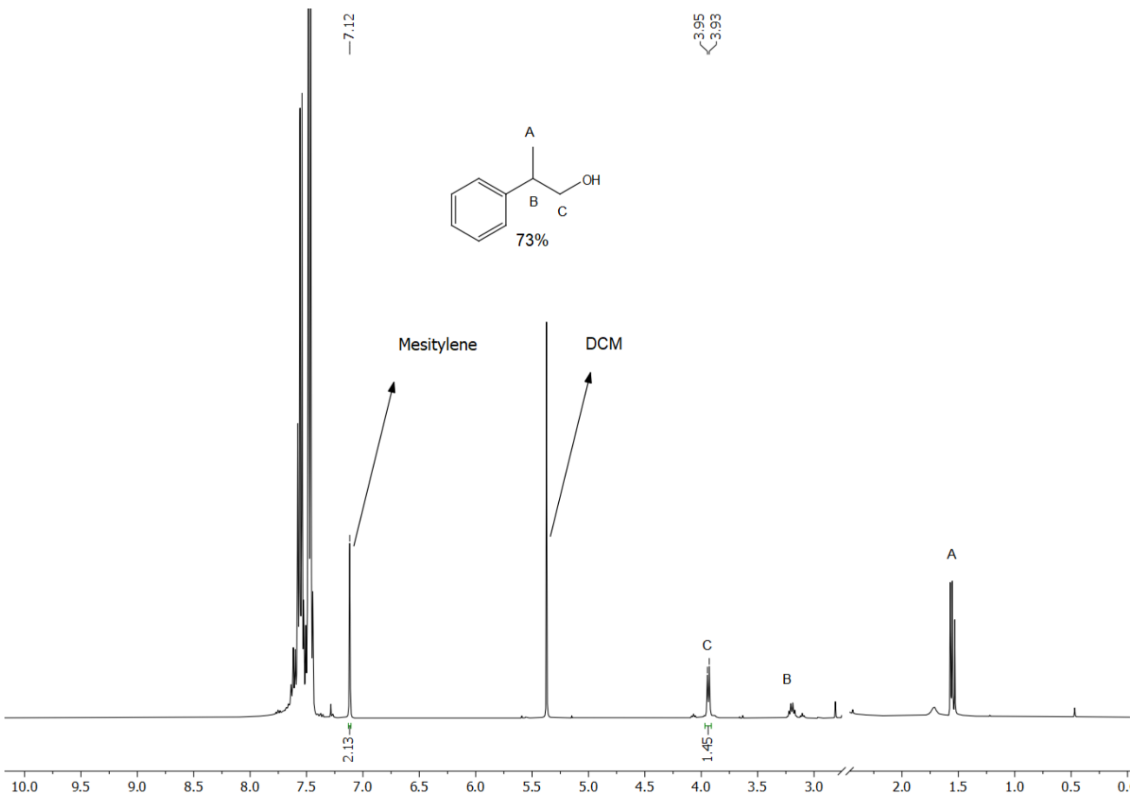

Figure S67: ^{1}H NMR (400 MHz, $CDCl_3$, 298 K) reaction mixture spectrum for table S1, entry 3.

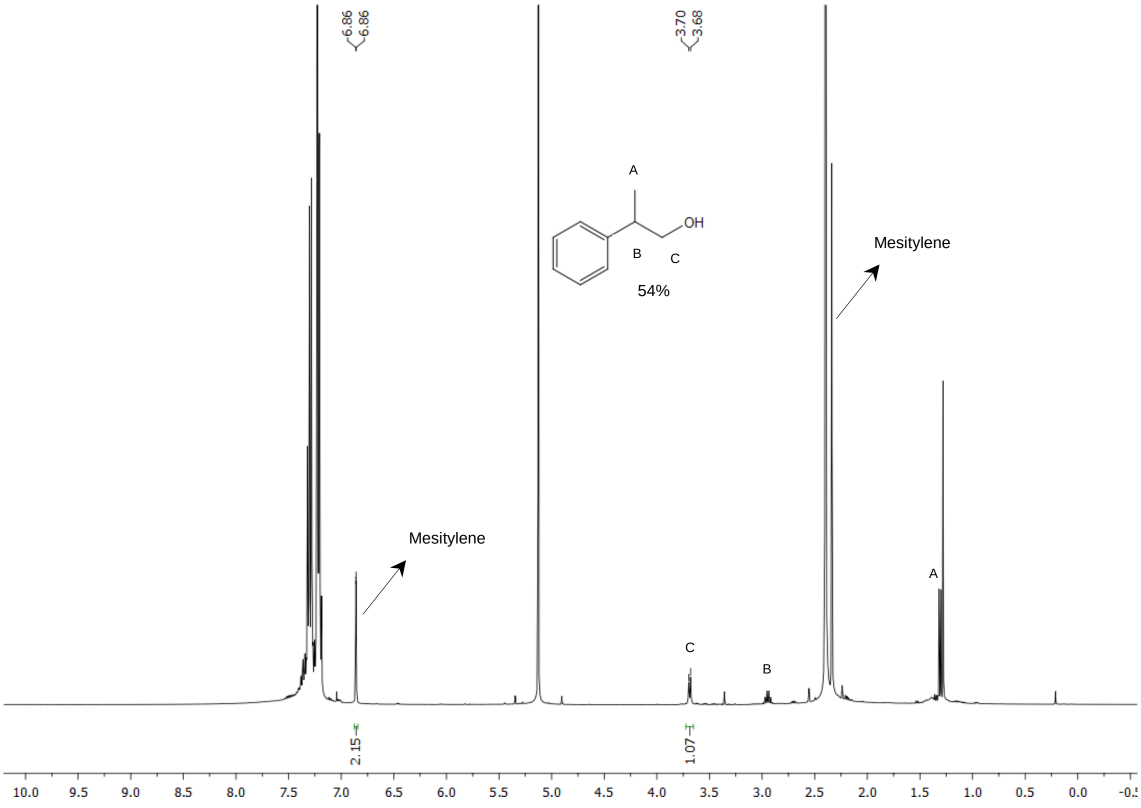

Figure S68: ^{1}H NMR (400 MHz, $CDCl_3$, 298 K) reaction mixture spectrum for table S1, entry 4.

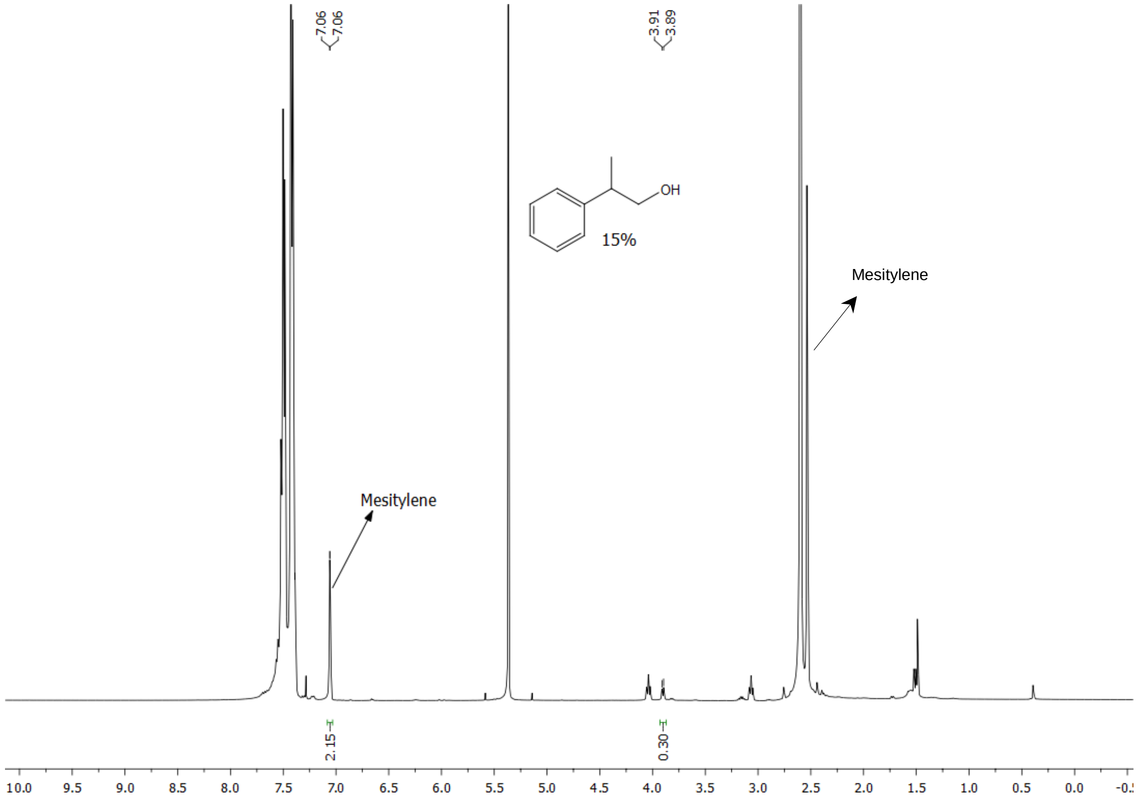

Figure S69: ^{1}H NMR (400 MHz, $CDCl_3$, 298 K) reaction mixture spectrum for table S1, entry 5.

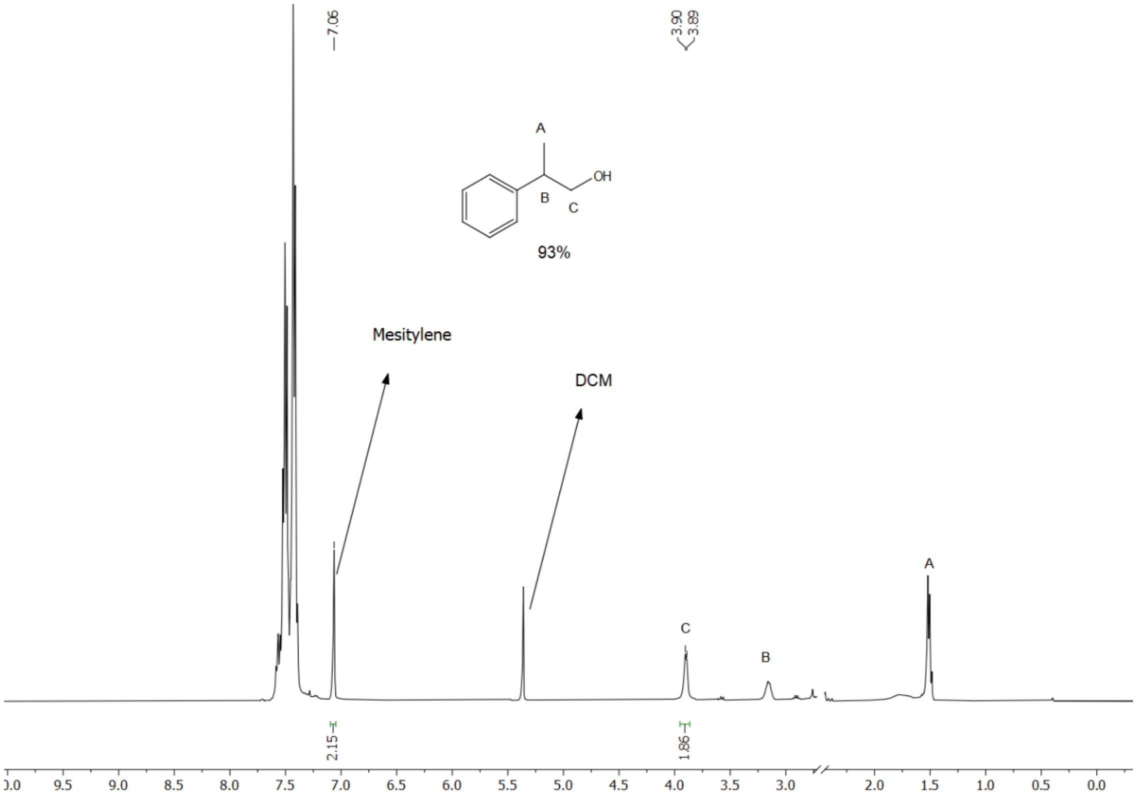

Figure S70: ^{1}H NMR (400 MHz, $CDCl_3$, 298 K) reaction mixture spectrum for table S1, entry 6.

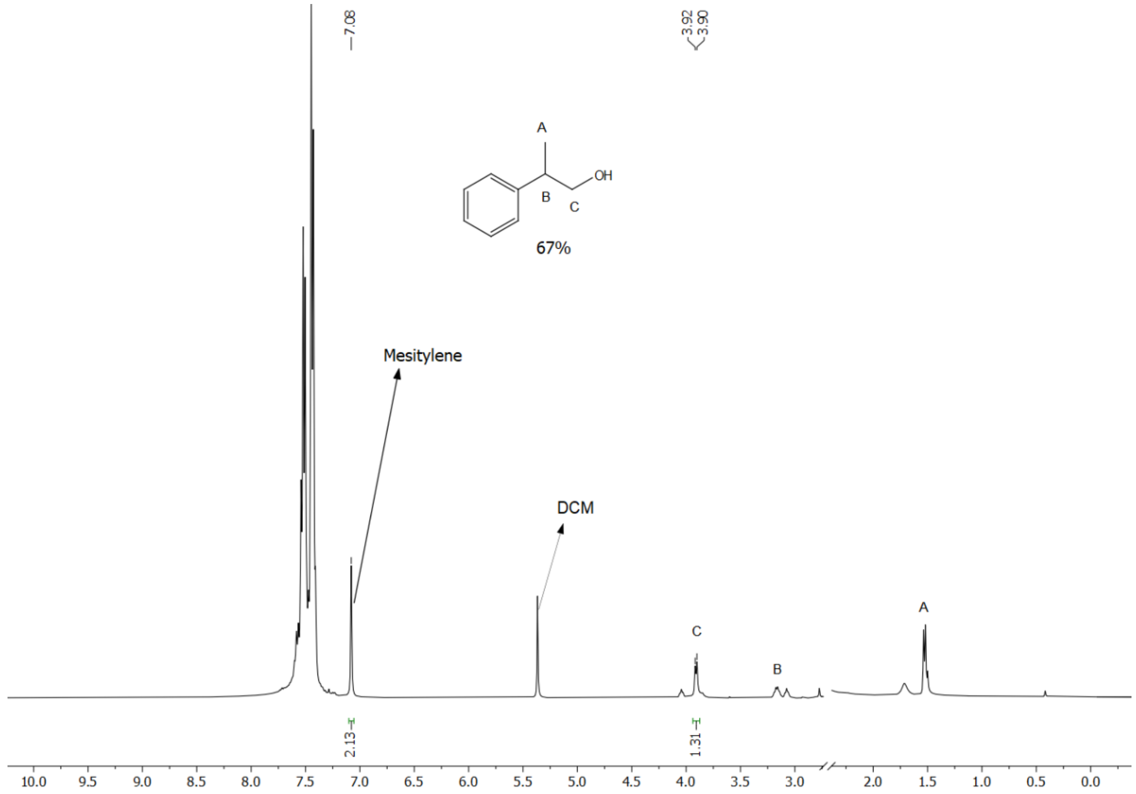

Figure S71: ^{1}H NMR (400 MHz, $CDCl_3$, 298 K) reaction mixture spectrum for table S1, entry 7.

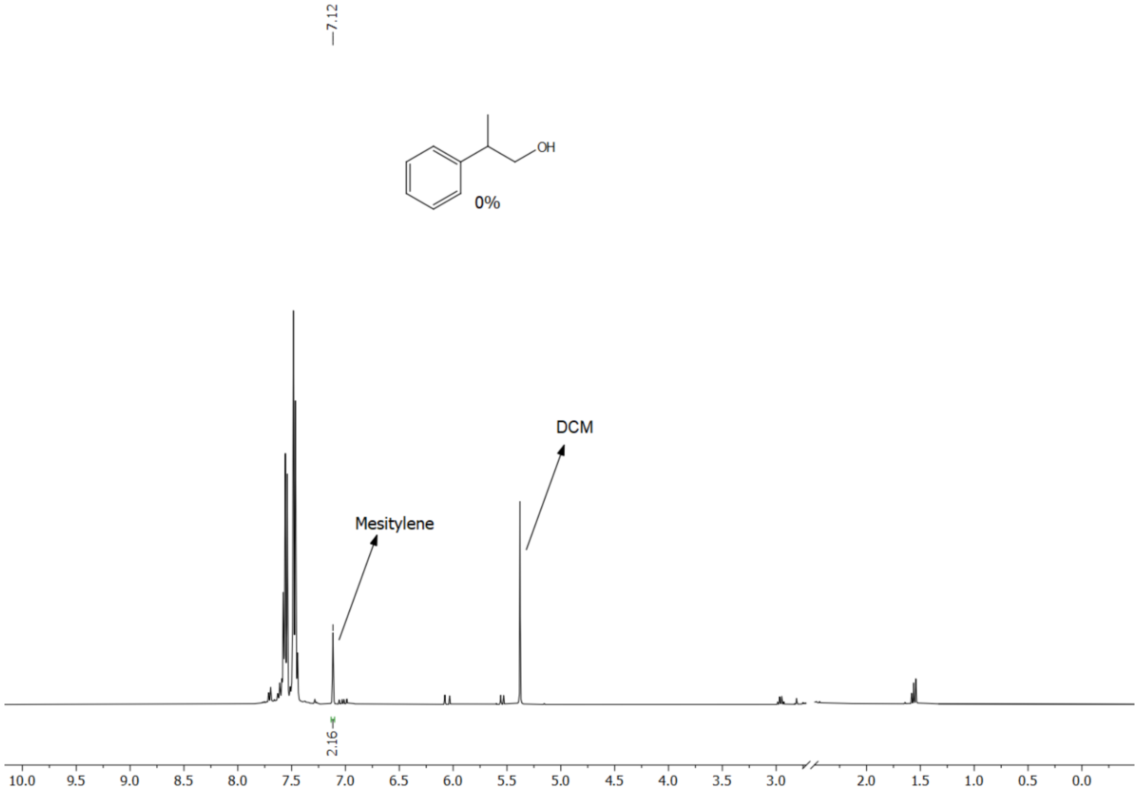

Figure S72: ^{1}H NMR (400 MHz, $CDCl_3$, 298 K) reaction mixture spectrum for table S1, entry 8.

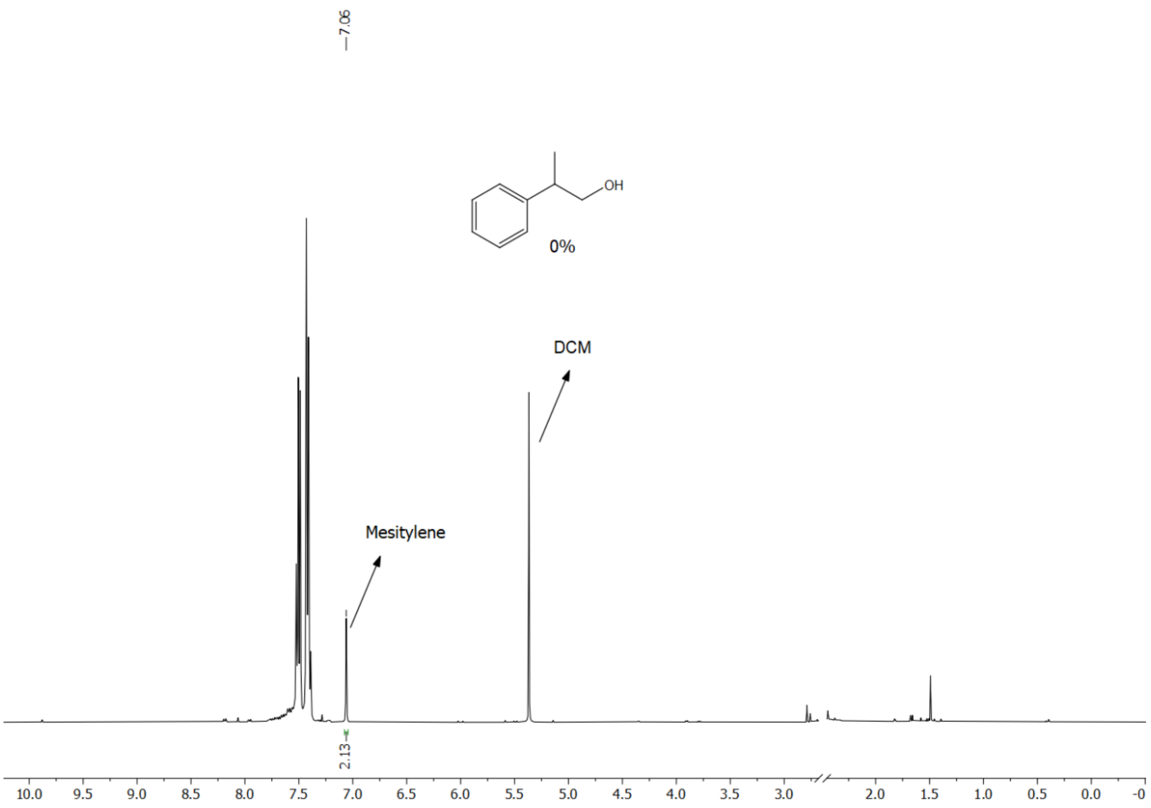

Figure S73: ^{1}H NMR (400 MHz, $CDCl_3$, 298 K) reaction mixture spectrum for table S1, entry 9.

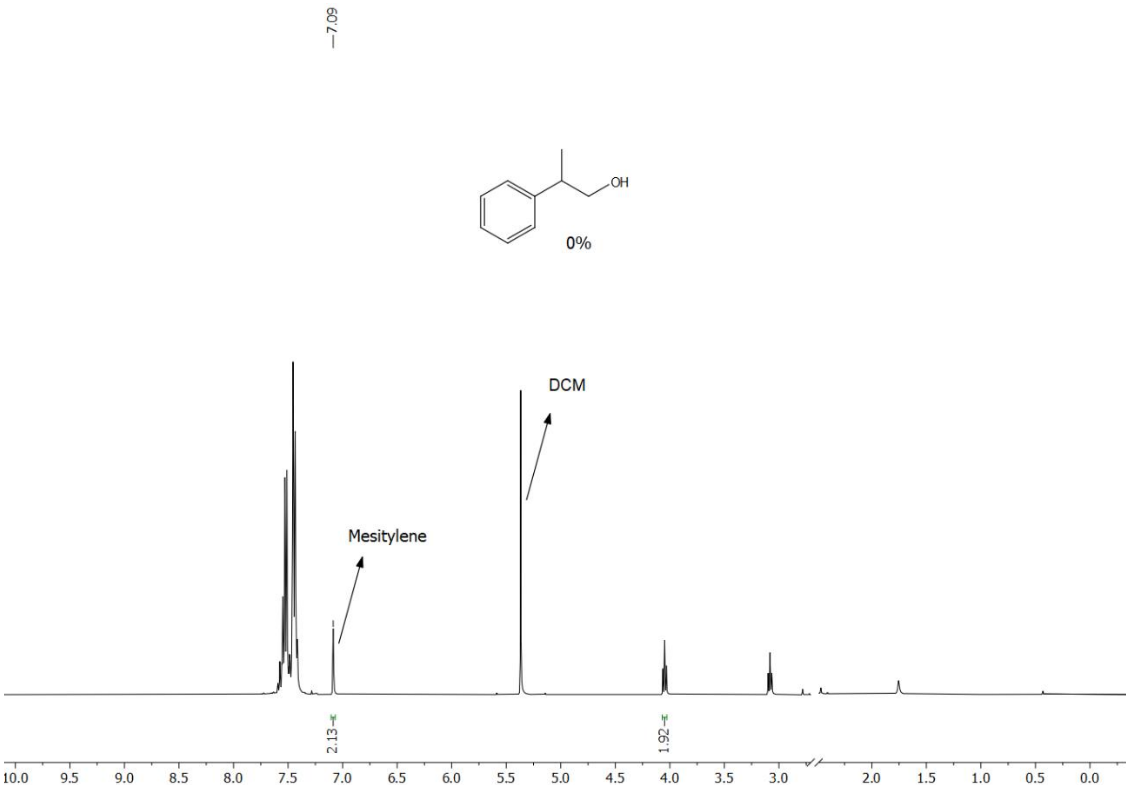

Figure S74:^{1}H NMR (400 MHz, $CDCl_3$, 298 K) reaction mixture spectrum for table S1, entry 10.

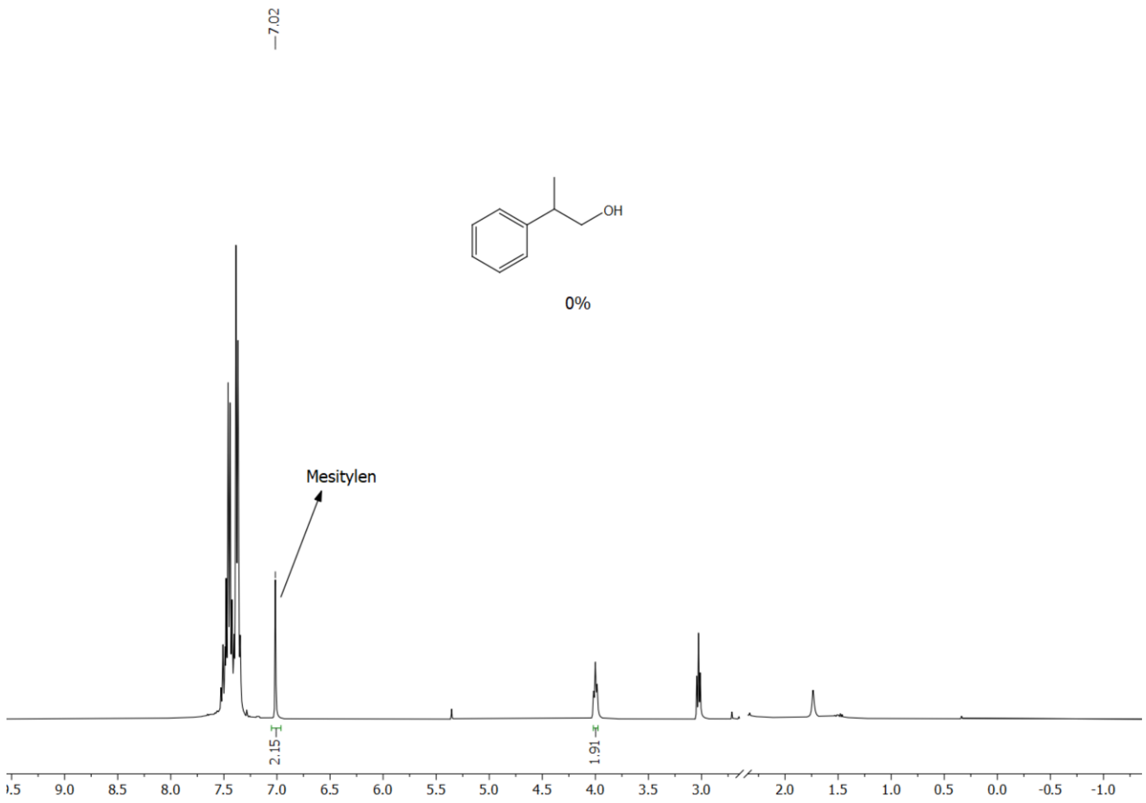

Figure S75: ^{1}H NMR (400 MHz, $CDCl_3$, 298 K) reaction mixture spectrum for table S1, entry 11.

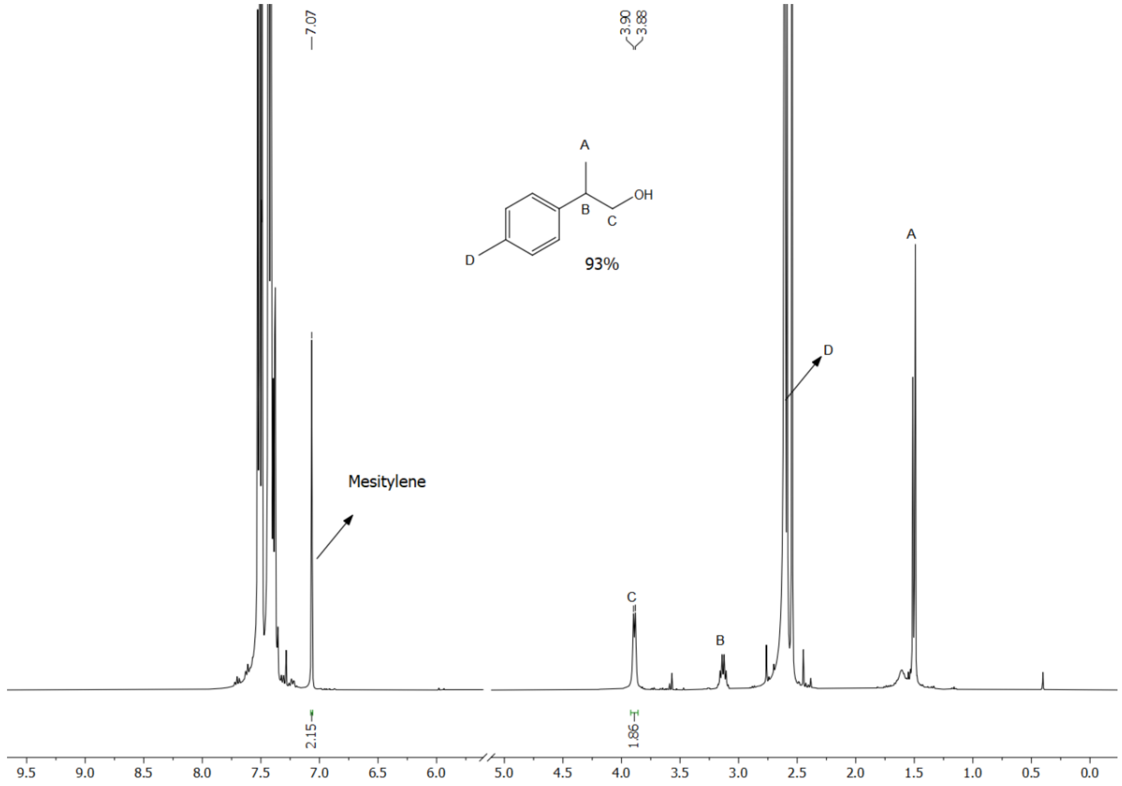

Figure S76: ^{1}H NMR (400 MHz, $CDCl_3$, 298 K) reaction mixture spectrum of 2-(*p*-tolyl)propan-1-ol 3b.

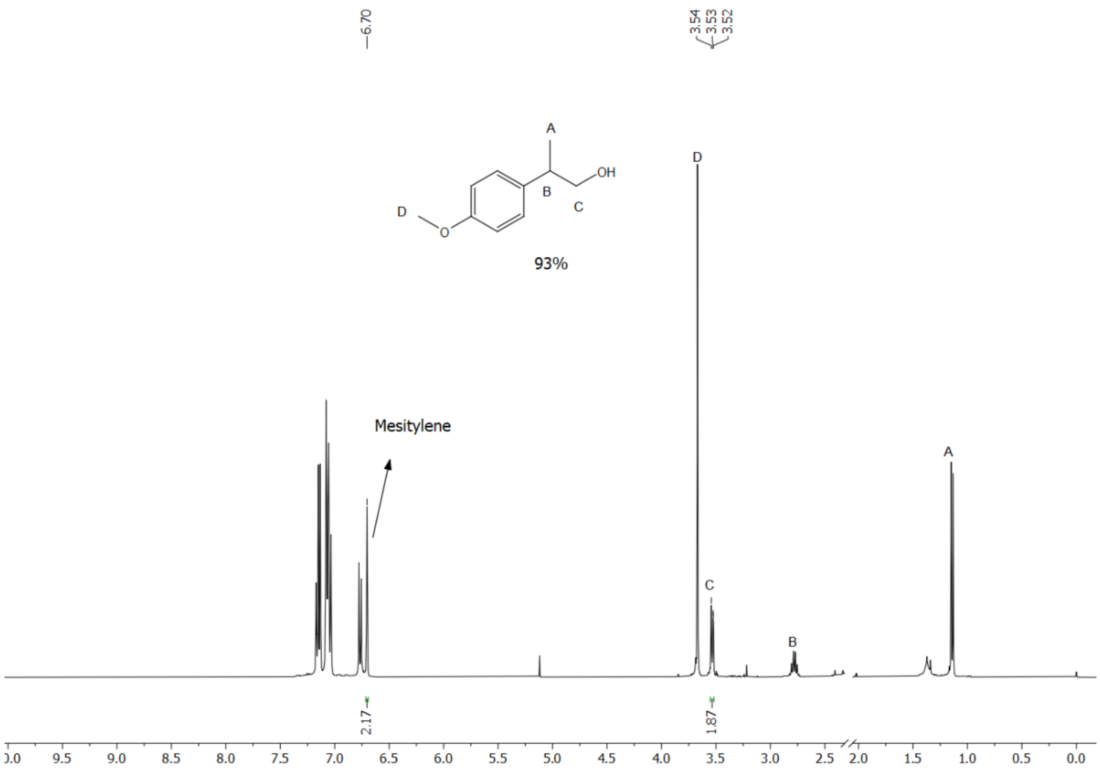

Figure S77: ^{1}H NMR (400 MHz, $CDCl_3$, 298 K) reaction mixture spectrum of 2-(4-methoxyphenyl)propan-1-ol 3d.

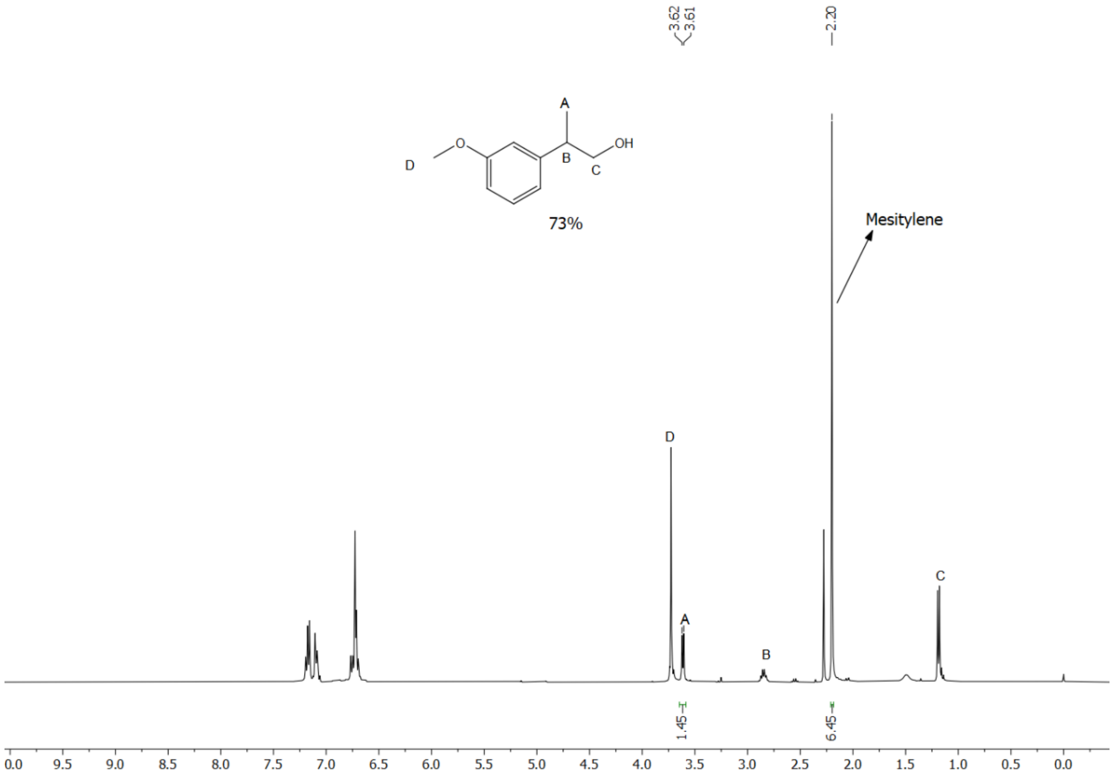

Figure S78: ^{1}H NMR (400 MHz, $CDCl_3$, 298 K) reaction mixture spectrum of 2-(3-methoxyphenyl)propan-1-ol 3e.

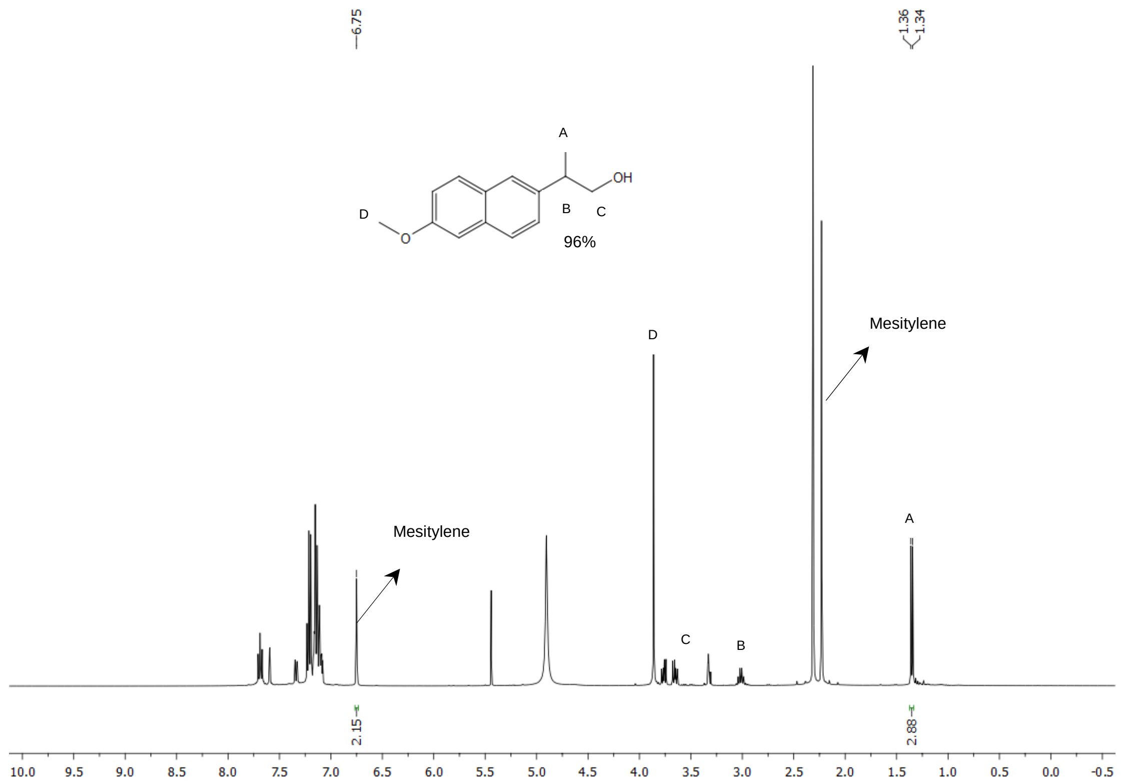

Figure S79: ^{1}H NMR (400 MHz, CD_3OD, 298 K) reaction mixture spectrum of 2-(6-methoxynaphthalen-2-yl)propan-1-ol 3f.

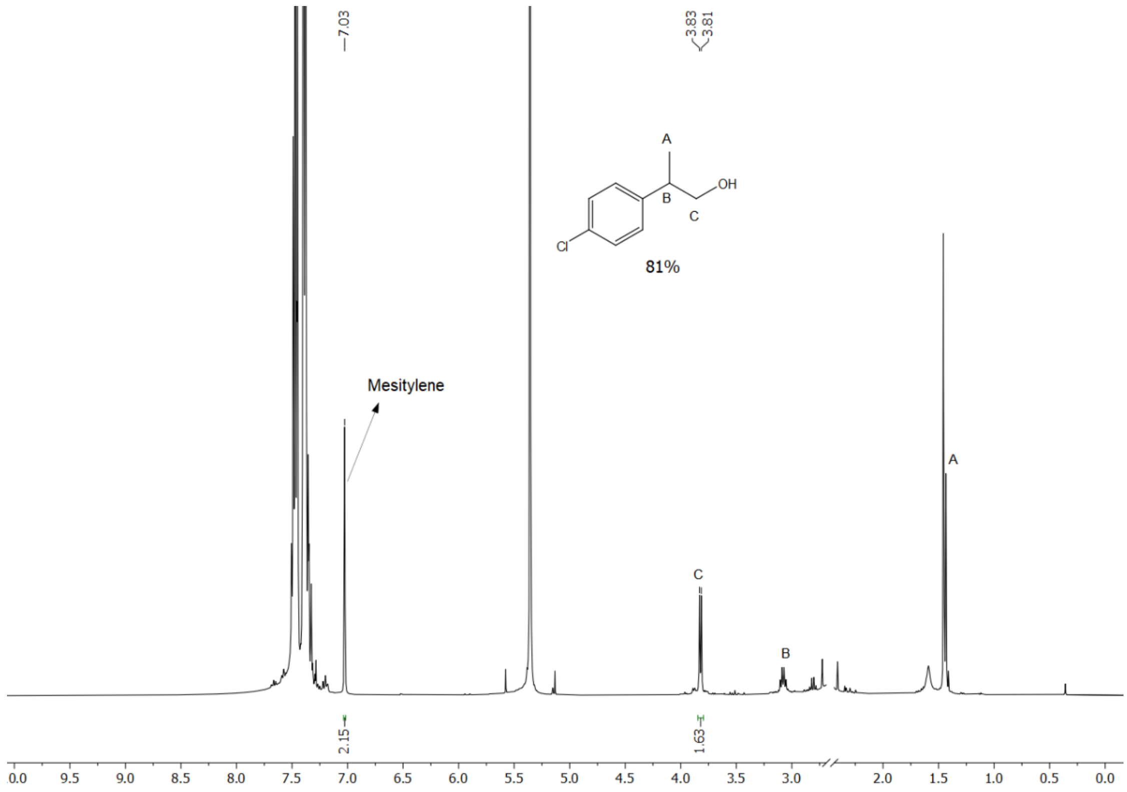

Figure S80: ^{1}H NMR (400 MHz, $CDCl_3$, 298 K) reaction mixture spectrum of 2-(4-chlorophenyl)propan-1-ol 3g.

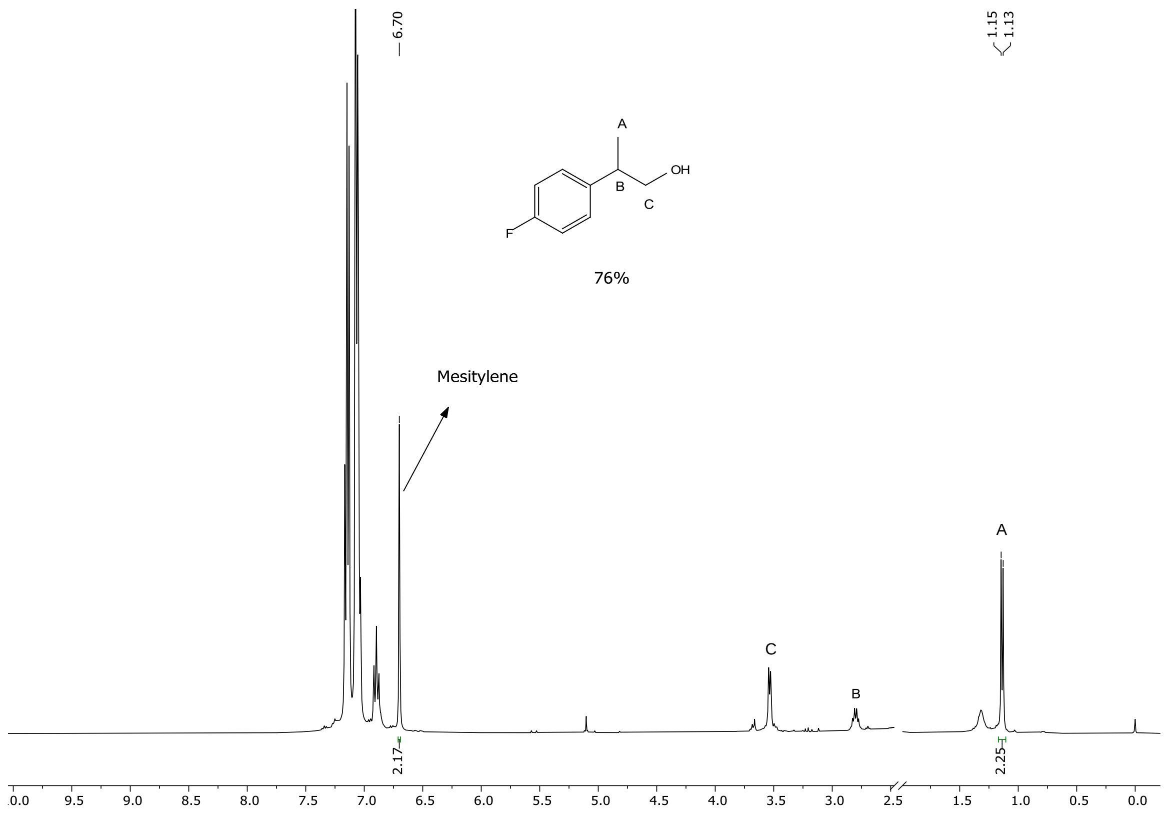

Figure S81: ^{1}H NMR (400 MHz, $CDCl_3$, 298 K) reaction mixture spectrum of 2-(4-fluorophenyl)propan-1-ol 3h.

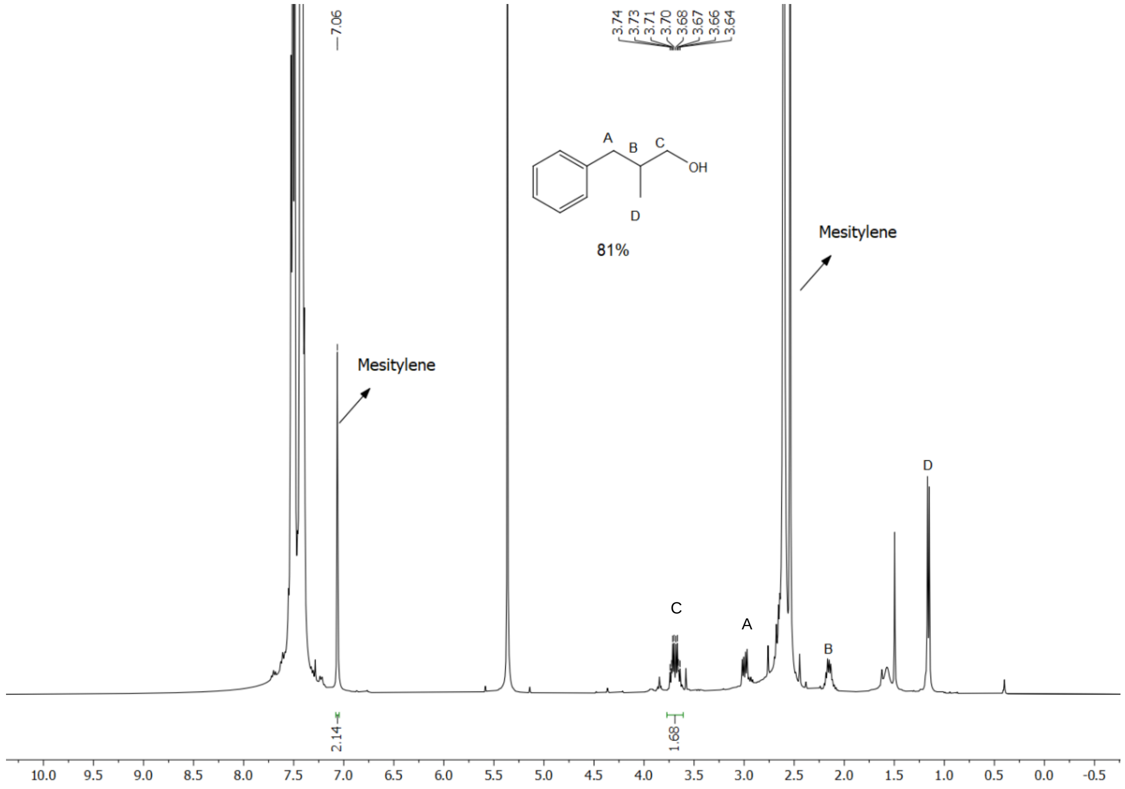

Figure S82: ^{1}H NMR (400 MHz, $CDCl_3$, 298 K) reaction mixture spectrum of 2-methyl-3-phenylpropan-1-ol 3j.

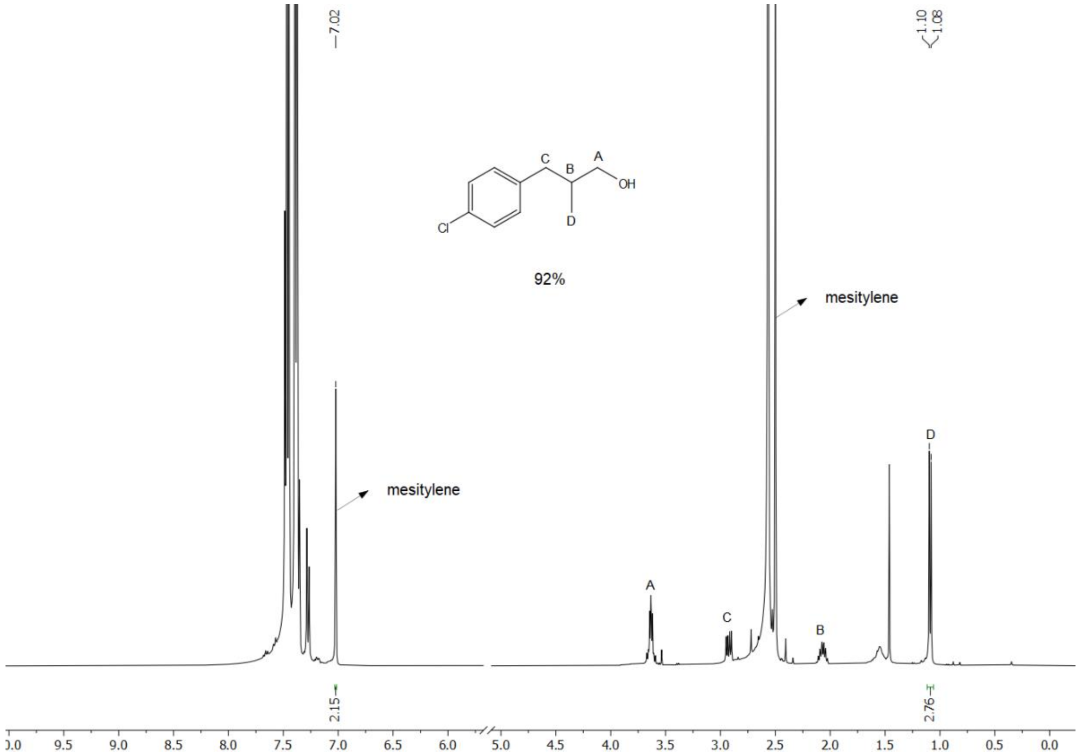

Figure S83: ^{1}H NMR (400 MHz, CDCl$_3$, 298 K) reaction mixture spectrum of3-(4-chlorophenyl)-2-methylpropan-1-ol 3k.

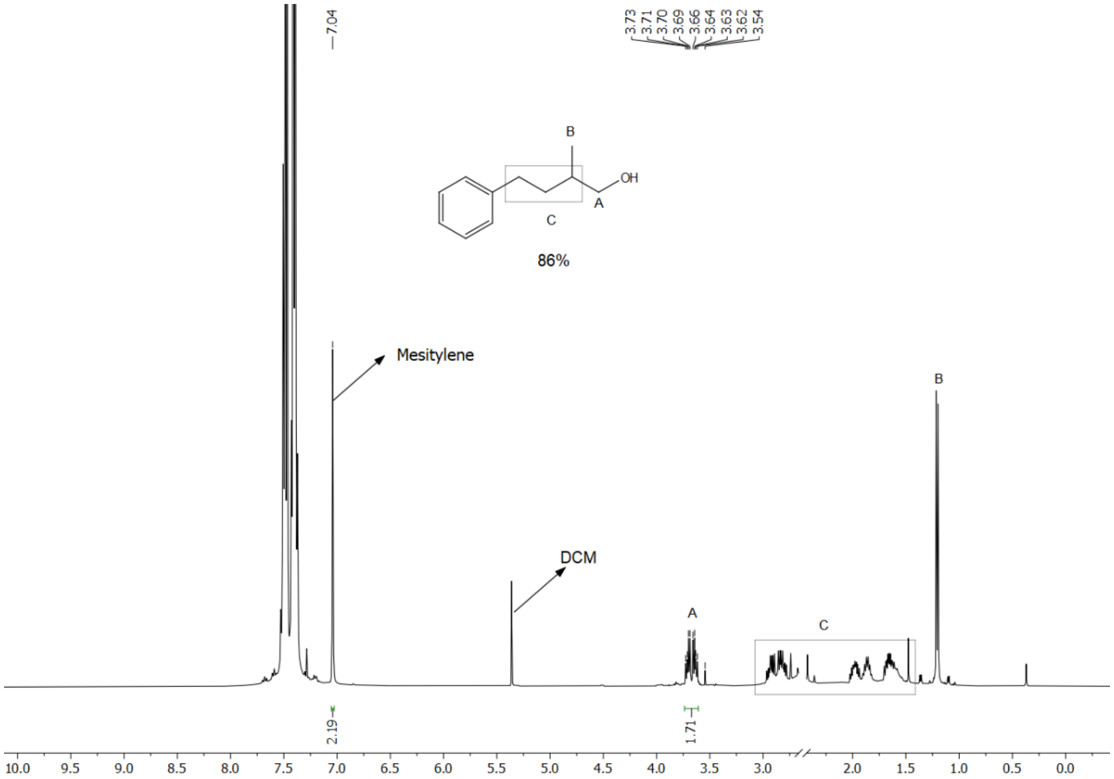

Figure S84: ^{1}H NMR (400 MHz, CDCl$_3$, 298 K) reaction mixture spectrum of 2-methyl-4-phenylbutan-1-ol 3l.

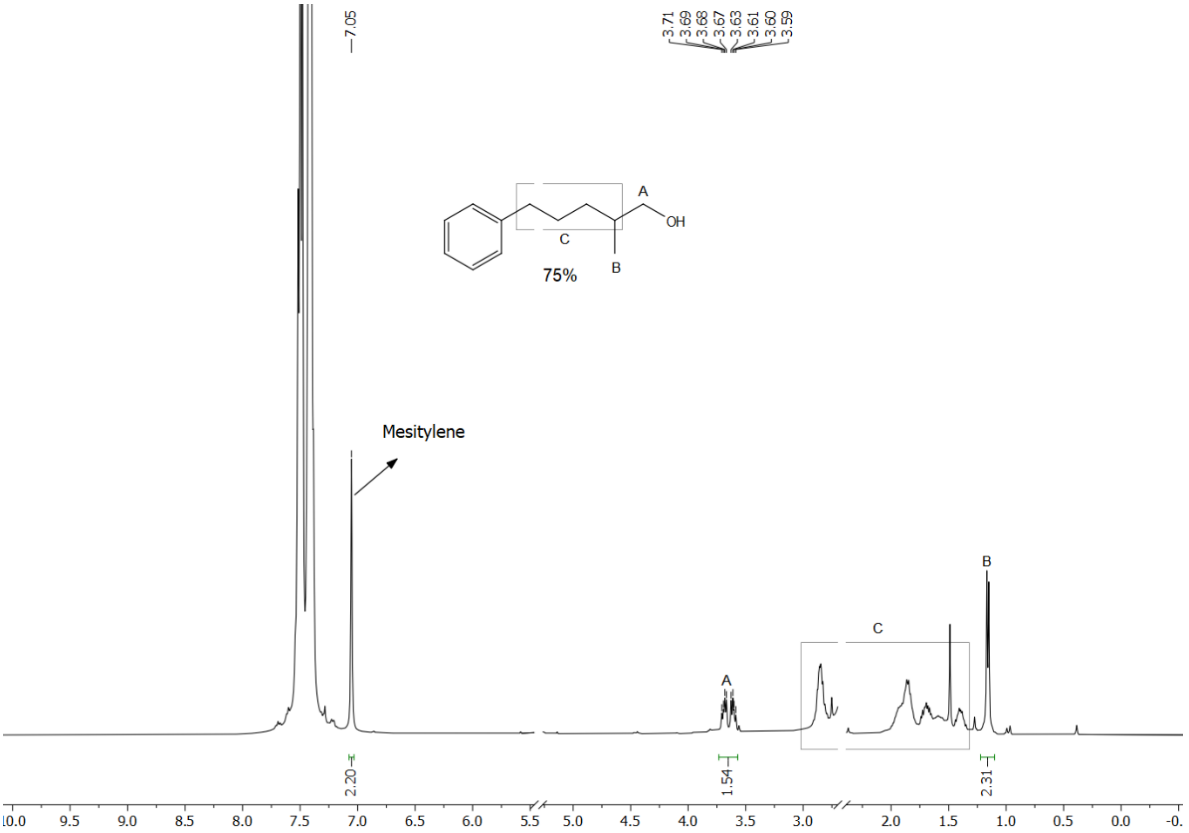

Figure S85: ^{1}H NMR (400 MHz, $CDCl_3$, 298 K) reaction mixture spectrum of 2-methyl-5-phenylpentan-1-ol 3m.

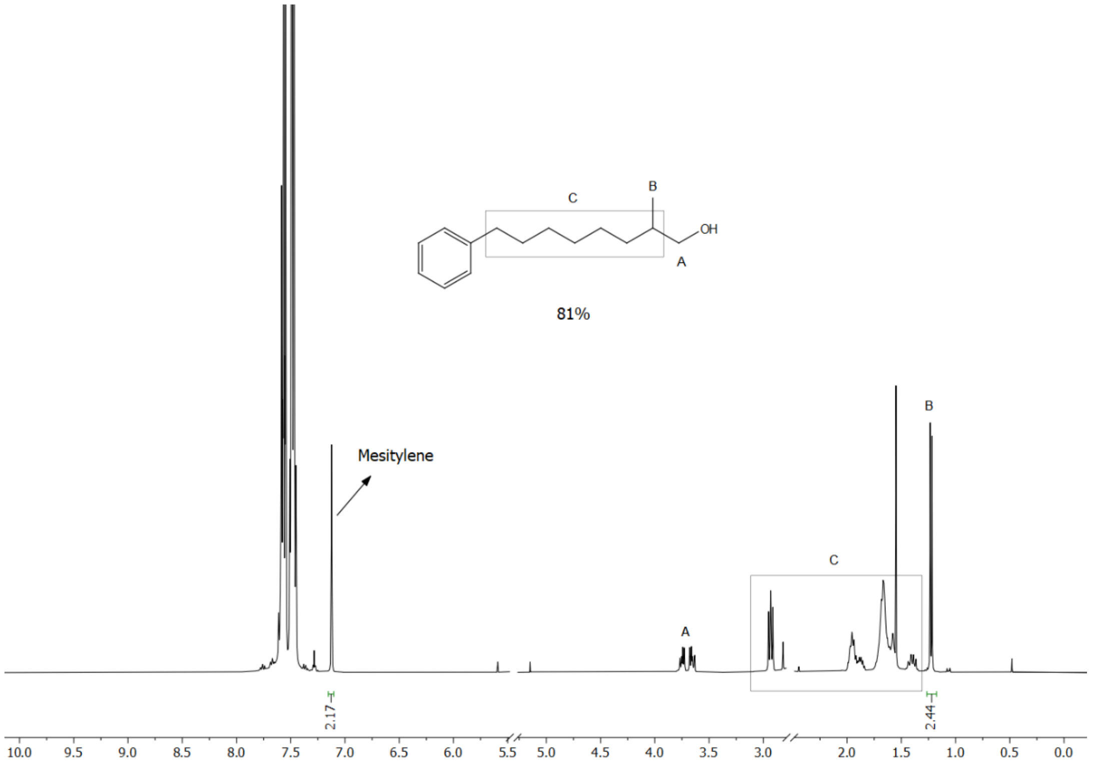

Figure S86: ^{1}H NMR (400 MHz, $CDCl_3$, 298 K) reaction mixture spectrum of2-methyl-8-phenyloctan-1-ol 3n.

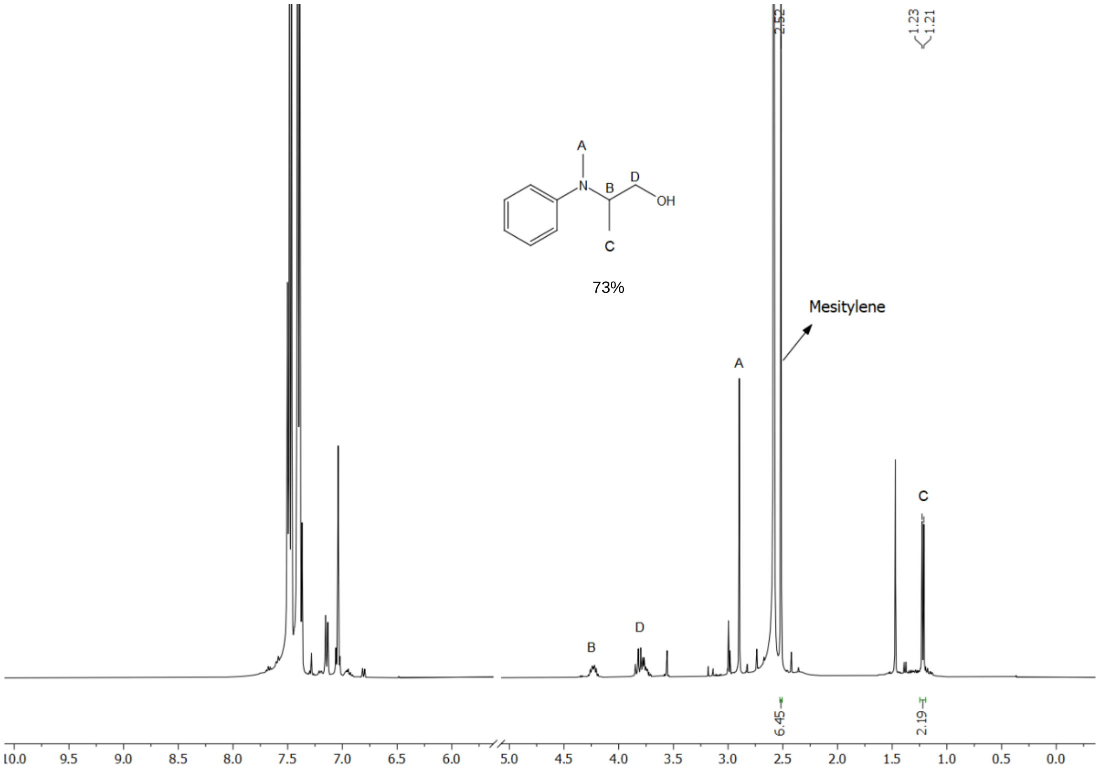

Figure S87: ^{1}H NMR (400 MHz, $CDCl_3$, 298 K) reaction mixture spectrum of 2-(methyl(phenyl)amino)propan-1-ol 3o.

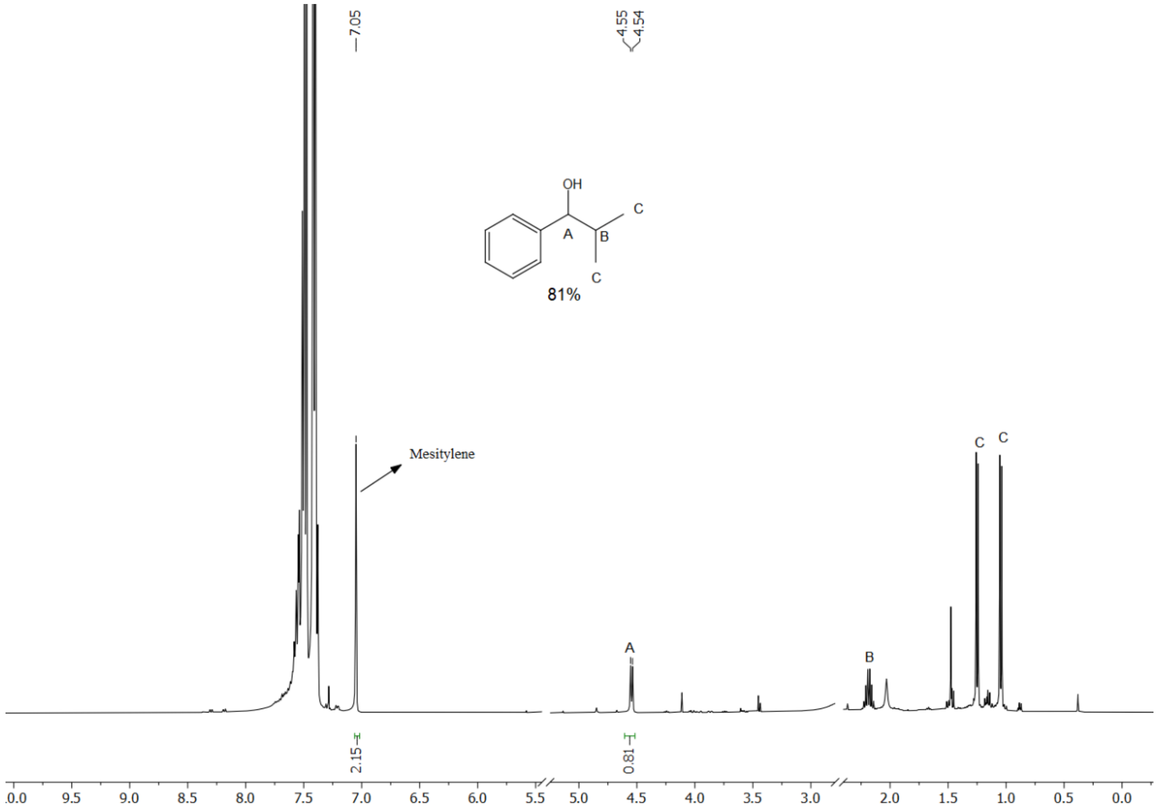

Figure S88: ^{1}H NMR (400 MHz, $CDCl_3$, 298 K) reaction mixture spectrum of 2-methyl-1-phenylpropan-1-ol 8a.

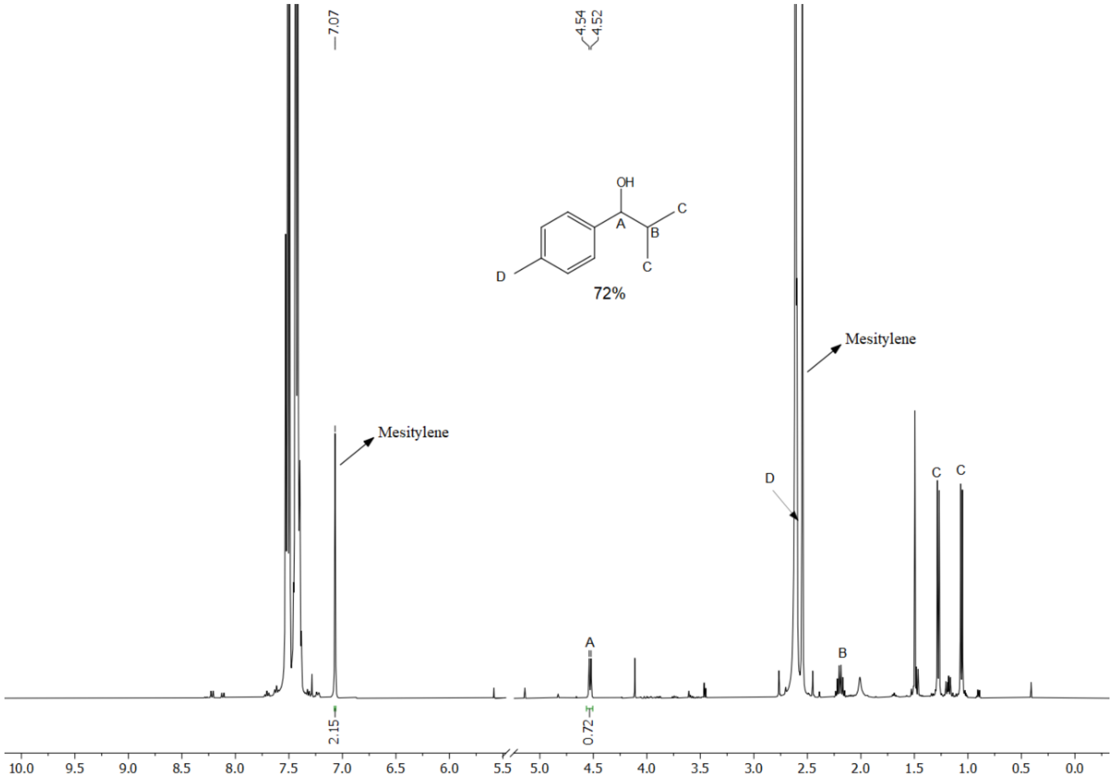

Figure S89: ^{1}H NMR (400 MHz, $CDCl_3$, 298 K) reaction mixture spectrum of 2-methyl-1-(p-tolyl)propan-1-ol 8b.

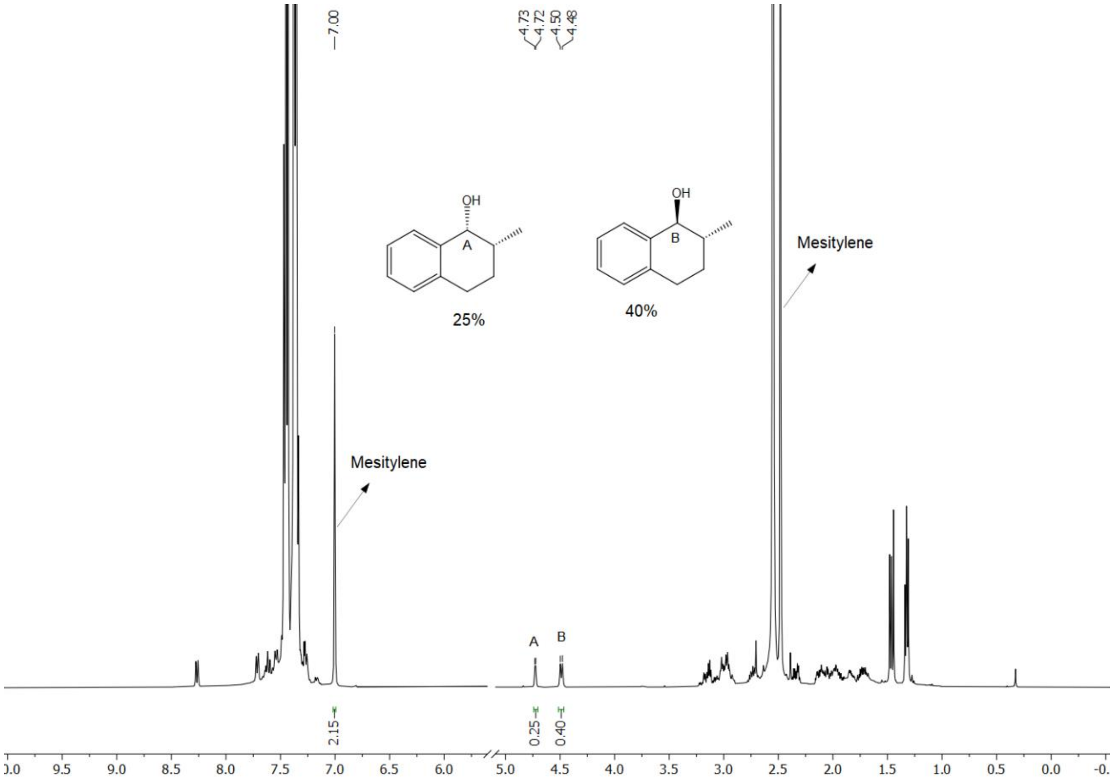

Figure S90: ^{1}H NMR (400 MHz, CDCl$_3$, 298 K) reaction mixture spectrum of 2-methyl-1,2,3,4-tetrahydronaphthalen-1-ol 8c.

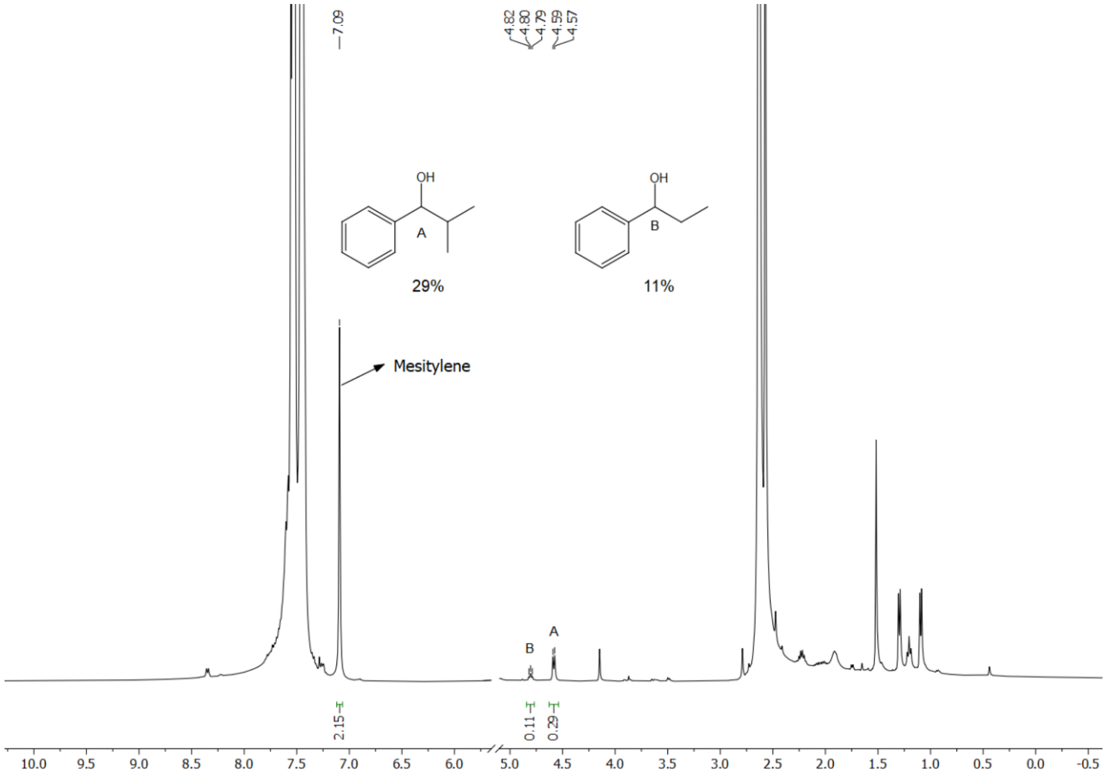

Figure S91: ^{1}H NMR (400 MHz, CDCl$_3$, 298 K) reaction mixture spectrum for table S2, entry 1.

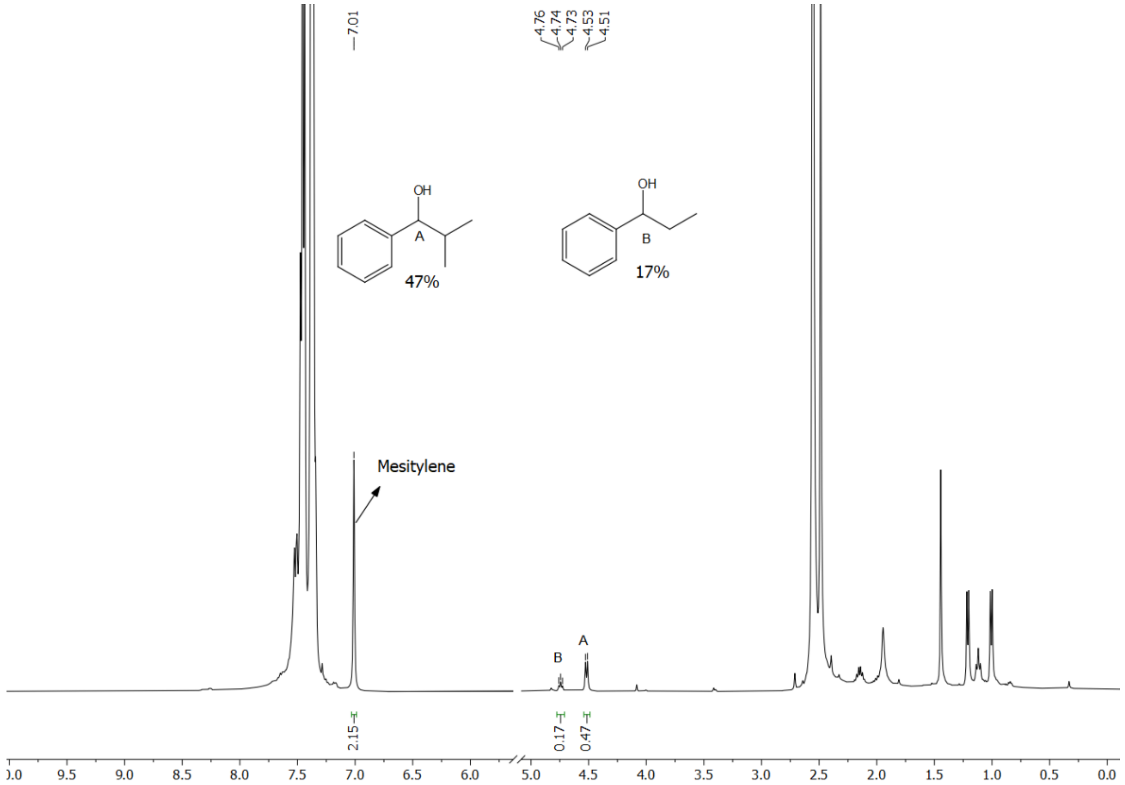

Figure S92: ^{1}H NMR (400 MHz, $CDCl_3$, 298 K) reaction mixture spectrum for table S2, entry 2.

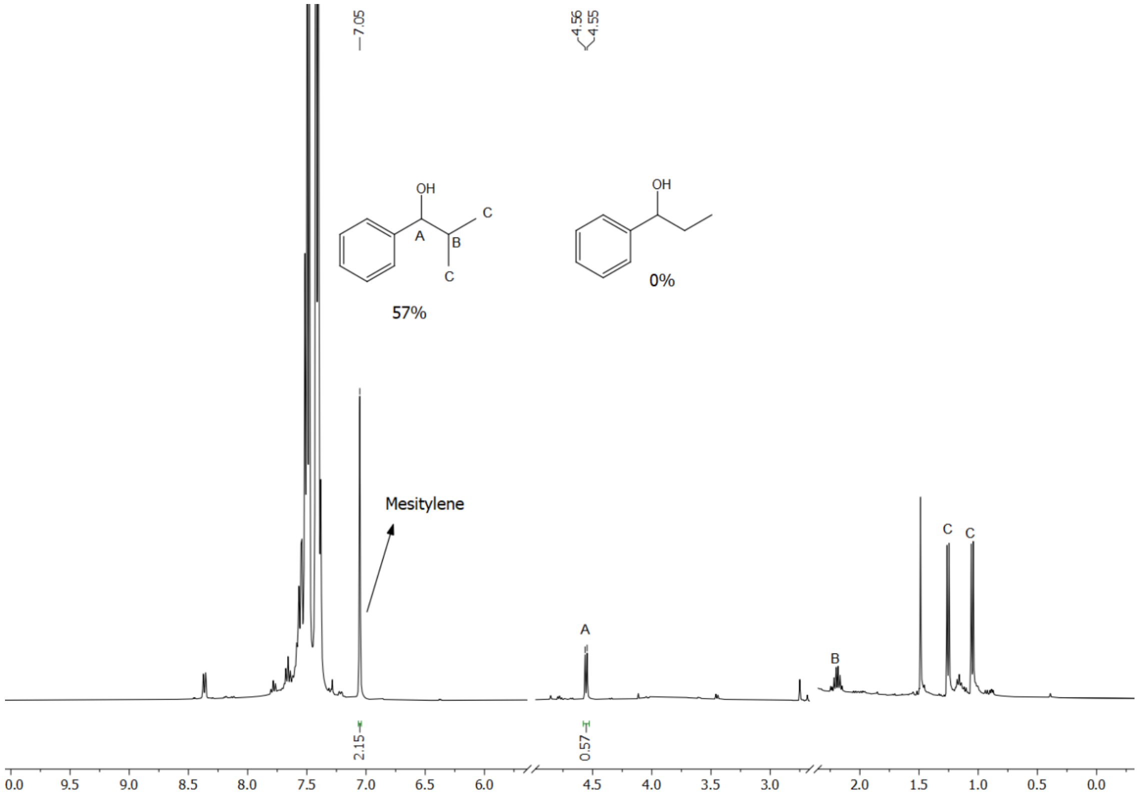

Figure S93: ^{1}H NMR (400 MHz, $CDCl_3$, 298 K) reaction mixture spectrum for table S2, entry 3.

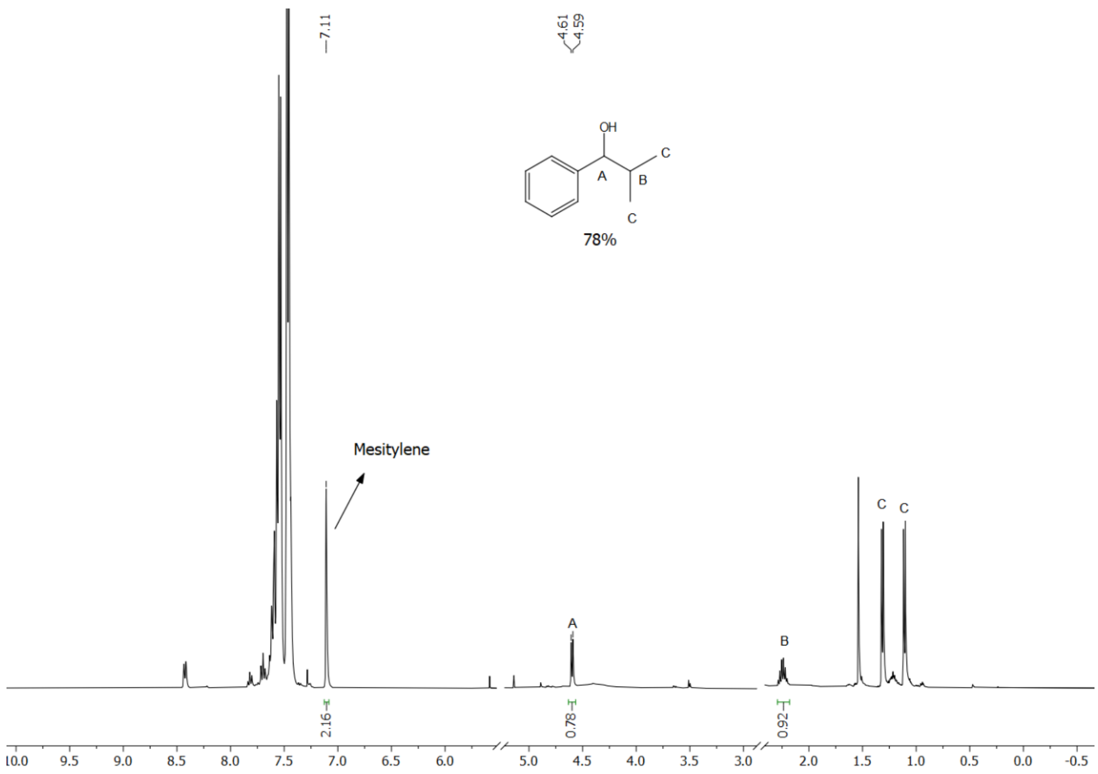

Figure S94: ^{1}H NMR (400 MHz, $CDCl_3$, 298 K) reaction mixture spectrum for table S2, entry 4.

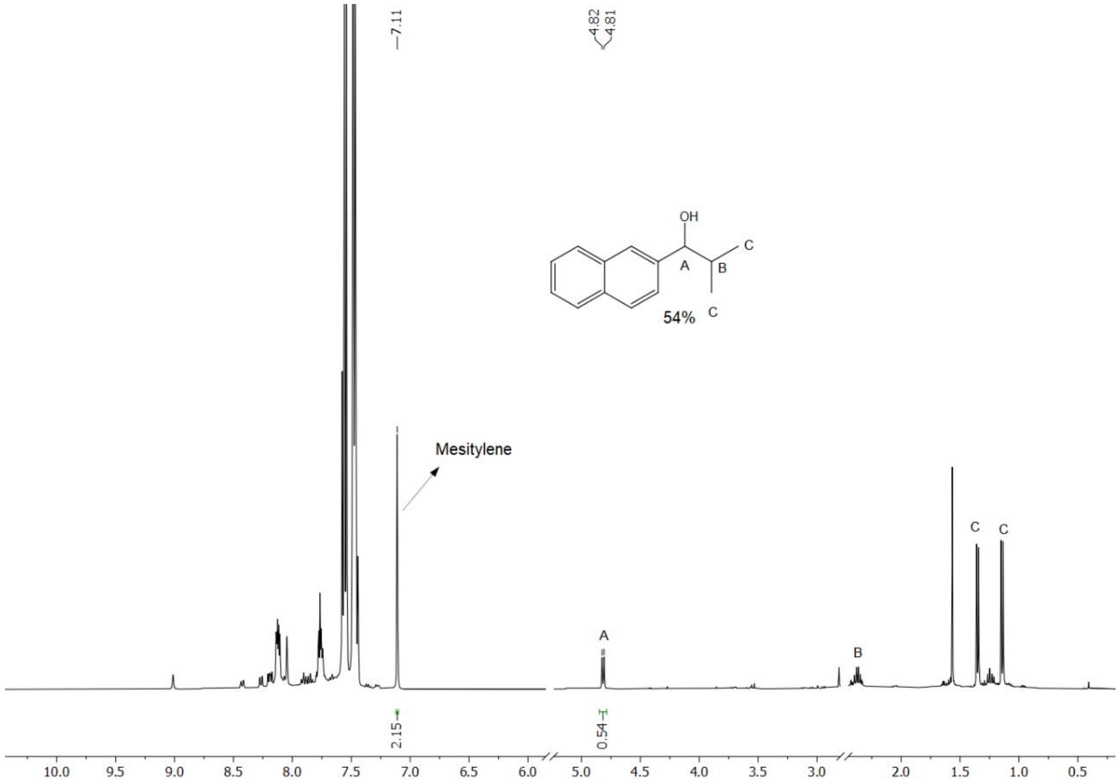

Figure S95: ^{1}H NMR (400 MHz, $CDCl_3$, 298 K) reaction mixture spectrum of 2-methyl-1-(naphthalen-2-yl)propan-1-ol 8e.

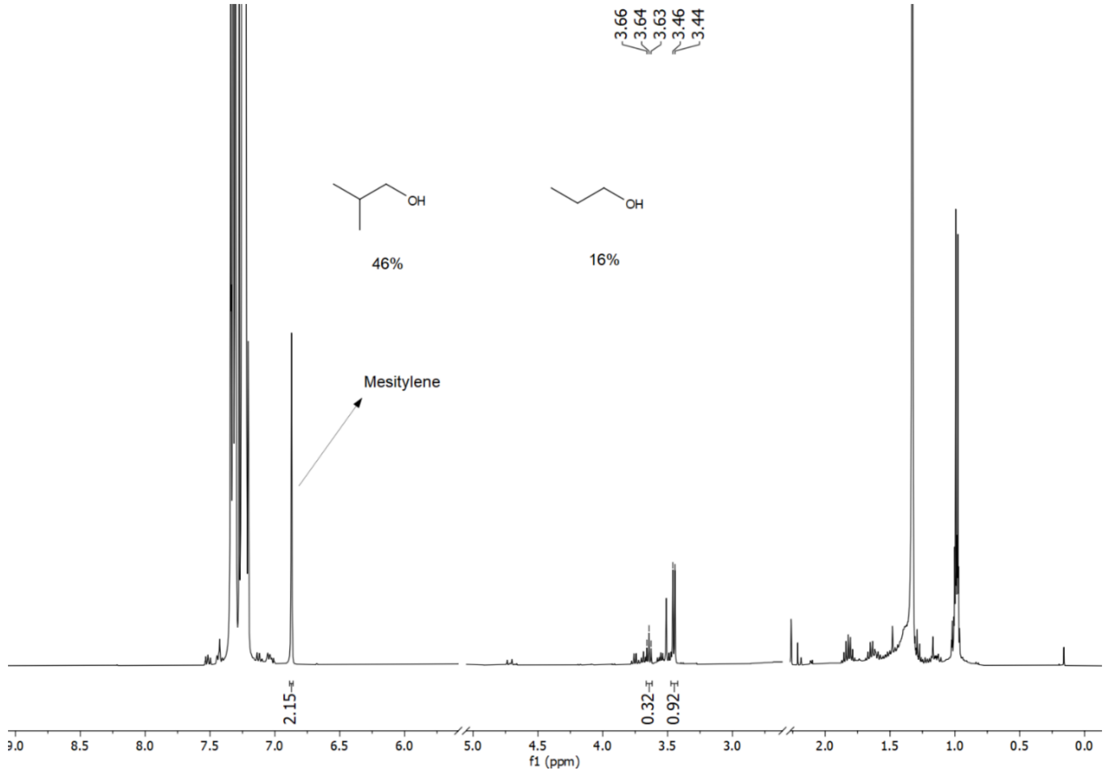

Figure S96: ^{1}H NMR (400 MHz, $CDCl_3$, 298 K) reaction mixture spectrum of 2-methylpropan-1-ol 10a from ethanol.

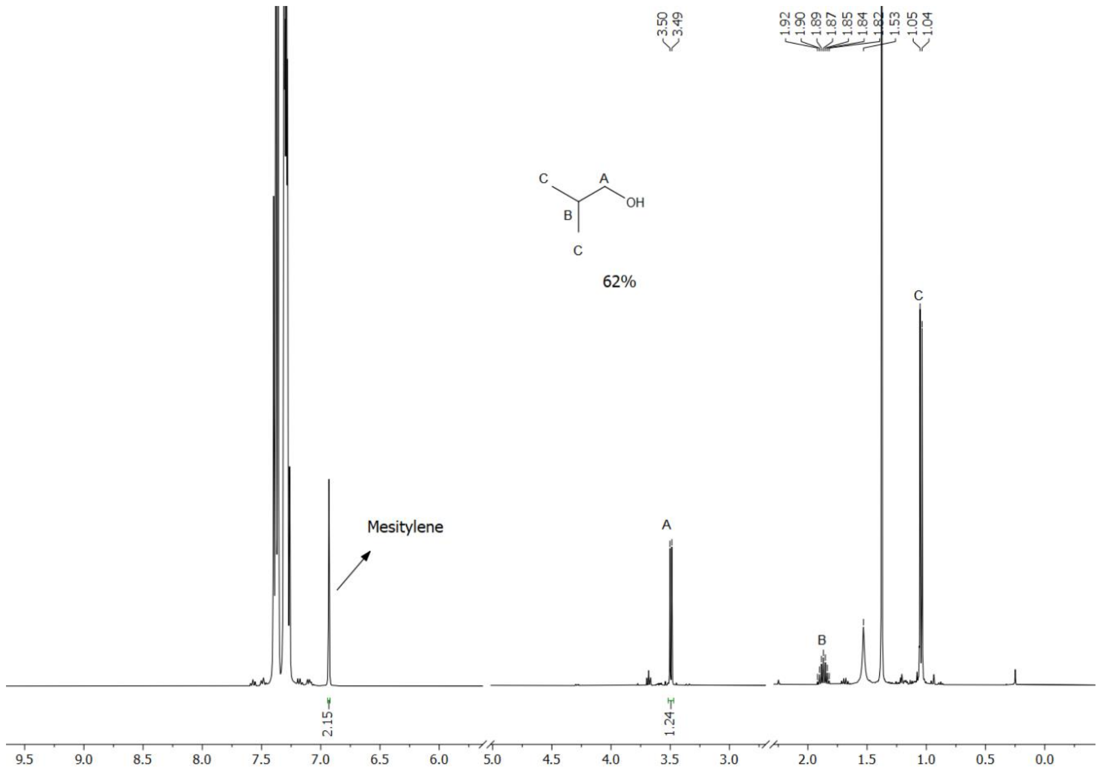

Figure S97: ^{1}H NMR (400 MHz, $CDCl_3$, 298 K) reaction mixture spectrum of 2-methylpropan-1-ol 10a from 1-propanol.

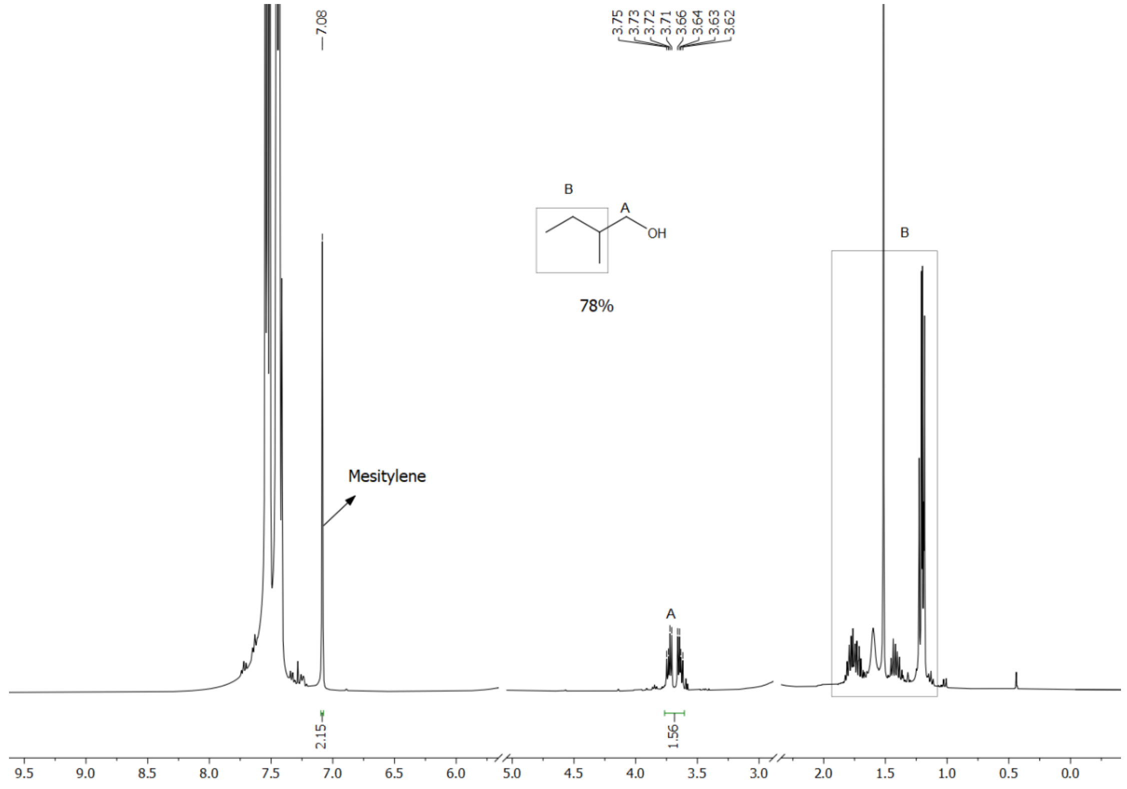

Figure S98:[1]**H NMR (400 MHz, CDCl$_3$, 298 K) reaction mixture spectrum of 2-methylbutan-1-ol 10b.**

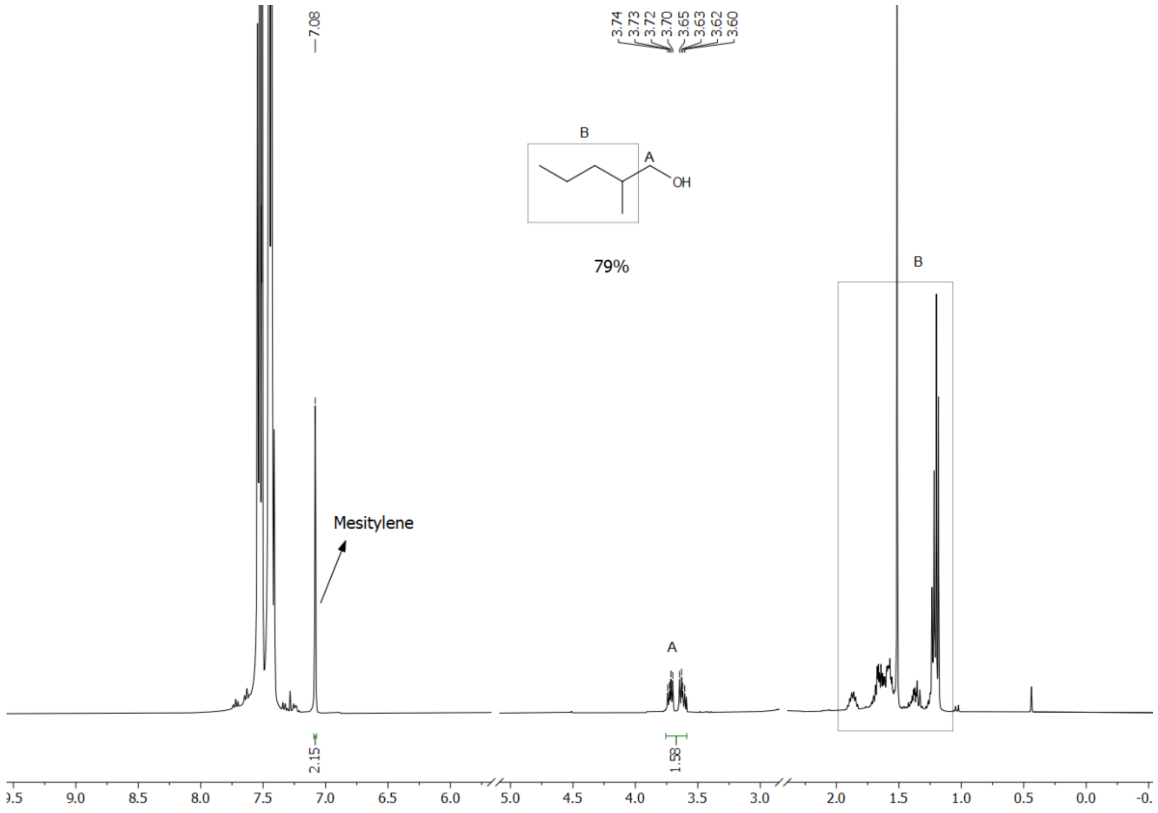

Figure S99:[1]**H NMR (400 MHz, CDCl$_3$, 298 K) reaction mixture spectrum of 2-methylpentan-1-ol 10c.**

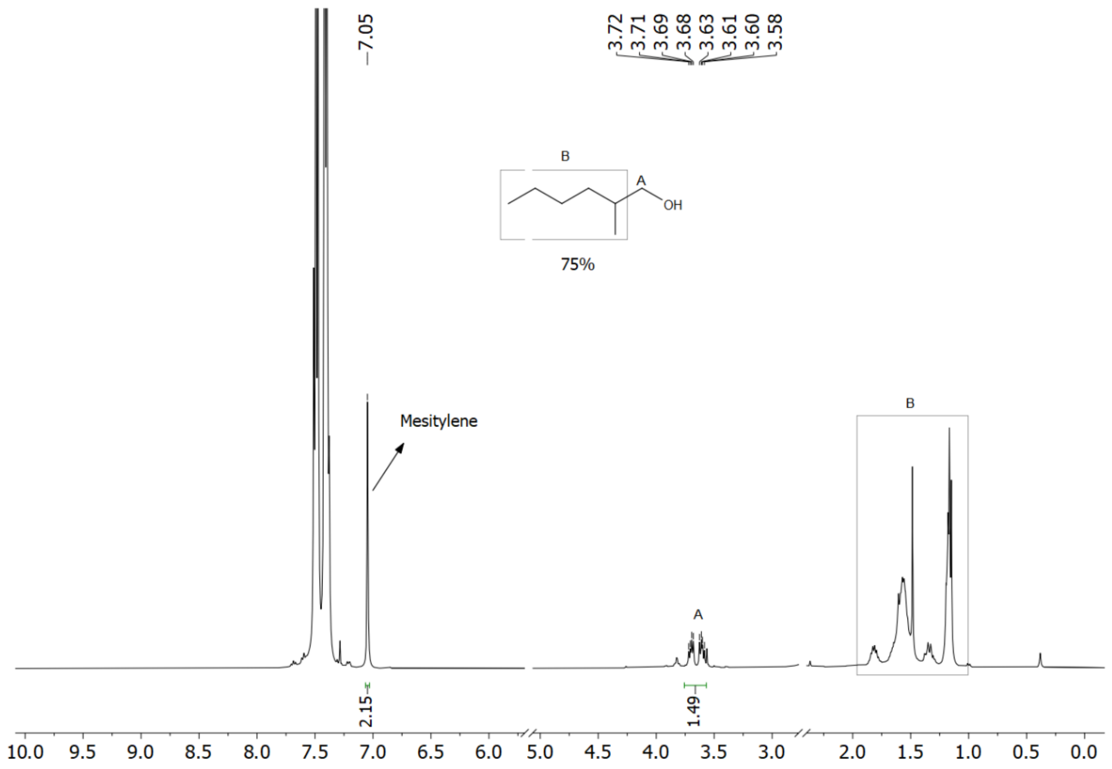

Figure S100: ^{1}H NMR (400 MHz, $CDCl_3$, 298 K) reaction mixture spectrum of 2-methylhexan-1-ol 10d.

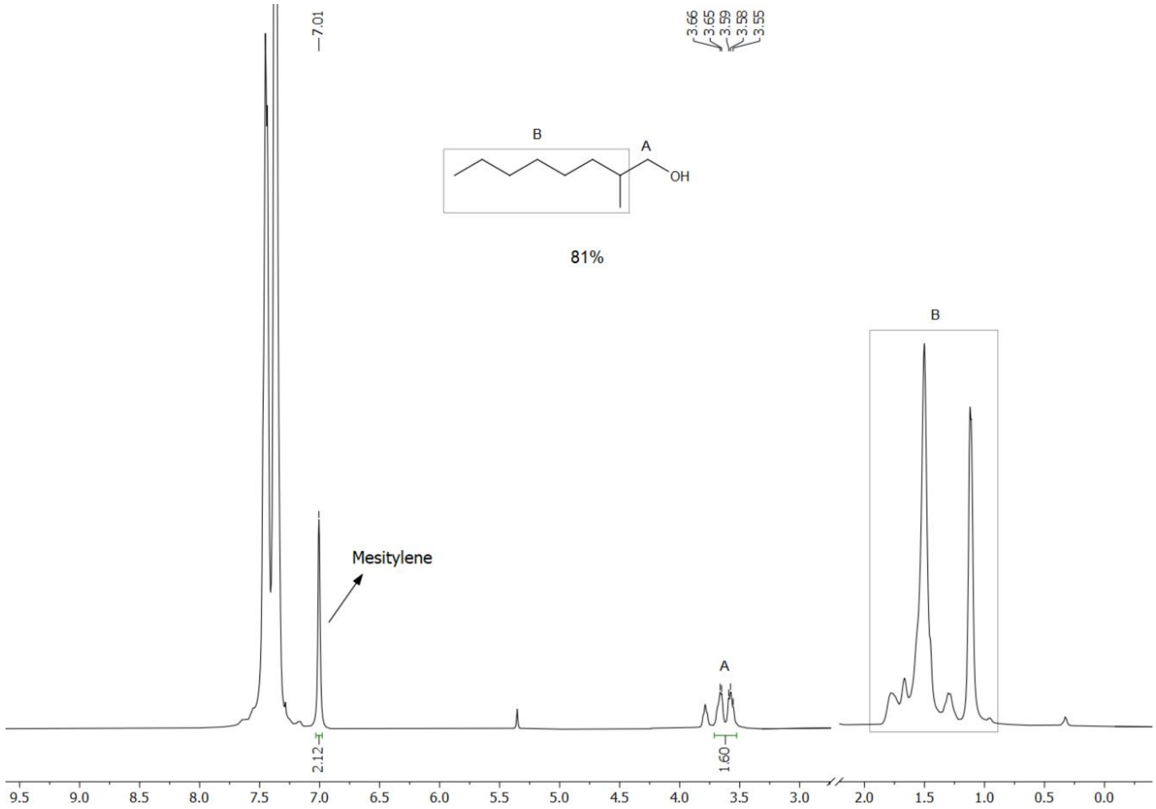

Figure S101:^{1}H NMR (400 MHz, $CDCl_3$, 298 K) reaction mixture spectrum of 2-methyloctan-1-ol 10e.

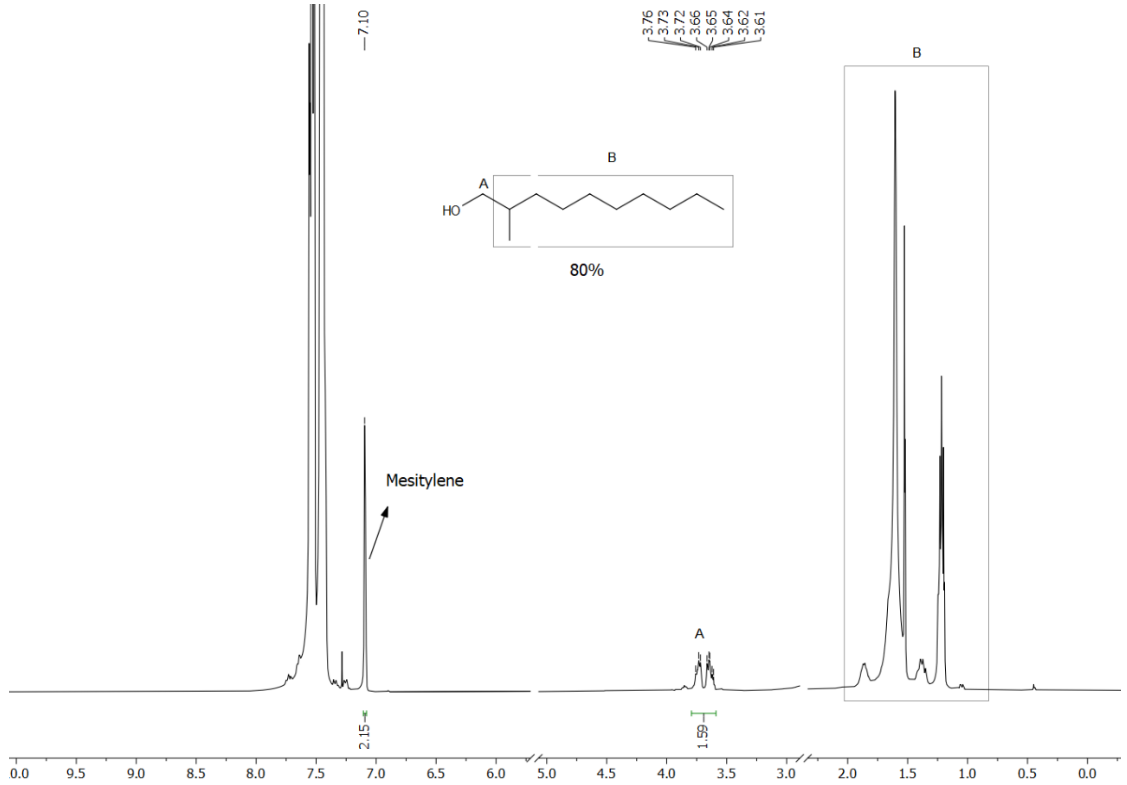

Figure S102: ^{1}H NMR (400 MHz, $CDCl_3$, 298 K) reaction mixture spectrum of 2-methyldecan-1-ol 10f.

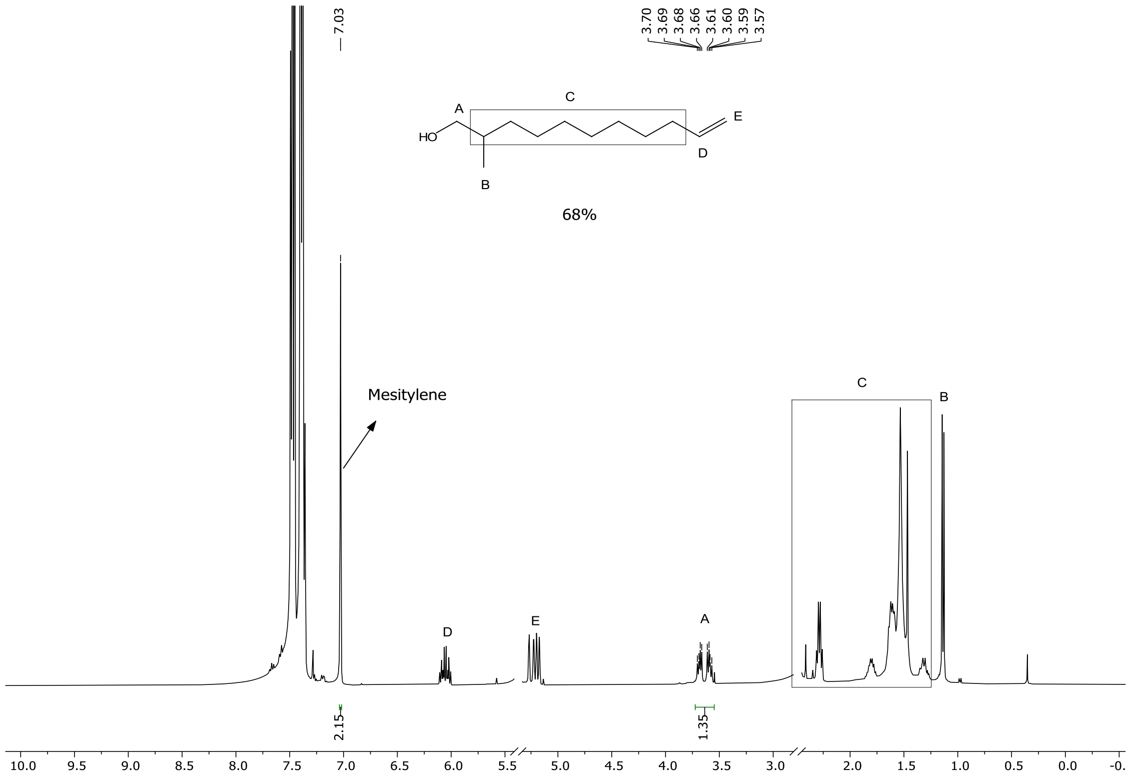

Figure S103: ^{1}H NMR (400 MHz, $CDCl_3$, 298 K) reaction mixture spectrum of 2-methylundec-10-en-1-ol 10g.

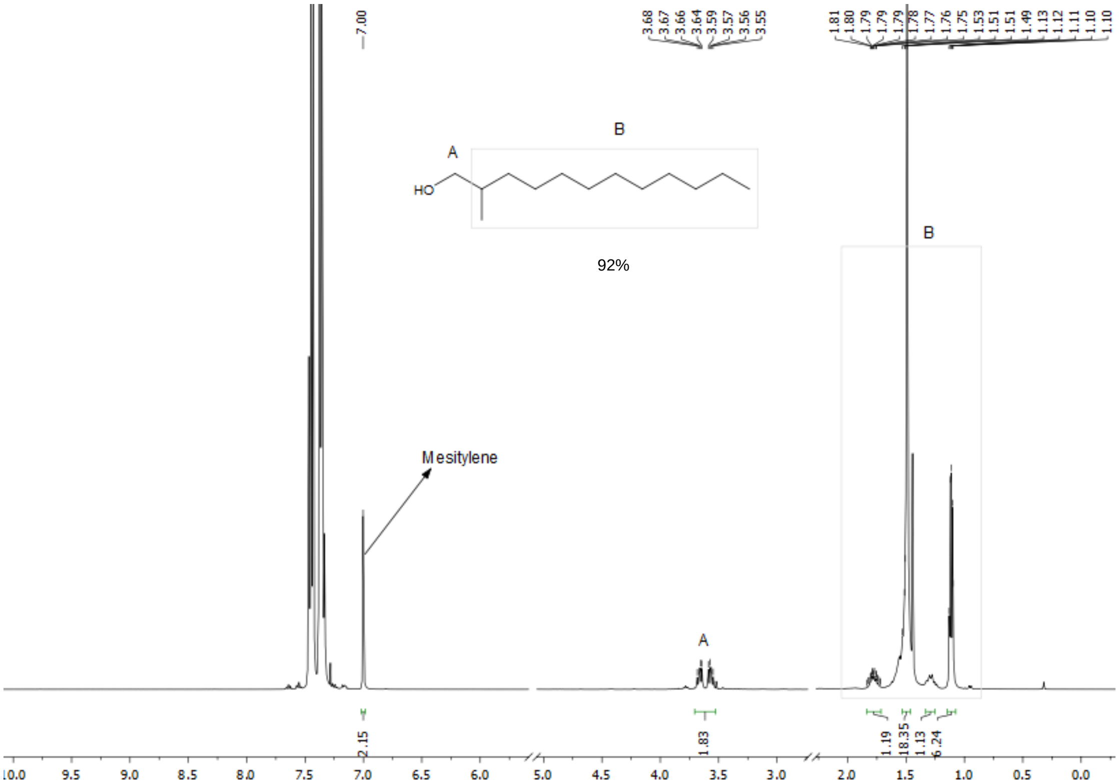

Figure S104: ^{1}H NMR (400 MHz, $CDCl_3$, 298 K) reaction mixture spectrum of 2-methyldodecan-1-ol 10h.

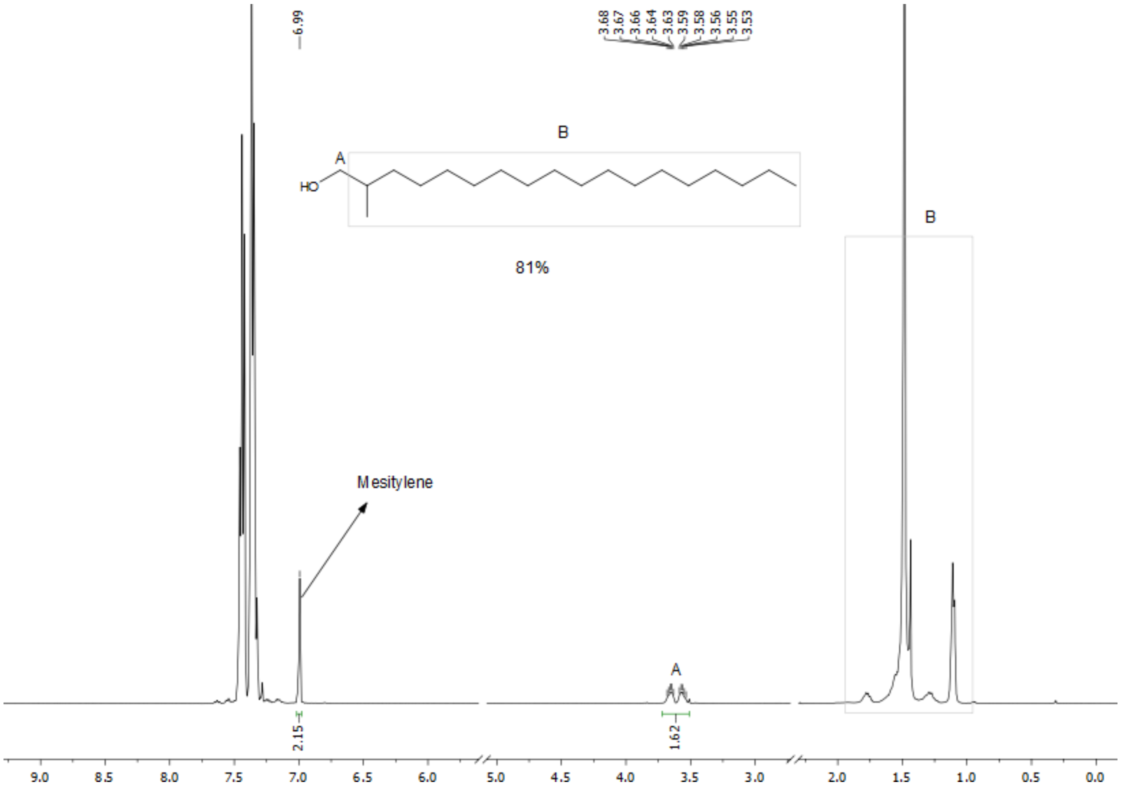

Figure S105: ^{1}H NMR (400 MHz, $CDCl_3$, 298 K) reaction mixture spectrum of 2-methyloctadecan-1-ol 10i.

References

1. G. R. Fulmer, A. J. M. Miller, N. H. Sherden, H. E. Gottlieb, A. Nudelman, B. M. Stoltz, J. E. Bercaw and K. I. Goldberg, *Organometallics*, 2010, **29**, 2176-2179.
2. A. Kaithal, M. Hölscher and W. Leitner, *Angew. Chem. Int. Ed.*, 2018, **57**, 13449-13453.
3. M. Rivlin, U. Eliav and G. Navon, *J. Phys. Chem. B*, 2015, **119**, 4479-4487.
4. S. V. Ley, C. Mitchell, D. Pears, C. Ramarao, J.-Q. Yu and W. Zhou, *Org. Lett.*, 2003, **5**, 4665-4668.
5. K. Oikawa, S. Itoh, H. Yano, H. Kawasaki and Y. Obora, *Chem. Commun.*, 2017, **53**, 1080-1083.
6. J. C. Bardhan and D. N. Mukherji, *Journal of the Chemical Society (Resumed)*, 1956, DOI: 10.1039/JR9560004629, 4629-4633.
7. S. Koul, J. L. Koul, B. Singh, M. Kapoor, R. Parshad, K. S. Manhas, S. C. Taneja and G. N. Qazi, *Tetrahedron: Asymmetry*, 2005, **16**, 2575-2591.
8. A. Gansäuer, M. Klatte, G. M. Brändle and J. Friedrich, *Angew. Chem. Int. Ed.*, 2012, **51**, 8891-8894.
9. Y. Li, H. Li, H. Junge and M. Beller, *Chem. Commun.*, 2014, **50**, 14991-14994.
10. K. Polidano, J. M. J. Williams and L. C. Morrill, *ACS Catal.*, 2019, **9**, 8575-8580.
11. W. J. Jang, S. M. Song, J. H. Moon, J. Y. Lee and J. Yun, *J Am Chem Soc*, 2017, **139**, 13660-13663.
12. Q. Wang, X. Liu, X. Liu, B. Li, H. Nie, S. Zhang and W. Chen, *Chem. Commun.*, 2014, **50**, 978-980.
13. J. E. Roque Pena and E. J. Alexanian, *Org. Lett.*, 2017, **19**, 4413-4415.
14. J. Cao and P. Perlmutter, *Org. Lett.*, 2013, **15**, 4327-4329.
15. A. Kaithal, M. Schmitz, M. Hölscher and W. Leitner, *ChemCatChem*, 2019, **11**, 5287-5291.
16. A. Clerici, A. Ghilardi, N. Pastori, C. Punta and O. Porta, *Org. Lett.*, 2008, **10**, 5063-5066.
17. A. Kišić, M. Stephan and B. Mohar, *Adv. Synth. Catal.*, 2015, **357**, 2540-2546.
18. S. Rodríguez, B. Qu, K. R. Fandrick, F. Buono, N. Haddad, Y. Xu, M. A. Herbage, X. Zeng, S. Ma, N. Grinberg, H. Lee, Z. S. Han, N. K. Yee and C. H. Senanayake, *Adv. Synth. Catal.*, 2014, **356**, 301-307.
19. G. R. Dowson, M. F. Haddow, J. Lee, R. L. Wingad and D. F. Wass, *Angew. Chem. Int. Ed. Engl.*, 2013, **52**, 9005-9008.
20. P. H. Galebach, D. J. McClelland, N. M. Eagan, A. M. Wittrig, J. S. Buchanan, J. A. Dumesic and G. W. Huber, *ACS Sustain. Chem. Eng.*, 2018, **6**, 4330-4344.
21. S. R. Tamang and M. Findlater, *J. Org. Chem.*, 2017, **82**, 12857-12862.
22. I. Burkhardt and J. S. Dickschat, *Eur. J. Org. Chem.*, 2018, **2018**, 3144-3157.
23. P. Wang, D.-L. Wang, H. Liu, X.-L. Zhao, Y. Lu and Y. Liu, *Organometallics*, 2017, **36**, 2404-2411.

24. B. Cao, X. Chen, Y. Yamaryo-Botte, M. B. Richardson, K. L. Martin, G. N. Khairallah, T. W. T. Rupasinghe, R. M. O'Flaherty, R. A. J. O'Hair, J. E. Ralton, P. K. Crellin, R. L. Coppel, M. J. McConville and S. J. Williams, *J. Org. Chem.*, 2013, **78**, 2175-2190.
25. V. N. Kovalenko and I. V. Mineeva, *Russ. J. Org. Chem.*, 2014, **50**, 934-942.
26. R. Ishidate, A. J. Markvoort, K. Maeda and E. Yashima, *J. Am. Chem. Soc.*, 2019, **141**, 7605-7614.
